# Artificial intelligence for the analysis of intracoronary optical coherence tomography images: a systematic review

**DOI:** 10.1093/ehjdh/ztaf005

**Published:** 2025-01-28

**Authors:** Ruben G A van der Waerden, Rick H J A Volleberg, Thijs J Luttikholt, Pierandrea Cancian, Joske L van der Zande, Gregg W Stone, Niels R Holm, Elvin Kedhi, Javier Escaned, Dario Pellegrini, Giulio Guagliumi, Shamir R Mehta, Natalia Pinilla-Echeverri, Raúl Moreno, Lorenz Räber, Tomasz Roleder, Bram van Ginneken, Clara I Sánchez, Ivana Išgum, Niels van Royen, Jos Thannhauser

**Affiliations:** Department of Cardiology, Radboud University Medical Center, Geert Grooteplein Zuid 10, Nijmegen 6525 GA, The Netherlands; Diagnostic Image Analysis Group, Radboud University Medical Center, Geert Grooteplein 10, Nijmegen 6525 GA, The Netherlands; Department of Cardiology, Radboud University Medical Center, Geert Grooteplein Zuid 10, Nijmegen 6525 GA, The Netherlands; Department of Cardiology, Radboud University Medical Center, Geert Grooteplein Zuid 10, Nijmegen 6525 GA, The Netherlands; Diagnostic Image Analysis Group, Radboud University Medical Center, Geert Grooteplein 10, Nijmegen 6525 GA, The Netherlands; Diagnostic Image Analysis Group, Radboud University Medical Center, Geert Grooteplein 10, Nijmegen 6525 GA, The Netherlands; Quantitative Healthcare Analysis (qurAI) Group, Informatics Institute, University of Amsterdam, Amsterdam, The Netherlands; Department of Cardiology, Radboud University Medical Center, Geert Grooteplein Zuid 10, Nijmegen 6525 GA, The Netherlands; Diagnostic Image Analysis Group, Radboud University Medical Center, Geert Grooteplein 10, Nijmegen 6525 GA, The Netherlands; The Zena and Michael A Wiener Cardiovascular Institute, Icahn School of Medicine at Mount Sinai, New York, NY, USA; Department of Cardiology, Aarhus University Hospital, Aarhus, Denmark; McGill University Health Center, Royal Victoria Hospital, Montreal, Canada; Hospital Clinico San Carlos IdISSC, Complutense University of Madrid, Madrid, Spain; U.O. Cardiologia Ospedaliera, IRCCS Ospedale Galeazzi Sant'Ambrogio, Milan, Italy; U.O. Cardiologia Ospedaliera, IRCCS Ospedale Galeazzi Sant'Ambrogio, Milan, Italy; Population Health Research Institute, McMaster University and Hamilton Health Sciences, Hamilton, ON, Canada; Division of Cardiology, Hamilton General Hospital, Hamilton Health Sciences, McMaster University, Hamilton, ON, Canada; Interventional Cardiology, University Hospital La Paz, Madrid, Spain; Department of Cardiology, Bern University Hospital Inselspital, Bern, Switzerland; Faculty of Medicine, Wrocław University of Science and Technology, Wrocław, Poland; Diagnostic Image Analysis Group, Radboud University Medical Center, Geert Grooteplein 10, Nijmegen 6525 GA, The Netherlands; Quantitative Healthcare Analysis (qurAI) Group, Informatics Institute, University of Amsterdam, Amsterdam, The Netherlands; Department of Biomedical Engineering and Physics, Amsterdam University Medical Center University of Amsterdam, Amsterdam, The Netherlands; Quantitative Healthcare Analysis (qurAI) Group, Informatics Institute, University of Amsterdam, Amsterdam, The Netherlands; Department of Biomedical Engineering and Physics, Amsterdam University Medical Center University of Amsterdam, Amsterdam, The Netherlands; Department of Radiology and Nuclear Medicine, Amsterdam University Medical Center University of Amsterdam, Amsterdam, The Netherlands; Department of Cardiology, Radboud University Medical Center, Geert Grooteplein Zuid 10, Nijmegen 6525 GA, The Netherlands; Department of Cardiology, Radboud University Medical Center, Geert Grooteplein Zuid 10, Nijmegen 6525 GA, The Netherlands; Diagnostic Image Analysis Group, Radboud University Medical Center, Geert Grooteplein 10, Nijmegen 6525 GA, The Netherlands

**Keywords:** Optical coherence tomography, Artificial intelligence, Coronary, Intravascular imaging, Systematic review, Deep learning

## Abstract

Intracoronary optical coherence tomography (OCT) is a valuable tool for, among others, periprocedural guidance of percutaneous coronary revascularization and the assessment of stent failure. However, manual OCT image interpretation is challenging and time-consuming, which limits widespread clinical adoption. Automated analysis of OCT frames using artificial intelligence (AI) offers a potential solution. For example, AI can be employed for automated OCT image interpretation, plaque quantification, and clinical event prediction. Many AI models for these purposes have been proposed in recent years. However, these models have not been systematically evaluated in terms of model characteristics, performances, and bias. We performed a systematic review of AI models developed for OCT analysis to evaluate the trends and performances, including a systematic evaluation of potential sources of bias in model development and evaluation.

## Graphical Abstract

**Graphical Abstract:**
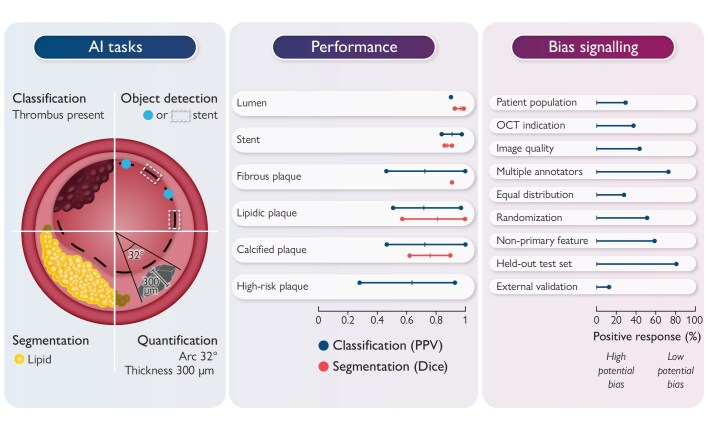
Systematic review on artificial intelligence (AI) tasks in intracoronary optical coherence tomography (OCT): performance and bias signalling. *Left panel:* Typical tasks for AI for the automated assessment of intracoronary OCT images include classification, object detection, and segmentation. Classification can be performed framewise or on individual A-lines and is typically used in a binary fashion (i.e. presence or absence of the feature of interest) or with multiple classes (e.g. lipidic plaque, calcified plaque, fibrous plaque, etc.). Object detection refers to the task of localizing a feature of interest, and is typically performed with bounding boxes (dashed lines) or a centroid (blue dot). Semantic segmentation refers to pixel-wise labelling of the feature(s) of interest. Quantification is frequently performed using post-processing steps after semantic segmentation or A-line classification and includes for example measurements of plaque components. *Middle panel:* Among all identified models, the overall performances (reported as medians with minimum and maximum values) for lumen and stent assessment were excellent, while plaque analysis showed varied results. *Right panel*: The bias signalling questionnaire, containing several questions related to data management, model development, and validation, revealed potential caveats in model training and evaluation that should be addressed in future studies.

## Introduction

The high resolution of intracoronary optical coherence tomography (OCT) enables detailed *in vivo* assessment of coronary morphology, pathology, and implanted stents.^1^ Despite its promise, prevailing guidelines consider the use of OCT only for selective indications.^2,3^ Nevertheless, increasing evidence underscores the value of OCT, particularly for periprocedural guidance of percutaneous coronary intervention (PCI)^4–6^ but also for identification of lesions at risk for future events.^7–10^ Consequently, an increase in clinical use can be foreseen in the near future,^11^ which might result in a shift in end-users from highly trained experts to low-volume operators as well. However, on-site interpretation of the voluminous data available from a single OCT pullback demands a high level of expertise and is time consuming, but is nonetheless pivotal for transforming OCT into an actionable application with clinical value.^12^ To overcome these challenges, automated analysis of OCT frames has been proposed, either through mathematical models or artificial intelligence (AI).

The adoption of AI for medical image analysis has increased substantially in recent years and has proven successful for numerous applications.^13–15^ Artificial intelligence refers to advanced computational systems designed to mimic ‘human intelligence’ and includes both machine learning as well as more sophisticated deep learning methods. Machine learning focuses on specific tasks, enabling computers to learn from data. Deep learning involves advanced neural networks applicable for complex pattern recognition. Typical applications of AI in medical image analysis include tasks such as classification, object detection, segmentation, and regression, which can be used for the quantification of coronary morphology and/or stents (Graphical Abstract). Specifically for OCT, these tasks can be clinically useful for applications such as segmentation of the lumen (e.g. for stent sizing), detection of stent struts (e.g. for assessing malapposition), classification of plaque morphology (e.g. for identifying thin-cap fibroatheroma), and regression analysis of OCT-derived fractional flow reserve (FFR). Moreover, AI models could be investigated for the prediction of clinical or procedural outcomes.

Artificial intelligence models targeted for clinical use require trustworthy development and validation, in order to avoid potential bias, to ensure generalizability, and to achieve robust performance.^16,17^ We, therefore, performed a systematic review to provide a comprehensive overview of existing AI models employed in OCT analysis. We analysed trends in this application area, focusing on different tasks and performances of currently proposed methods. Moreover, we aimed to assess potential sources of bias across reported AI models, and highlighted the next steps required for clinical adoption of such models.

## Methods

### General information

This systematic review was prospectively registered in PROSPERO (CRD42023441903) and the manuscript is reported in accordance with the Preferred Reporting Items for Systematic Reviews and Meta-Analyses (PRISMA) statement.^18^  *Table 1* provides definitions of commonly used terms.

**Table 1:** Definitions

Term	Definition
A-line	Individual axial scan line.
Artificial intelligence	Advanced computer systems designed to mimic human intelligence.
Cartesian co-ordinates	Cross-sectional representation of an OCT image.
*Datasets*	
Training set	Subset of data used to train an AI model, providing examples for the algorithm to learn patterns and features from.
Validation set	Separate subset of data used to fine-tune and optimize the parameters of the model during the training process.
Held-out test set	Independent set of data, derived from the internal data source, not seen by the algorithm during training or validation, used to assess the performance and generalization ability of the trained model.
External test set	Independent set of data, derived from a different data source, not seen by the algorithm during training or validation, used to assess the performance and generalization ability of the trained model.
Deep learning	Part of machine learning that involves advanced neural networks applicable for complex pattern recognition.
Dice score	Performance metric used for segmentation tasks, calculated as two times the area of overlap divided by the total area of the class of interest in the prediction and reference standard.
Machine learning	AI subset enabling systems to learn from data.
Polar co-ordinates	Representation of an OCT image with the angle (*θ*) and radius (*r*).
Quantification	The precise measurement and assessment of characteristics such as plaque angle measurement, mostly using post-processing.
*Tasks*	
Classification	AI task that aims to categorize input features into output classes, which can be dichotomous (e.g. present vs. absent) or multiclass (e.g. lipid vs. calcified vs. fibrous).
Object detection	AI task that aims to localize the feature of interest using for example bounding boxes or centroids.
Regression	AI task that aims to predict a continuous variable.
Segmentation	AI task that aims to pixel-wise label the input image.

AI, artificial intelligence; OCT, optical coherence tomography.

### Search strategy and selection criteria

A systematic electronic literature search was performed on Embase, Pubmed, Scopus, and Web of Science for papers that use AI for intracoronary OCT. The final search was performed on 31 December 2023. Search terms included; ‘Artificial Intelligence’, ‘Optical Coherence Tomography’, and ‘Coronary Vessels’ or synonyms. The exact search queries can be found in Supplementary material online, *Table S1*. We included articles which (i) used intracoronary OCT, (ii) employed AI models based on machine learning or deep learning, and (iii) focused on human coronary arteries, either *in vivo* or *ex vivo*. The main exclusion criteria were (i) sole use of non-AI models, e.g. mathematical models, (ii) conference abstracts or proceedings, (iii) non-English articles, (iv) articles unavailable for full-text analysis, and (v) articles not reporting exact performance metrics and/or outcome measures.

After the removal of duplicates, titles and abstracts were screened for potential inclusion. Full-text articles were subsequently evaluated for final eligibility.

### Study evaluation and data extraction

We systematically abstracted data on study characteristics, AI model characteristics (e.g. machine learning, deep learning, etc.), AI-specific tasks (e.g. classification, segmentation, etc.), outcome measures, and performance metrics. Performance metrics of interest included accuracy, sensitivity, specificity, positive predictive value (PPV), negative predictive value (NPV), F1 score, Dice and Jaccard scores. The best-performing algorithm was included when multiple algorithms were tested for the same task within a single study. Screening and data extraction were performed independently by two investigators (R.W. and R.V.). Cases of discrepancies were resolved through discussion with a third investigator (J.T.). The full study evaluation form can be found in Supplementary material online, *File S1*.

### Bias signalling assessment

Contemporary bias assessment tools employed for systematic reviews on diagnostic accuracy studies (e.g. QUADAS-2),^19^ are considered less suitable to identify AI-specific biases.^20^ Important sources of bias in AI studies include data collection, data preparation, model training, and model evaluation.^21,22^ To the best of our knowledge, only one AI-specific bias assessment tool is published, that primarily involves a subjective evaluation of articles.^23^ Therefore, we evaluated each article using nine objective bias signalling questions, each one linked to a specific source of bias within AI (*Table 2*).^21,22^ All questions were answered with yes (+), no (−), or unknown (?). Questions were independently evaluated by two investigators (R.W. and R.V.), and discrepancies were resolved through discussion with a third investigator (J.T.).

**Table 2:** Bias signalling questions

	Category	Keyword	Question
	**Data collection**		
**1**	Population bias	Patient population	Is a description available of the **patient population**?
**2**	Population bias	OCT indication	Is a description available of the **indication for OCT** and/or the type of lesions?
**3**	Representation bias	Image quality	Is information available of the management of **image quality**/artefacts?
	**Data preparation**		
**4**	Annotator bias	Multiple annotators	Does the annotation procedure include **multiple annotators**?
	**Model training**		
**5**	Sampling bias	Equal distribution	Are the primary feature(s) of interest **equally distributed** between the training and test set?^a^
**6**	Sampling bias	Randomization	Are the training and test set **randomized**/split on either a patient or pullback-level (i.e. are frames of the same pullback not present in both training and test set)?
**7**	Training data bias	Non-primary feature	Do the training and test set include samples without the **primary feature(s) of interest**?
	**Model evaluation**		
**8**	Evaluation bias	Held-out test set	Is a **held-out test** set used for model evaluation?
**9**	Population bias	External validation	Is the held-out test set obtained from an **external cohort**?

^a^Defined as a relative difference ≤20%.

OCT, optical coherence tomography.

## Results

### Study selection

**Figure 1:**
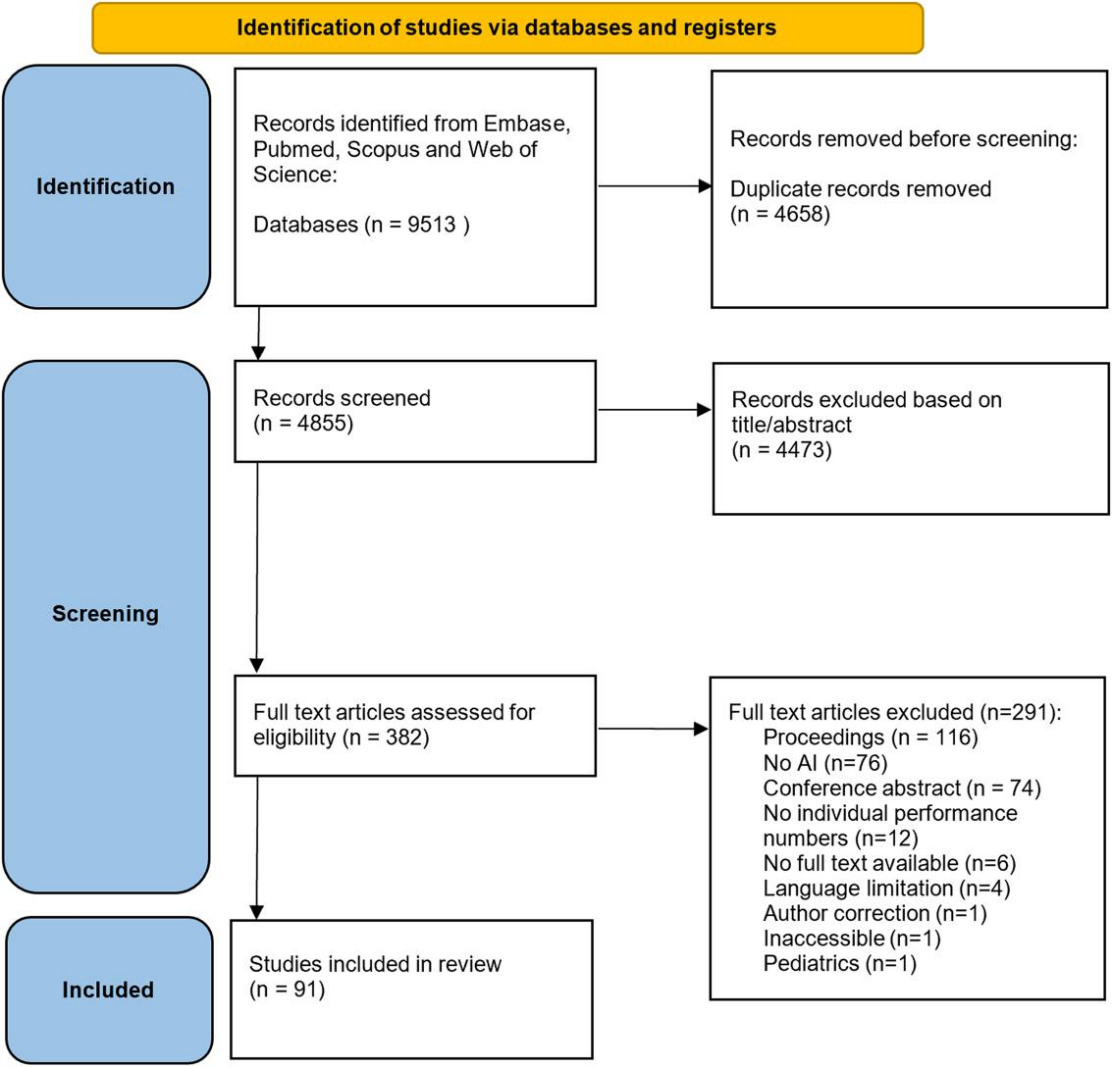
Flowchart of study selection.

A total of 9513 articles were identified, of which 4855 remained after removal of duplicates (*Figure 1*). Subsequently, 4473 articles were excluded based on title and abstract screening. Among the 382 remaining articles, 291 were excluded based on full-text evaluation. Finally, 91 articles were included for analysis.

### Study characteristics

The clinical scenario in which OCT was performed was specified in 19 (21%) articles, including six (32%) that solely included patients presenting with acute coronary syndromes (ACSs), and four (21%) with chronic coronary syndromes, whilst nine (47%) included patients with either. The number of patients included among the articles ranged from 1 to 1791 [median 41 (interquartile range, IQR 15–67)]. Sex was reported in 21 (23%) articles with the proportion of female patients ranging from 15% to 100% [median 25% (IQR 22–31)]. Studies were predominantly conducted *in vivo* [86 articles, (95%)] and histology served as the reference standard in four (4%) articles. Individual study and patient characteristics are reported in Supplementary material online, *Tables S2* and *S3*.

### Model characteristics and tasks

**Figure 2:**
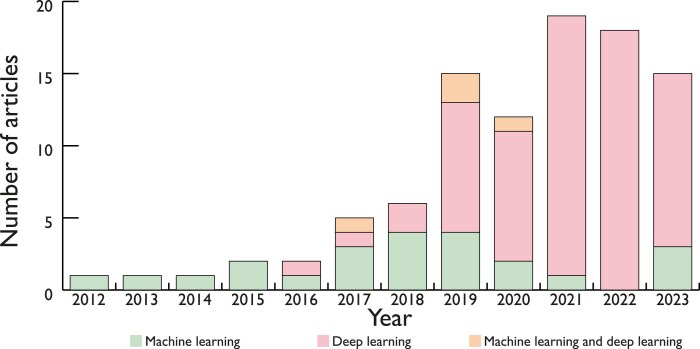
Annual publications on machine learning and deep learning.

**Figure 3:**
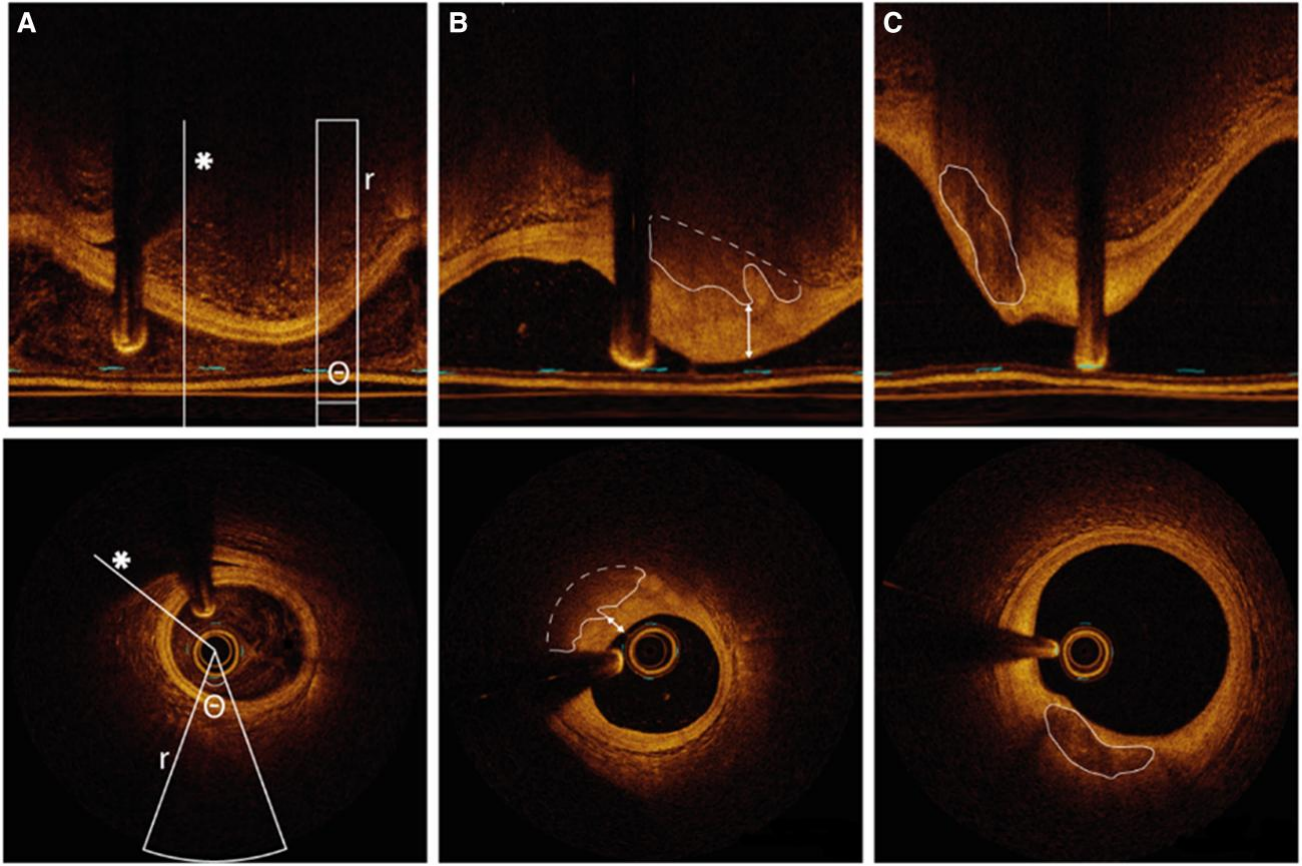
Examples of intracoronary optical coherence tomography images in polar and Cartesian views. The top row presents three optical coherence tomography frames in polar view, while the reconstructed Cartesian view is displayed in the bottom row. (*A*) Polar and Cartesian view of the same frame illustrating the *y*-axis aligning with radius (*r*) and the *x*-axis corresponding to the angle (Θ). Corresponding A-lines are denoted by an asterisk. (*B*) Example of an optical coherence tomography frame with a lipidic plaque with a thick fibrous cap (arrows). The inner boundary of the lipidic plaque is outlined with a continuous line, while the estimated outer boundary is represented by a dashed line. (*C*) Example of an optical coherence tomography frame with a calcified plaque (white outline).

**Figure 4:**
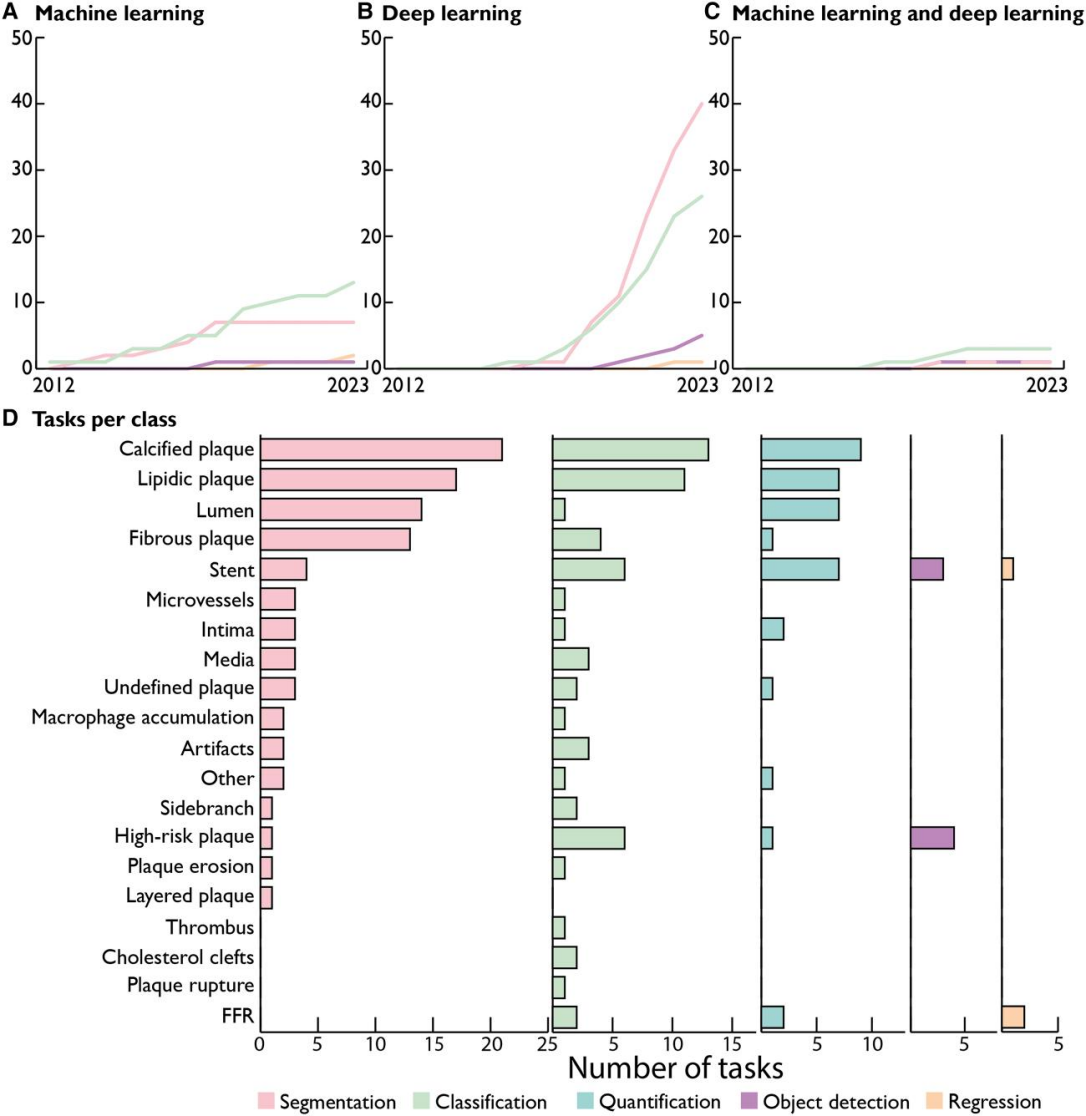
Distribution of tasks per class. Cumulative number of identified tasks for segmentation, classification, object detection, and regression over time (*A–C*) and per class (*D*). Quantification is not an artificial intelligence task but refers to transforming the model output into numerical variables to quantify for example plaque volume or fractional flow reserve.

A total of 97 models were described. Model characteristics are provided in Supplementary material online, *Table S4*. A notable shift in different AI methods was observed over time, with sole development of machine learning methods between 2012 and 2015, followed by an increasing adoption of deep learning methods (*Figure 2*). Thirty-six (37%) models analysed OCT frames in polar co-ordinates, 34 (35%) used Cartesian co-ordinates, and three (3%) used a combination of both (*Figure 3A*). Additionally, 80 models (82%) used a 2D analysis approach (i.e. single frame input). Among the 97 models, 100 distinct tasks were identified, predominantly segmentation [48 tasks, (48%)] and classification [42 tasks, (42%)]. Other AI tasks included object detection [7 tasks, (7%)] and regression [3 tasks, (3%)] (*Figure 4A–C*). Most tasks focused on the assessment of atherosclerotic plaques, the coronary lumen, and stent struts (*Figure 4D*). Additionally, 19 models (20%) performed a subsequent quantitative evaluation of the primary feature of interest. The algorithm code was shared with the publication in two articles (2%), while data and code are available upon request for 27 (30%) and one (1%) articles, respectively.

### Model performances

Overall and individual model performances are reported in *Table 3* and Supplementary material online, *Tables S5*–*S24*, respectively. Results are presented as median (minimum–maximum values). The median Dice score across all studies for segmentation of the lumen was 0.972 (0.933–0.988). The mean difference between the AI-based lumen area and the reference standard was <0.50 mm^2^ in seven of the eight (87.5%) studies reporting on the quantification of the lumen (see Supplementary material online, *Table S25*). Segmentation of the media was reported in two studies with one study reporting a Dice score of 0.9108 and the other a pixel-level accuracy of 87%.

**Table 3:** Overview of model performances

CLASS/TASK	ACC (%)	SENS (%)	SPEC (%)	PPV (%)	NPV (%)	F1 (%)	AUC–ROC	Dice	Jaccard
Lumen									
Classification	98.2	95.9	98.5	90.0	99.4	—	0.96	—	—
Segmentation	98.1 (98.0–98.2)	94.5 (90.6–99.0)	98.80 (93.0–99.7)	96.9	—	97.2 (95.4–99.0)	—	0.97 (0.93–0.99)	0.92 (0.88–0.97)
Intima									
Classification	92.0	90.0	94.0	—	—	—	—	*—*	*—*
Segmentation	90.0	96.0 (93.0–98.9)	86.0	98.9	—	99.0	—	0.95 (0.91–0.99)	—
Media									
Classification	92.0	94.0	90.0	—	—	—	—	—	—
Segmentation	87.0	91.0	82.0	—	—	99.0	—	0.91	—
Sidebranch									
Classification	95.7 (93.4–97.9)	98.3 (98.1–98.5)	79.2 (60.1–98.4)	96.1 (94.7–97.5)	—	98.0 (96.3–99.7)	0.97 (0.94–1.00)	—	—
Segmentation	—	89.0	—	81.5	—	—	—	0.85	—
Lipidic plaque									
Classification	90.6 (75.4–99.2)	85.6 (67.0–100)	91.1 (78.1–99.0)	71.5 (50.6–97.1)	91.9 (85.0–95.9)	81.2 (57.9–98.5)	0.90 (0.82–0.98)	—	—
Segmentation	88.0 (74.1–96.4)	81.0 (71.0–99.0)	91.9 (82.9–96.0)	81.2 (68.0–91.8)	95.0 (94.3–95.7)	80.1 (62.0–92.7)	0.86 (0.85–0.86)	0.81 (0.57–1.00)	0.69 (0.45–0.99)
High-risk plaque									
Classification	93.2 (90.0–96.7)	92.2 (88.6–94.1)	90.9 (84.2–93.2)	63.3 (28.0–92.9)	99.0 (99.0–99.0)	92.9 (92.2–93.7)	0.96 (0.92–1.00)	—	—
Detection	71.6	93.7 (88.0–95.0)	—	84.2 (83.3–88.8)	—	—	0.87	—	—
Segmentation	93.3	91.4	94.3	—	—	—	—	—	—
Calcified plaque									
Classification	92.8 (73.4–100)	84.0 (37.6–100)	95.3 (85.4–100)	72.5 (46.1–100)	97.0 (80.4–99.4)	88.3 (41.4–100)	0.97 (0.93–1.00)	—	—
Segmentation	91.1 (72.1–99.0)	87.4 (67.8–92.9)	92.0 (76.4–99.0)	83.0 (65.9–95.7)	89.0 (80.6–93.1)	78.1 (66.8–93.6)	0.90 (0.89–0.91)	0.76 (0.62–0.90)	0.59 (0.34–0.83)
Fibrous plaque									
Classification	94.0 (81.6–96.0)	94.0 (55.0–94.0)	96.0 (90.5–99.0)	65.8	85.8	59.9	—	—	—
Segmentation	93.0 (81.9–100)	88.6 (74.0–95.0)	94.0 (85.0–98.6)	86.3 (73.0–96.0)	95.0 (85.0–98.6)	88.7 (83.0–94.4)	0.85	0.91	0.58 (0.34–0.81)
Plaque rupture									
Classification	—	81.7 (74.3–83.3)	93.8 (84.9–96.4)	70.6 (44.6–96.0)	89.2 (67.5–99.0)	—	0.94 (0.90–0.98)	—	—
Plaque erosion									
Classification	—	84.9 (80.0–92.7)	84.5 (73.4–88.5)	69.7 (56.7–80.2)	94.8 (86.1–98.7)	—	0.92 (0.90–0.94)	—	—
Layered plaque									
Classification	76.8 (76.0–77.6)	77.1 (76.5–77.7)	76.8 (76.0–77.6)	—	—	—	0.85 (0.85–0.86)	—	—
Segmentation	—	67.0	96.0	53.0	96.0	—	0.86	—	0.36
Undefined plaque									
Classification	81.7 (74.7–91.7)	87.8 (53.8–90.9)	84.0 (61.5–92.4)	52.9	84.5	72.4 (53.4–91.3)	—	—	—
Segmentation	93.0 (90.2–96.0)	90.0 (76.3–97.0)	95.0 (93.5–95.0)	74.0	94.3	84.0 (75.1–96.0)	—	—	—
Thrombus									
Classification	98.0	97.0	99.0	—	—	—	—	—	—
Macrophages									
Classification	92.0	89.0	97.0	—	—	—	—	—	—
Segmentation	48.1	56.8	42.9	—	—	—	—	0.49	—
Cholesterol clefts									
Segmentation	94.7	50.8	—	54.3	—	—	—	0.53	—
Microvessels									
Classification	92.5 (90.0–95.0)	85.0 (80.0–90.0)	98.5 (97.0–100)	—	—	—	—	—	—
Segmentation	—	64.4 (60.4–85.5)	99.8	59.8	—	—	—	0.60 (0.55–0.73)	—
Stents									
Classification	89.7 (82.8–96.5)	93.9 (87.4–95.8)	90.0	90.1 (84.0–97.5)	—	—	0.97	—	—
Detection	—	95.4 (91.5–96.0)	87.9	98.7 (97.9–99.5)	—	93.3 (90.0–96.6)	—	—	—
Segmentation	—	92.0 (90.0–96.6)	92.2 (90.4–94.0)	92.9 (92.0–94.3)	—	92.3 (91.0–93.6)	—	0.88 (0.86–0.91)	0.84
Regression	84.0	87.0	82.0	—	—	—	0.85	—	—
Fractional flow reserve									
Classification	83.2 (77.5–91.7)	89.6 (72.9–98.3)	70.6 (61.5–81.5)	85.7 (77.8–92.1)	77.4 (77.2–88.9)	—	0.76	—	—
Regression	85.4 (75.5–95.2)	87.1 (74.1–100)	85.0 (77.1–92.9)	83.0 (78.4–87.5)	86.3 (72.5–100)	—	0.90 (0.82–0.98)	—	—
Artefacts									
Classification	88.6 (81.2–95.9)	94.8 (90.6–99.0)	93.4 (87.1–99.6)	65.2 (34.7–95.1)	99.6 (99.2–99.9)	98.4	0.98 (0.96–1.00)	—	—
Segmentation	99.3	94.6 (92.0–97.1)	99.9	93.3 (87.0–99.6)	99.3	98.4	—	0.89	—

Overall model performances according to the different classes and tasks. Results are reported as median (minimum–maximum), or as a single value in case of a single article presenting the specific performance metric.

ACC, accuracy; AUC–ROC, area under the receiver operating characteristic curve; NPV, negative predictive value; PPV, positive predictive value; SENS: sensitivity; SPEC, specificity.

Lipidic plaque classification resulted in a median accuracy of 90.6% (75.4–99.2%) with a median sensitivity of 85.6% (67–100%) and PPV of 71.5% (50.6–97.1%). For calcified plaque classification, the median accuracy was 92.8% (73.4–100%), the median sensitivity was 84% (37.6–100%), and the median PPV was 72.5% (46.1–100%), whilst the median accuracy, sensitivity and PPV for classification of fibrous plaques were 94% (81.6–96%), 94% (55–94%), and 65.8% (reported in one article), respectively. The median Dice scores for segmentation of lipid and calcified plaques were 0.814 (0.565–0.995) and 0.756 (0.618–0.897), respectively. Quantification of calcified (e.g. calcium arc) or lipidic plaques [e.g. minimum fibrous cap thickness (FCT) or lipid arc] was performed in nine and seven articles, respectively (see Supplementary material online, *Table S25*). The median accuracy for the classification of high-risk plaques was 93.2% (90–96.7%) with a sensitivity of 92.2% (88.6–94.1%) and PPV of 63.3% (28.0–92.9%). Using detection methods for high-risk plaque assessment, the median sensitivity and PPV were 93.7% (88.0–95.0%) and 84.2% (83.3–88.8%), respectively. Only four and three studies compared the model prediction for lipid and calcified plaque to histology, respectively (see Supplementary material online, *Tables S9* and *S11*).

Classification of plaque rupture and plaque erosion were reported in only one and two studies, respectively (see Supplementary material online, *Tables S13* and *S14*). The median reported sensitivities for plaque rupture and plaque erosion were 81.7% (74.3–83.3%) and 84.9% (80–92.7%), respectively.

The median sensitivity and PPV for the classification of stent struts, mostly performed and evaluated on a strut level, were 93.9% (87.4–95.8%) and 90.1% (84–97.5%), respectively. The median correlation of the predicted stent area and malapposition distance with the reference standard was 0.962 (0.918–0.990) and 0.99 (0.98–1.00), respectively (see Supplementary material online, *Table S25*).

Two papers reported on a model for the classification of lesions with a pressure wire-derived FFR of ≤0.80 vs. >0.80 (see Supplementary material online, *Table S22*). The median accuracy was 83.2% (77.5–91.7%), the median sensitivity was 89.6% (72.9–98.3%), and the median PPV was 85.7% (77.8–92.1%). Two papers described a regression model for the prediction of the OCT-derived FFR value and reported a median accuracy of 85.4 (75.5–95.2%) and a median area under the receiver operating characteristic curve of 0.90 (0.82–0.98) to identify lesions with a pressure wire-derived FFR ≤0.80 vs. >0.80.

### Histological validation

Among four studies with histology-based validation of the AI methods, one performed A-line-based classification of lipidic plaques (A-line sensitivity 83.6% and specificity 91.1%). In the three studies on semantic segmentation of lipidic, fibrous, calcified, and/or layered plaques, reported pixel-wise accuracies were 87.7%, 97.4%, 97.6%, and unreported. Median Jaccard indices were 0.57 (0.45–0.68), 0.34, 0.43 (0.34–0.51), and 0.36, respectively.

### Prognosis

Associations between AI-detected plaque features and either stent-related, plaque-related, or clinical outcomes were reported in one, two, and three studies, respectively (see Supplementary material online, *Table S26*). As for the latter, high-risk plaques as defined in the respective studies were associated with an increased risk of study-defined clinical events in all three studies, with a median hazard ratio of 2.56 (1.87–19.13) on a patient level and 12.12 (10.72–13.53) on a lesion-level.

### Bias signalling questions

**Figure 5:**
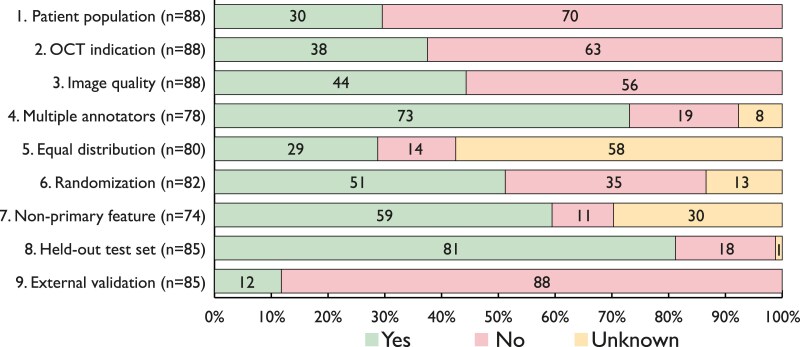
Results of bias signalling questions.

The bias signalling questions were evaluated in 88 articles (*Figure 5*, Supplementary material online, *Table S27*), excluding three articles that did not report a new model. The patient population characteristics and indication for OCT were described in 26 (30%) and 33 (38%) articles, respectively, with 39 (44%) articles reported on their approach to manage suboptimal frame quality. Sixty-nine (81%) articles evaluated their model on a held-out test set, while 10 (12%) articles used an external test set.

### Inference time and software packages

Inference times were reported for 15 models. For classification tasks, the median inference times were 0.385 s (0.003–16) at the frame-level, and 60 s (0.025–605) at the pullback-level. For segmentation tasks, the median inference times were 0.1725 s (0.0044–30) at the frame-level and 37.7 s (0.366–120) at the pullback-level.

A total of five software packages were identified in the included studies. Of these, one focused on calcified plaque segmentation, one on stent analysis, and three on plaque and stent assessment.

## Discussion

This systematic review provides a comprehensive overview of current AI models for the analysis of intracoronary OCT frames, highlighting the growing interest and increasing use of AI for intracoronary OCT analysis in recent years. Most reported models have targeted assessment of coronary lumen, plaques, and stents, with some also allowing quantification of these structures. The described models demonstrate the feasibility of AI for automated analysis of the extensive data generated by a single OCT pullback for several tasks, including segmentation, classification, and object detection. Specifically, excellent overall performances were reported for lumen and stent evaluation, while more varied performances were reported for plaque analysis.

Despite these efforts, only a limited number of software packages were identified, and none of the proposed models was clinically evaluated in a randomized controlled trial. Additionally, this overview highlights potential bias-related caveats in model training and evaluation that should be prioritized in future studies. In particular, data presentation among articles was diverse, and important information was frequently unavailable. These considerations are essential for ensuring the robustness of AI solutions for intracoronary OCT and to chart a pathway for their clinical adoption.

### Evolution of artificial intelligencey models for optical coherence tomography analysis

Since the 1990s, AI models have increasingly been applied for medical image analysis, with the first AI model for intracoronary OCT appearing in 2012, which targeted stent strut classification using machine learning.^24^ Recently, there has been a notable shift towards more sophisticated deep learning approaches. This transition has aligned with a revolution of AI-related tasks, shifting from relatively simple classification tasks to more intricate studies involving pixel-wise semantic segmentation of OCT frames, and detection of high-risk plaques. It should be acknowledged that while mathematical models have also been proposed,^25,26^ these fall outside the scope of the present review.

### Periprocedural guidance of revascularization

Multiple randomized trials have compared intravascular imaging to angiography for guidance of coronary revascularization. The totality of evidence indicates significant clinical benefit with intravascular guidance particularly for complex coronary lesions.^4–6^ Notably, real-world OCT-guidance has been shown to result in altered decision-making in over 80% of lesions undergoing PCI, highlighting that real-time accurate OCT interpretation is pivotal.^27^ The findings of this systematic review suggest that AI can serve as a valuable tool in various stages of this process.

**Figure 6:**
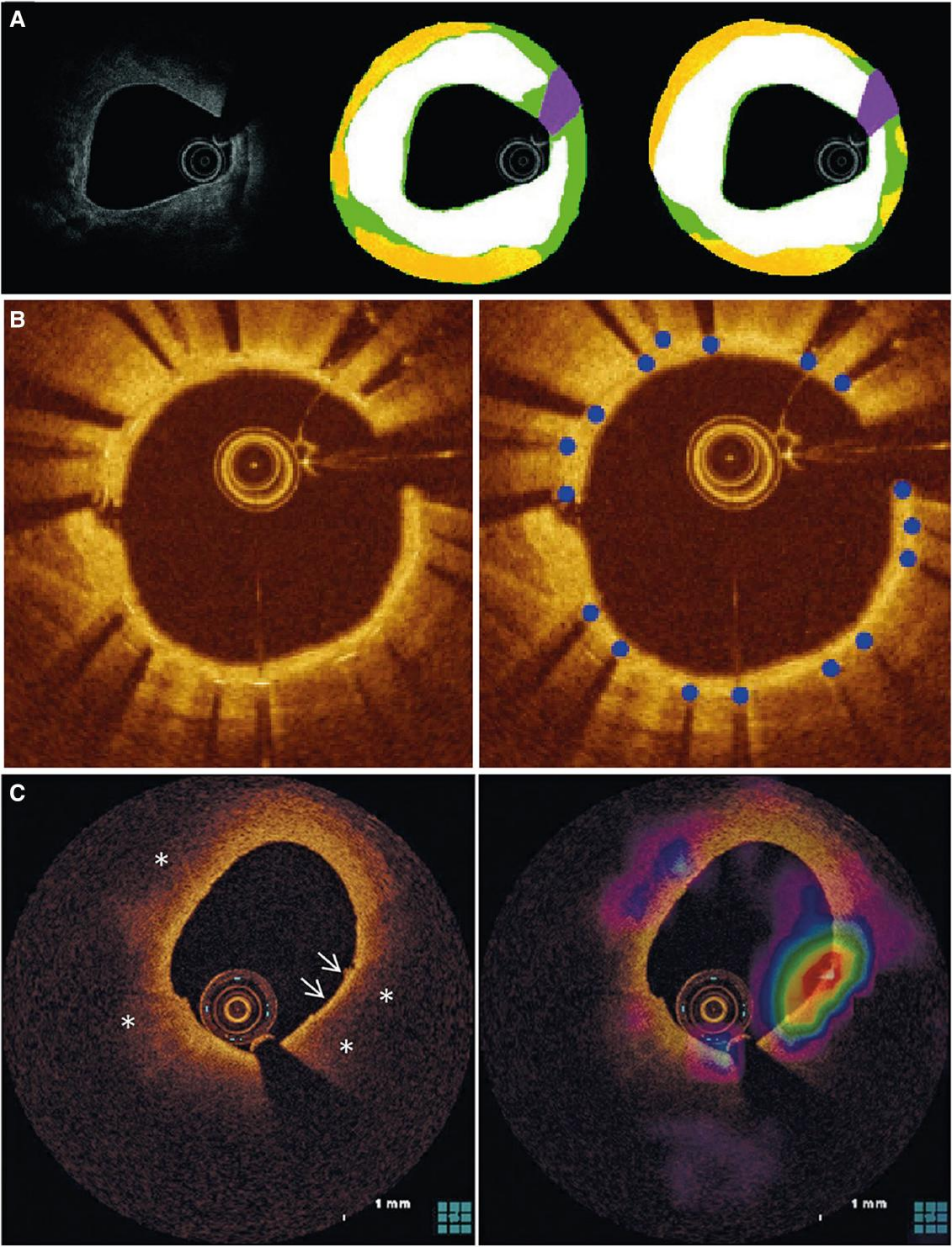
Examples of artificial intelligence algorithms for different tasks. (*A*) Multiclass semantic segmentation model proposed by Chu *et al*. The raw optical coherence tomography image is presented in the left image, the reference standard in the middle image, and the model prediction in the right image. White represents calcium, green represents intima/fibrous, yellow represents lipid, and purple represents guidewire artefact. Used with permission of EuroIntervention, from Artificial intelligence and optical coherence tomography for the automatic characterization of human atherosclerotic plaques, Chu, 17, 1, 2021; permission conveyed through Copyright Clearance Center, Inc.^28^ (*B*) Detection model for the localization of metal stent struts using centroids proposed by Yang *et al*. Adapted with permission from Yang *et al*.^29^ © Optica Publishing Group. (*C*) Framewise classification for the presence vs. absence of thin-cap fibroatheroma proposed by Min *et al*. The right panel represents the gradient-weighted class activation mapping (Grad-CAM) with red colours indicating the high attended regions to predict thin-cap fibroatheroma. Used with permission of EuroIntervention, from Detection of optical coherence tomography defined thin-cap fibroatheroma in the coronary artery using deep learning, Min, 16, 5, 2020; permission conveyed through Copyright Clearance Center, Inc.^30^

First, OCT is employed to determine stent sizes, which requires accurate quantification of lumen and vessel dimensions. We identified fourteen AI algorithms for lumen segmentation which demonstrated excellent performance. Mean differences between predicted and reference lumen areas were generally <0.50 mm^2^. Although vessel-based sizing is preferred over lumen-based stent sizing, only two studies reported media segmentation. In coronary OCT, assessing vessel sizes can be challenging in the presence of lipidic or thick calcified plaques, due to the light-attenuating nature of these tissues. Chu *et al*.^28^ extrapolated the visible parts of the internal elastic lamina to approximate the vessel area in their nine-class segmentation model based on 11673 frames with and without plaque (*Figure 6A*) and achieved a Dice score of 0.989 for vessel area segmentation. This model was also externally validated, showing a good correlation (ICC 0.81) with co-registered intravascular ultrasound-derived plaque burden.^31^

Second, OCT is recommended post-PCI to detect signs of suboptimal stent implantation, such as underexpansion, malapposition, and edge dissections. Overall, identified AI models targeting stent classification, detection, or segmentation showed excellent performance. For example, Yang *et al*.^29^ introduced a deep learning-based segmentation model to identify multiple stent layers (*Figure 6B*). The model performed well in identifying stent struts with both thin and thick tissue coverage (PPV 93.2 ± 0.9% and 85.6 ± 1.9% on a strut level, respectively), without excluding any frames. Overall, correlations between manual and automated stent area assessment were high (>0.90) in all studies. Additionally, the highest absolute mean difference between prediction and the reference standard was 0.36 mm^2^ for stent area and the mean differences for malapposition distance was <20 µm in both studies reporting this measure,^32,33^ suggesting that current models accurately enable detection of suboptimal stent placement. However, none of the algorithms addressed other clinically significant features such as tissue protrusion, edge dissections, or residual disease.

### Lesion preparation

Achieving optimal stent expansion is pivotal for long-term stent durability, but challenging in case of extensive calcification. Fujino *et al*.^34^ previously proposed an OCT-based risk score to identify lesions requiring specialized preparation to achieve sufficient stent expansion (i.e. calcified lesions with the combined presence of a lipid arc >180^°^, thickness >500 µm, and length >5 mm). While studies showed lower performances in evaluating calcified plaque compared with lumen and stent assessment, efforts to quantify calcification continue. Lee *et al*.^35^ performed multiple studies and achieved reasonable mean differences in calcium arc (13.2 ± 16.8°) and length (10.8 ± 20.1 mm) compared with core lab analysis, and excellent reproducibility in a cadaveric *ex vivo* dataset.^36^

Incorporating volumetric measures, such as total calcification volume, or differentiating types of calcification, could theoretically improve predictions.^37^ However, on-site evaluation of calcification volume is infeasible without computer assistance. Among 21 studies addressing calcified plaque segmentation, only three studies compared predicted calcium area or volume to a reference standard, all with high agreement. Notably, only one study employed AI to predict acute stent expansion from pre-interventional OCT frames and achieved a good correlation (0.94) between the predicted and actual minimum lumen area after PCI. The model had a sensitivity of 87% and a PPV of 84% for the prediction of proper stent expansion (defined as >80%).^38^ Intriguingly, this model outperformed the calcium score proposed by Fujino *et al*.^34^ However, the latter showed poor discrimination on the testing dataset (AUC 0.521), underscoring the need for external validation of both this score and the AI model.

### Lipidic and high-risk plaque assessment

Compared with calcified plaques, manually detecting lipidic plaques on OCT frames is more challenging and exhibits substantial interobserver variability,^39^ and an even larger variability compared to histology.^40^ We identified several classification and segmentation models for automated lipid assessment. While these models show reasonable performance for framewise identification of lipidic plaques, moderate pixel-wise performances were observed, likely due to difficulties in predicting the abluminal lipid border caused by its light-attenuating nature. For instance, the Dice score for lipidic plaques in the study by Chu *et al*.^28^ for stable coronary lesions was 0.772 (*Figure 6A*). It is important to note that most models were evaluated against expert image analysis, with only a few studies using histology as a reference standard, calling for more extensive validation against histology.

Quantification of lipidic plaques can be particularly relevant for the identification of high-risk plaques, which are lipidic plaques prone to rupture and are characterized by a thin fibrous cap (variably defined as ≤55–75 µm), large lipid arc (variably defined as ≥90–180˚), small lumen area (variably defined as ≤3.0–4.0 mm^2^), and in some studies the presence of macrophages.^1^ Numerous prospective observational studies have demonstrated that high-risk plaques provide important prognostic information,^7–10^ and preventive treatment of such plaques is under active investigation. Intensive lipid lowering or anti-inflammatory therapy can indeed help stabilize high-risk plaques^41^ and preventive focal treatment has been proposed to seal high-risk plaques.^42^ Both strategies require accurate identification of high-risk plaques, that should also be readily available when pursuing focal treatment. We identified six studies targeting high-risk plaque classification, with a median accuracy and sensitivity >90%. In contrast, only modest PPV has been reported. For example, the study by Min *et al*.^30^ (*Figure 6C*) achieved a high framewise classification accuracy for thin-cap fibroatheroma of 90%, but a PPV of only 51% in the held-out test set and 28% in a small external test set. Furthermore, the relatively high false positive rate at the vessel-level of 31% would be unacceptable in clinical practice, if used to guide preventive focal treatment of high-risk plaques. Besides classification, preliminary attempts on high-risk plaque localization using detection models exhibit promising results.^43–46^ However, these studies used relatively small datasets (up to 2500 frames), emphasizing the need for validation in larger studies, and none compared the performance to histology.

Whether AI-assisted high-risk plaque identification can contribute to prognostication remains largely unclear. To date, three studies showed an association between AI-identified high-risk plaques and adverse clinical outcomes, with a PPV ranging between 5.2–14.0%, which seems slightly lower than manual core lab analyses.^7^ For example, Biccirè *et al*.^10^ found a substantially lower PPV for AI compared with the original core lab analysis (5.2% vs. 19.4%) on the CLIMA dataset. As such, there is insufficient evidence to support AI-driven high-risk plaque detection in its current state, highlighting the need for further research.

### Culprit lesion identification

In addition to analysing plaque composition, OCT could help identify the culprit lesion when it is ambiguous, such as in myocardial infarction without obstructive coronary artery disease, as well as elucidate mechanisms like plaque rupture, erosion, coronary dissection, and vasospasm, which may not always be visible on coronary angiograms. However, AI training for these models faces challenges due to the rarity of such structures, including thrombus, intimal ruptures, or dissections. In a study by Park *et al*., a transformer model demonstrated high performances for diagnosing plaque erosion, achieving a frame-level AUC of 0.94 and a lesion-level AUC of 0.91 in the external test set. Notably, the model was validated in a reader study to experienced and inexperienced physicians, outperforming the latter group in identifying plaque erosion. Further research is needed to establish the specific benefits of AI in identifying culprit lesions.

### Optical coherence tomography-derived fractional flow reserve estimation

The integration of computational flow indices could further enhance the utility of OCT, omitting the concurrent need for a pressure wire. Preliminary attempts were performed to employ AI to predict pressure wire-derived FFR from OCT images in small datasets, resulting in a sensitivity and PPV of 72.9–100% and 77.8–92.1%. These performances, which seem below those obtained using computational fluid dynamics models,^47,48^ suggest a limited applicability of currently proposed AI models for OCT-derived FFR for determining the haemodynamic significance of coronary lesions.

### Potential sources of bias

The potential of introducing systematic bias is a critical consideration throughout the process of development and evaluation of AI models. The bias signalling questions revealed a lack of reporting with regard to numerous aspects. Therefore, we advocate for the adherence to AI-specific reporting guidelines.^49^ Additionally, data leakage could not be ruled out in 49% of the articles in which the data were not randomized on a patient- or pullback-level, posing a risk of a substantial overestimation of model performance. In this context, validating models on external datasets is crucial before clinical adoption, a step only undertaken in 12% of the reviewed articles. Importantly, a positive answer to any of the signalling questions does not automatically rule out bias. For instance, in the majority of articles detailing the management of suboptimal frame quality, frames with significant artefacts were manually excluded, potentially limiting the generalizability of such algorithms to routine clinical OCT pullbacks. A potential solution is adopting a parallel classification model to identify frames with suboptimal quality, as proposed by Park *et al*.^50^

### Future perspectives

Overall, the identified algorithms demonstrate the feasibility of AI-aided interpretation of intracoronary OCT frames. However, multiple barriers need to be addressed to ensure clinical adoption. First, future efforts should account for potential sources of bias to ensure trustworthiness and generalizability. Furthermore, evaluating model performances and assessing clinical significance requires comparison with both inter- and intra-observer variability among expert physicians. The importance of histology-based OCT studies is underscored by these variabilities and strengthened by our findings, which show that pixel-wise metrics, as well as PPV, appeared lower in histology-based evaluation when compared with studies relying solely on manual OCT image interpretation. Consequently, incorporating histology as a reference standard could further improve the accuracy of AI-based OCT analysis, which has thus far only been implemented in four studies. Second, inference times ranged from seconds to minutes for both classification and segmentation tasks. Balancing model performance with fast real-time decision-making poses a challenge for clinical implementation. Third, the integration of user-friendly software packages would enhance the daily use of autonomous AI models. Despite reviewing nearly 100 articles, only five software packages were identified.^28,33,51–54^ Among these, only one CE-certified software package (Ultreon) is currently available that allows automated assessment of the lumen, media, stents, and calcium.^54^ Integration of this software package in a standardized workflow (MLD-MAX)^55^ has been shown to streamline image interpretation and a modest yet significant reduction in analysis time compared with the former, non-AI-aided, software package.^54^ Overall, unlocking the full potential of AI algorithms for intracoronary OCT will require improved infrastructure, extensive validation, regulatory frameworks, and sustainable business models—a stepwise process that has been outlined in recent work.^16,56^

Furthermore, large multicentre evaluations are generally lacking, and none of the models have been tested in a randomized clinical trial. This is particularly important for validating AI-guided treatment strategies of high-risk plaques or guidance of revascularization based on AI-derived flow indices, but could also be valuable for AI-aided periprocedural stent guidance. In the observational LightLab initiative, the AI-assisted MLD-MAX workflow for periprocedural guidance improved acute stent-related outcomes compared with variable workflows, with reduced resource usage and without prolonging procedural duration.^57^ This real-world impact of this AI-assisted workflow will be further evaluated in the prospective observational ILUMIEN-V AERO study, in which an all-comer population of 2000 patients will be included.^58^ Last, enhancing code and data availability could improve the interpretation of model performances, although availability is frequently restricted by ethical, legal, and/or financial considerations.

## Conclusions

In this systematic review focusing on the use of AI for the analysis of intracoronary OCT images, we observed a notable transition from traditional machine learning models to the development of deep learning-based methods. These advanced models primarily concentrated on tasks related to the analysis of the intracoronary lumen, atherosclerotic plaques and their composition, and the assessment of stents. While evaluation results for the coronary lumen and stent struts were excellent, AI-based assessment of atherosclerotic plaques remains challenging. Despite these advancements, important considerations related to potential bias are present and must be addressed in future research, to ensure the overall trustworthiness of developed AI models and to pave the way towards their clinical adoption.

## Lead author biography


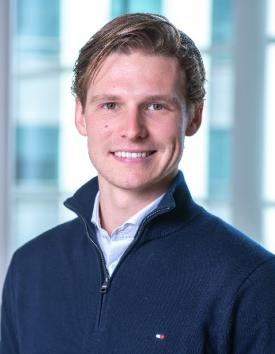


Ruben van der Waerden is a PhD student at the Department of Cardiology, Radboud University Medical Center (Nijmegen, The Netherlands), and the qurAI group (Amsterdam, The Netherlands), under the supervision of Prof. Niels van Royen and Prof. Ivana Išgum, respectively. His research, embedded within the CARA Lab, focuses on developing AI-driven techniques for analysing intracoronary OCT images. He specializes in vessel wall segmentation and plaque quantification, aiming to enhance diagnostic accuracy.

## Supplementary Material

ztaf005_Supplementary_Data

## Data Availability

The data underlying this systematic review are derived from publicly available sources. All articles analysed are available in Embase, Pubmed, Scopus, and Web of Science libraries and can be accessed through the references listed in the Supplementary File.
