# Supplementary material for: Artificial intelligence for the analysis of intracoronary optical coherence tomography images: a systematic review
Source: Eur Heart J Digit Health. 2025 Jan 28;6(2):270–84. doi: 10.1093/ehjdh/ztaf005 (PMC11914731; doi:10.1093/ehjdh/ztaf005)
Supplement: ztaf005_Supplementary_Data [file ztaf005_supplementary_data.docx]

Supplementary appendix

**Content**

[**Supplementary File 1 Protocol form** 3](#_Toc182326945)

[**Supplementary Table 1 Search queries** 6](#_Toc182326946)

[**Supplementary Table 2 Study characteristics** 7](#_Toc182326947)

[**Supplementary Table 3 Patient characteristics** 10](#_Toc182326948)

[**Supplementary Table 4 Algorithm characteristics** 12](#_Toc182326949)

[**Supplementary Table 5 Model performances for lumen** 18](#_Toc182326950)

[**Supplementary Table 6 Model performances for intima** 18](#_Toc182326951)

[**Supplementary Table 7 Model performances for media** 19](#_Toc182326952)

[**Supplementary Table 8 Model performances for sidebranch** 19](#_Toc182326953)

[**Supplementary Table 9 Model performances for lipid plaques** 20](#_Toc182326954)

[**Supplementary Table 10 Model performances for high-risk plaques** 21](#_Toc182326955)

[**Supplementary Table 11 Model performances for calcified plaques** 22](#_Toc182326956)

[**Supplementary Table 12 Model performances for fibrous plaques** 23](#_Toc182326957)

[**Supplementary Table 13 Model performances for plaque rupture** 24](#_Toc182326958)

[**Supplementary Table 14 Model performances for plaque erosion** 24](#_Toc182326959)

[**Supplementary Table 15 Model performances for layered plaques** 24](#_Toc182326960)

[**Supplementary Table 16 Model performances for undefined plaques** 25](#_Toc182326961)

[**Supplementary Table 17 Model performances for thrombus** 25](#_Toc182326962)

[**Supplementary Table 18 Model performances for macrophage accumulation** 25](#_Toc182326963)

[**Supplementary Table 19 Model performances for cholesterol clefts** 26](#_Toc182326964)

[**Supplementary Table 20 Model performances for microvessels** 26](#_Toc182326965)

[**Supplementary Table 21 Model performances for stents** 27](#_Toc182326966)

[**Supplementary Table 22 Model performances for fractional flow reserve** 28](#_Toc182326967)

[**Supplementary Table 23 Model performances for artifacts** 28](#_Toc182326968)

[**Supplementary Table 24 Model performances for other objectives** 28](#_Toc182326969)

[**Supplementary Table 25 Model performance for quantification** 29](#_Toc182326970)

[**Supplementary Table 26 Model performances for stent-related, plaque-related or clinical outcome** 31](#_Toc182326971)

[**Supplementary Table 27 Bias signaling questions** 32](#_Toc182326972)

[**References** 33](#_Toc182326973)

# **Supplementary File 1 Protocol form**

| Name investigator |  |
| --- | --- |
| Date (dd/mmm/yyyy) |  |

**ARTICLE INFORMATION**

| First author and year of publication |  |
| --- | --- |
| Title of the article |  |
| Journal |  |

**STUDY CHARACTERISTICS**

| Study period |  |
| --- | --- |
| Inclusion criteria |  |
| Exclusion criteria |  |
| Population analyzed in the article |  |
| Name of the database |  |
| Age of participants (mean ± standard deviation) |  |
| Female (n [%]) |  |
| Clinical presentation of participants (n [%]) |  |
| Diabetes mellitus (n [%]) |  |
| Hypertension (n [%]) |  |
| Previous myocardial infarction (n [%]) |  |

**DATASET CHARACTERISTICS**

| Dataset is organized between training and test set | - Yes - No |
| --- | --- |
| Total amount of pullbacks (n) |  |
| Total amount of frames (n) |  |
| Training set |  |
| Total amount of patients |  |
| Total amount of pullbacks |  |
| Total amount of frames |  |
| Validation set |  |
| Total amount of patients |  |
| Total amount of pullbacks |  |
| Total amount of frames |  |
| Test set |  |
| Total amount of patients |  |
| Total amount of pullbacks |  |
| Total amount of frames |  |
| External validation (if yes, [patients; pullbacks; frames]) | - No - Yes (…Patients, … Pullbacks, …. Frames) |

**ALGORITHM CHARACTERISTICS**

| Image coordinate system (Cartesian/Polar) | - Cartesian - Polar - Unknown |
| --- | --- |
| Model type |  |
| Transfer learning (if yes, type of model and weights initiation) | - No - Yes (Model type and weights initiation: …..) |
| Clinical setting (*ex vivo/in vivo*) | - In vivo - Ex vivo |
| Input shape | - 2D - 3D - Pseudo-3D (± … Frames) - Other, … |
| Output shape | - 2D - 3D - Pseudo-3D (± … Frames) - Other, … |
| Input color (RGB, Grey, not reported) | - RGB - Grey - Not reported |
| Data augmentation | - Yes, … - No |
| Output shape |  |
| Evaluated shape | - Pixelwise - Framewise - A-line - Other, … |
| Epochs |  |
| Optimizer |  |
| Loss function |  |
| Cross validation | … Folds |
| Annotation labels |  |
| AI tasks |  |
| Ground truth | - One expert - Multiple experts - Unknown |
| Analysis time - Frame | … Seconds |
| Analysis time – Pullback | … Seconds |
| Data availability | - Yes - Yes, upon request - No - Other,…. |
| Code availability | - Yes - Yes, upon request - No - Other,…. |

**PERFORMANCE RESULTS**

| **Class** |  |  |  |  |
| --- | --- | --- | --- | --- |
| **Task** |  |  |  |  |
| **Reference** |  |  |  |  |
| Accuracy |  |  |  |  |
| Sensitivity |  |  |  |  |
| Specificity |  |  |  |  |
| PPV |  |  |  |  |
| Dice score |  |  |  |  |
| NPV |  |  |  |  |
| F1 |  |  |  |  |
| AUC-ROC |  |  |  |  |
| Jaccard |  |  |  |  |

# **Supplementary Table 1 Search queries**

| **Database** | **Search terms** |
| --- | --- |
| Embase | ((('Optical coherence tomography' OR OCT OR OFDI OR 'optical frequency domain imaging' OR ivoct) AND ('Coronary vessel*' OR Coronary OR intravascular OR vascular OR vessel* OR intracoron*)) AND ('artificial intelligence' OR 'computational intelligence' OR 'machine intelligence' OR AI OR 'machine learn*' OR 'deep learn*' OR 'sentiment analys*' OR 'natural language proces*' OR 'neural network*' OR Robotics OR 'computer reason*' OR Artificial OR 'fuzzy logic' OR 'computer vision system*' OR automat* OR “Algorithm” OR “Radiomics”)).ti,ab. |
| Pubmed | ("tomography, optical coherence"[MeSH Terms] OR "OCT"[Title/Abstract] OR "optical coherence tomography"[Title/Abstract] OR "OFDI"[Title/Abstract] OR "optical frequency domain imaging"[Title/Abstract] OR "ivoct"[Title/Abstract]) AND ("coronary vessels"[MeSH Terms] OR "Coronary"[Title/Abstract] OR "intravascular"[Title/Abstract] OR "vascular"[Title/Abstract] OR "vessel*"[Title/Abstract] OR "intracoron*"[Title/Abstract]) AND ("artificial intelligence"[MeSH Terms] OR "artificial intelligence"[Title/Abstract] OR "computational intelligence"[Title/Abstract] OR "machine intelligence"[Title/Abstract] OR "AI"[Title/Abstract] OR "machine learn*"[Title/Abstract] OR "deep learn*"[Title/Abstract] OR "sentiment analys*"[Title/Abstract] OR "natural language proces*"[Title/Abstract] OR "neural network*"[Title/Abstract] OR "Robotics"[Title/Abstract] OR "machine intelligence"[Title/Abstract] OR "computer reason*"[Title/Abstract] OR "Artificial"[Title/Abstract] OR "computer reason*"[Title/Abstract] OR "fuzzy logic"[Title/Abstract] OR "computer vision system*"[Title/Abstract] OR automat* [Title/Abstract] OR “Algorithm” [Title/Abstract] OR “Radiomics” [Title/Abstract]) |
| Scopus | ( TITLE-ABS-KEY ( "Optical coherence tomography" OR "OCT" OR “OFDI" OR "optical frequency domain imaging" OR "ivoct")) AND (TITLE-ABS-KEY ( "Coronary" OR "intravascular" OR "vascular" OR "vessel" OR "intracoron*" )) AND (TITLE-ABS-KEY ( "artificial intelligence" OR "computational intelligence" OR "machine intelligence" OR "AI" OR "machine learn*" OR "deep learn*" OR "sentiment analys*" OR "natural language proces*" OR "neural network*" OR "Robotics" OR "machine intelligence" OR "computer reason*" OR "Artificial" OR "fuzzy logic" OR "computer vision system*" OR “automat*” OR “Algorithm” OR “Radiomics”)) |
| Web of Science | (TI=((Optical coherence tomography OR OCT OR OFDI OR optical frequency domain imaging OR ivoct) AND (Coronary OR intravascular OR vascular OR vessel* OR intracoron* ) AND ("artificial intelligence" OR "computational intelligence" OR "machine intelligence" OR "AI" OR "machine learn*" OR "deep learn*" OR "sentiment analys*" OR "natural language proces*" OR "neural network*" OR "Robotics" OR "machine intelligence" OR "computer reason*" OR "Artificial" OR "computer reason*" OR "fuzzy logic" OR "computer vision system*" OR “automat*” OR “Algorithm” OR “Radiomics”))) OR (AB=((Optical coherence tomography OR OCT OR OFDI OR optical frequency domain imaging OR ivoct) AND (Coronary vessel* OR Coronary OR intravascular OR vascular OR vessel* OR intracoron*) AND ("artificial intelligence" OR "computational intelligence" OR "machine intelligence" OR "AI" OR "machine learn*" OR "deep learn*" OR "sentiment analys*" OR "natural language proces*" OR "neural network*" OR "Robotics" OR "machine intelligence" OR "computer reason*" OR "Artificial" OR "computer reason*" OR "fuzzy logic" OR "computer vision system*" OR “automat*”))) OR (TS=((Optical coherence tomography OR OCT OR OFDI OR optical frequency domain imaging OR ivoct) AND (Coronary OR intravascular OR vascular OR vessel* OR intracoron*) AND ("artificial intelligence" OR "computational intelligence" OR "machine intelligence" OR "AI" OR "machine learn*" OR "deep learn*" OR "sentiment analys*" OR "natural language proces*" OR "neural network*" OR "Robotics" OR "machine intelligence" OR "computer reason*" OR "Artificial" OR "computer reason*" OR "fuzzy logic" OR "computer vision system*" OR “automat*” OR “Algorithm” OR “Radiomics”))) |

Overview of search inputs used for the different databases. The latest search was performed on 31-12-2023.

# **Supplementary Table 2 Study characteristics**

| No. | First author | patients (n) | Pullbacks (n) | Frames (n) | SETTING | Inclusion criteria | Exclusion criteria |
| --- | --- | --- | --- | --- | --- | --- | --- |
| 1 | Lu, 2012 | - | 12 | 508 | In vivo | Post-interventional | - |
| 2 | Ughi, 2013 | 49 | 49 | 64 | In vivo | - | - |
| 3 | Athanasiou, 2014 | 22 | 27 | 200 | In vivo | - | Malapposed struts, increased thrombus burden, residual blood, motion artifacts |
| 4 | Macedo, 2015 | 9 | 9 | 1,460 | In vivo | Pre-interventional | Frames of the trunk blood vessel |
| 5 | Zhao, 2015 | 72 | 103 | 8,332 | In vivo | - | - |
| 6 | He, 2016 | 20 | 33 | 102 | In vivo | Frames with lipid-rich plaque | - |
| 7 | Nam, 2016 | 18 | 20 | 800 | In vivo | - | - |
| 8 | Abdolmanafi, 2017 | 26 | 26 | 4,800 | In vivo | Kawasaki disease | - |
| 9 | Essa, 2017 | - | 13 | 2,303 | In vivo | - | - |
| 10 | Shalev, 2017 | 35 | 35 | 287 | Both | - | - |
| 11 | Yong, 2017 | 28 | 64 | 19,027 | In vivo | CAD | Images of guiding catheter |
| 12 | Zahnd, 2017 | 40 | 40 | 400 | In vivo | CAD | Stent and bifurcation |
| 13 | Amrute, 2018 | 15 | - | 1,140 | In vivo | - | Expert disagreement, excess blood in lumen and around struts, presence of bifurcations obscuring the luminal outline, presence of multiple scaffolds on top of one another |
| 14 | Athanasiou, 2018 | 11 | 11 | 613 | In vivo | - | Artifacts (saturation, foldover and pixel proximity) |
| 15 | Cao, 2018 | - | 15 | 4,065 | In vivo | BVS | - |
| 16 | Chen, 2018 | 50 | 100 | 43,873 | In vivo | Heart transplant recipients  Age ≥18 years | Renal insufficiency ≥ stage IV (eGFR <30 ml/min), unfavorable post-transplant clinical conduction such as severe rejection or nosocomial sepsis with prolonged antibiotic treatment during the first month, ongoing need for circulatory support using a ventricular assist device, acute allograft failure |
| 17 | Huang, 2018 | 11 | - | 28 | In vivo | - | Artifacts and interferences on images |
| 18 | Kolluru, 2018 | 48 | 48 | 4,469 | In vivo | Pre-interventional | - |
| 19 | Abdolmanafi, 2019 | 45 | 45 | 5,040 | In vivo | Kawasaki disease | - |
| 20 | Gessert, 2019 | 49 | - | 4,000 | In vivo | - | Stents |
| 21 | Gharaibeh, 2019 | - | 34 | 2,640 | In vivo | - | Stents |
| 22 | Lee, 2019 | 55 | 57 | 4,892 | In vivo | - | - |
| 23 | Li, 2019 | - | - | 1,000 | In vivo | - | - |
| 24 | Liu, 2019 | - | - | 2,300 | In vivo | - | - |
| 25 | Lu, 2019 | - | 80 | 7,125 | In vivo | - | - |
| 26 | Miyagawa, 2019 | 9 | 9 | 1,361 | In vivo | - | - |
| 27 | Prabhu, 2019 | - | 49 *in vivo* 10 *ex vivo* | 6,556 *in vivo*  440 *ex vivo* | Both | - | - |
| 28 | Tang, 2019 | - | - | 800 | In vivo | - | - |
| 29 | Yan, 2019 | 47 | 47 | - | In vivo | - | - |
| 30 | Yang, 2019 | 9 | 20 | 1,700 | In vivo | - | - |
| 31 | Zhang, 2019 | - | - | - | In vivo | CAD | - |
| 32 | Zhang, 2019 | 5 | - | 77 | In vivo | Good image quality, lipid core | - |
| 33 | Cha, 2020 | 125 | 125 | - | In vivo | Typical angina, de novo intermediate stenosis (40-70%) in LAD proximal to mid-segment, lesion length <20 mm | Hypersensitivity to contrast agent, use of inotropic agents due to hemodynamic instability, severe ventricular dysfunction (LVEF <30%), creatinine ≥2.0mg/dL, life expectancy <12 months due to noncardiac comorbidity, severe heart valve disease.  Poor image quality (insufficient blood clearance, improper coverage of entire lesion) |
| 34 | Gao, 2020 | - | - | 2,018 OCT  2,018 IVUS | In vivo | - | - |
| 35 | He, 2020 | 24 | 24 | 5,144 | In vivo | - | - |
| 36 | Jiang, 2020 | - | 10 | 165 | In vivo | - | - |
| 37 | Lee, 2020 | 49 | - | 6,556 | In vivo | - | Poor image quality, stents |
| 38 | Lee, 2020 | 68 in vivo  4 ex vivo | 68 in vivo  8 ex vivo | 8,231 in vivo 4,320 ex vivo | Both | - | Poor image quality (luminal blood, unclear lumen, artifact or reverberation) |
| 39 | Lu, 2020 | - | 292 | - | In vivo | - | - |
| 40 | Min, 2020 | 602 | 602 | 45,400 | In vivo | Intermediate coronary lesion (30-85%) | Stented lesions, images of bifurcations, poor image quality |
| 41 | Wang, 2020 | - | - | 2,200 | In vivo | - | - |
| 42 | Wu, 2020 | - | 60 | 10,417 | In vivo | Metallic stent | Non-stents, poor image quality |
| 43 | Yang, 2020 | 54 | - | 14,207 | In vivo | Drug-eluting stent | - |
| 44 | Zhang, 2020 | 2 | - | 18 | In vivo | - | - |
| 45 | Abdolmanafi, 2021 | - | 41 | - | In vivo | - | - |
| 46 | Avital, 2021 | - | - | 540 | In vivo | - | Poor image quality |
| 47 | Balaji, 2021 | 22 | - | 12,011 | In vivo | - | - |
| 48 | Cheimariotis, 2021 | 33 | - | 183 | In vivo | - | - |
| 49 | Chu, 2021 | 391 | 509 | 11,673 | In vivo | Stable coronary lesions | Aorta-ostial lesions, bypass graft lesions, moderate or severe valvular heart disease, ACS <72h attributed to the imaged vessel and CTO in any other vessel.  Frames with plaque rupture, thrombus, stent struts, or insufficient quality. |
| 50 | Guo, 2021 | 1 | 2 | 45 | In vivo | CCS | ACS, severe calcified lesion, CTO, or chronic kidney disease (creatinine >1.5 mg/dL) |
| 51 | Holmberg, 2021 | 58 | - | 62 ex vivo  222 in vivo | Both | - | Stents |
| 52 | Huang, 2021 | - | - | 1,286 | In vivo | - | - |
| 53 | Isidori, 2021 | 63 | - | - | In vivo | - | - |
| 54 | Kolluru, 2021 | - | 41 | 3,741 | In vivo | - | Stents |
| 55 | Lau, 2021 | 27 | 51 | 17,799 | In vivo | - | - |
| 56 | Shibutani, 2021 | 45 | 105 | 1,103 | Ex vivo | - | Poor image quality |
| 57 | Sun, 2021 | - | - | 2,500 | In vivo | - | - |
| 58 | Yang, 2021 | 41 | 56 | 25,203 | In vivo | Metallic stent | - |
| 59 | Yin, 2021 | 31 | - | 2,000 | In vivo | - | Stents, hard to identify plaque composition |
| 60 | Cao, 2022 | 5 | - | 1,415 | In vivo | - | - |
| 61 | Chen, 2022 | - | 100 calcifications  50 nodules | 4,254 calcifications  570 nodules | In vivo | - | Stent, uninterpretable OCT due to residual blood, artifact or unclear lumen images |
| 62 | Hatfaludi, 2022 | 80 | 102 | - | In vivo | Age ≥18 years, CCS with indication for diagnostic coronary angiography due to intermediate or high likelihood of obstructive coronary artery disease, ≥1 lesion (visually estimated diameter stenosis 40-80%), invasive FFR required | Significant arrhythmia (HR >120/min), suspected ACS, atrial fibrillation, systolic blood pressure <90 mmHg, contraindication to betablocker, nitroglycerin or adenosine, life expectancy <2 years due to non-cardiac cause, pathological aortic valve, rest state angina, MI in last 6 months, aorta-ostial lesions, frames with catheter or suboptimal image quality, unable to provide informed consent |
| 63 | Hong, 2022 | 604 | 915 vessels | - | In vivo | ACS, successful revascularization of culprit, OCT ≥1 untreated non-culprit lesions | Poor image quality, non-culprit vessel < 30mm, iatrogenic disease due to OCT, severe valvulopathy |
| 64 | Huang, 2022 | 15 | - | 64 | In vivo | - | Incomplete lumen imaged by OCT or IVUS, arc of acoustic shadowing precluding vessel delineation in IVUS >90°, arc of invisible lumen in either modality due to the presence of side branch and/or artifact is >90° |
| 65 | Lee, 2022 | 79 | - | 8,403 | In vivo | CCS with documented ischemia or ACS | Unprotected left main disease, CTO, baseline serum creatinine >2.0 mg/dL, life expectancy <18 months, unsuitability for OCT imaging |
| 66 | Lee, 2022 | 41 | 43 | 4,360 | In vivo | CCS with documented ischemia or ACS | Unprotected left main disease, CTO, baseline serum creatinine >2.0 mg/dL, life expectance <18 months, unsuitability for OCT imaging |
| 67 | Lee, 2022 | 90 | 180 | - | In vivo | CCS with documented ischemia or ACS, undergoing stent implantation | Unprotected left main disease, CTO, baseline serum creatinine >2.0 mg/dL, life expectance <18 months, unsuitability for OCT imaging |
| 68 | Li, 2022 | 45 | 45 | 13,844 | In vivo | - | - |
| 69 | Niioka, 2022 | 1,791 | - | 46,120 | In vivo | Non-culprit lesions with clinically overt CAD, or angiographically normal or minor stenosis (<25%) segments | Poor image quality, severe calcification |
| 70 | Olender, 2022 | 21 | - | - | In vivo | Stenotic CAD | - |
| 71 | Park, 2022 | 581 | 581 | 237,021 | In vivo | ACS | ACS caused by uncommon pathologies (i.e. calcified nodules, spontaneous coronary dissection, coronary spasm) |
| 72 | Rico-Jimenez, 2022 | 5 | 98 | 98 | Ex vivo | - | - |
| 73 | Shi, 2022 | - | - | 2,300 | In vivo | - | - |
| 74 | Sun, 2022 | 83 | 83 | 8,223 | In vivo | ACS with culprit plaque erosion | Stents |
| 75 | Wu, 2022 | 70 | 70 | 1,950 | In vivo | - | - |
| 76 | Araki, 2023 | 581 | 581 | 237,021 | In vivo | ACS, pre-interventional OCT of culprit lesion | ACS caused by uncommon pathologies (i.e. calcified nodules, spontaneous coronary dissection, coronary spasm), first and last two frames |
| 77 | Biccire, 2023 | 1,003 | 1,003 | - | In vivo | Untreated coronary plaque in proximal LAD, in the context of clinical CAG | - |
| 78 | Cha, 2023 | 130 | 356 | - | In vivo | Coronary CT angiography because of chest pain and a de novo lesion of intermediate stenosis (40-70%) in proximal to middle portions | MI with single vessel disease, hypersensitivity to contrast agent, use of inotropic agents owing to hemodynamic instability, severe ventricular dysfunction (LVEF <30%), creatinine ≥2.0 mg/dL, life expectancy <12 months owning to noncardiac comorbidity, severe heart valve disease, poor image quality, incomplete OCT coverage of the lesion |
| 79 | Cioffi, 2023 | - | - | - | In vivo | - | - |
| 80 | Gharaibeh, 2023 | 104 | - | - | In vivo | - | Ostial lesions, severe tortuosity, occluding thrombus, bypass graft stenosis, in-stent restenosis, CTO, non-calcified frames, frames following plaque modification |
| 81 | Han, 2023 | 46 | - | 5,781 | In vivo | - | Non-stents |
| 82 | Lee, 2023 | 34 | 34 | 2,723 | In vivo | Calcified lesion | Poor image quality (luminal blood, unclear lumen, artifact or reverberation), Side branch |
| 83 | Liu, 2023 | - | - | 2,300 | In vivo | 18-80 years old | Pre-interventional |
| 84 | Lv, 2023 | 10 | - | 114 baseline | In vivo | CCS | Frames without lipid core; ACS patients, severe calcified lesion, CTO, chronic kidney disease |
| 85 | Oikawa, 2023 | 44 | - | 2,349 | In vivo | - | - |
| 86 | Ren, 2023 | 7 | 7 | 490 | In vivo | - | - |
| 87 | Shi, 2023 | - | - | 2,300 | In vivo | - | - |
| 88 | Shi, 2023 | 5 | - | 51 | In vivo | - | - |
| 89 | Tang, 2023 | 14 | - | 2,388 | In vivo | - | - |
| 90 | Wang, 2023 | 15 | 15 | 5,624 | In vivo | Culprit vessel | - |
| 91 | Wu, 2023 | 17 IVUS 148 OCT | 45 IVUS 382 OCT | 54,105 IVUS  60,587 OCT | In vivo | - | - |

Overview of study characteristics in included articles.
*ACS acute coronary syndrome; BVS Bioresorbable vascular scaffold; CAD coronary artery disease; CCS chronic coronary syndrome; CT computed tomography; CTO chronic total occlusion; eGFR estimated glomerular filtration rate; FFR fractional flow reserve; IVUS intravascular ultrasound; LAD left anterior descending artery; LVEF left ventricular ejection fraction; MI myocardial infarction; OCT optical coherence tomography*

# **Supplementary Table 3 Patient characteristics**

|  |  |  |  |  | Clinical scenario | | | | | |  |  |  |  |
| --- | --- | --- | --- | --- | --- | --- | --- | --- | --- | --- | --- | --- | --- | --- |
| No. | FIRST AUTHOR | AGE (YEARS) | FEMALE |  | ACS | STEMI | NSTEMI | UAP | CCS | Other |  | DIABETES | HYPERTENSION | PREVIOUS MI |
| 1 | Lu | - | - |  | - | - | - | - | - | - |  | - | - | - |
| 2 | Ughi | - | - |  | - | - | - | - | - | - |  | - | - | - |
| 3 | Athanasiou | - | - |  | - | - | - | - | - | - |  | - | - | - |
| 4 | Macedo | 65.33 ± 9.45 | 2 (22%] |  | - | - | - | - | - | - |  | 5 (56%) | 8 (89%) | - |
| 5 | Zhao | - | - |  | - | - | - | - | - | - |  | - | - | - |
| 6 | He | - | - |  | - | - | - | - | - | - |  | - | - | - |
| 7 | Nam | - | - |  | - | - | - | - | - | - |  | - | - | - |
| 8 | Abdolmanafi | - | - |  | - | - | - | - | - | - |  | - | - | - |
| 9 | Essa | - | - |  | - | - | - | - | - | - |  | - | - | - |
| 10 | Shalev | - | - |  | - | - | - | - | - | - |  | - | - | - |
| 11 | Yong | 59.71 ± 9.61 | 21 (75%) |  | - | - | - | - | - | - |  | - | - | - |
| 12 | Zahnd | - | - |  | - | - | - | - | - | - |  | - | - | - |
| 13 | Amrute | - | - |  | - | - | - | - | - | - |  | - | - | - |
| 14 | Athanasiou | - | - |  | - | - | - | - | - | - |  | - | - | - |
| 15 | Cao | - | - |  | - | - | - | - | - | - |  | - | - | - |
| 16 | Chen | - | - |  | - | - | - | - | - | - |  | - | - | - |
| 17 | Huang | 61 – 73 | 4 (36.4%) |  | - | - | - | - | - | - |  | - | - | - |
| 18 | Kolluru | - | - |  | - | - | - | - | - | - |  | - | - | - |
| 19 | Abdolmanafi | - | - |  | - | - | - | - | - | - |  | - | - | - |
| 20 | Gessert | - | - |  | - | - | - | - | - | - |  | - | - | - |
| 21 | Gharaibeh | - | - |  | - | - | - | - | - | - |  | - | - | - |
| 22 | Lee | - | - |  | - | - | - | - | - | - |  | - | - | - |
| 23 | Li | - | - |  | - | - | - | - | - | - |  | - | - | - |
| 24 | Liu | - | - |  | - | - | - | - | - | - |  | - | - | - |
| 25 | Lu | - | - |  | - | - | - | - | - | - |  | - | - | - |
| 26 | Miyagawa | - | - |  | - | - | - | - | - | - |  | - | - | - |
| 27 | Prabhu | - | - |  | - | - | - | - | - | - |  | - | - | - |
| 28 | Tang | - | - |  | - | - | - | - | - | - |  | - | - | - |
| 29 | Yan | - | - |  | - | - | - | - | - | - |  | - | - | - |
| 30 | Yang | - | - |  | - | - | - | - | - | - |  | - | - | - |
| 31 | Zhang | - | - |  | - | - | - | - | - | - |  | - | - | - |
| 32 | Zhang | - | - |  | - | - | - | - | - | - |  | - | - | - |
| 33 | Cha | 62.7 ± 9.1 | 31 (24.8%) |  | - | - | - | 40 (32%) | - | - |  | 39 (31.2%) | 77 (61.6%) | - |
| 34 | Gao | - | - |  | - | - | - | - | - | - |  | - | - | - |
| 35 | He | - | - |  | - | - | - | - | - | - |  | - | - | - |
| 36 | Jiang | - | - |  | - | - | - | - | - | - |  | - | - | - |
| 37 | Lee | - | - |  | - | - | - | - | - | - |  | - | - | - |
| 38 | Lee | - | - |  | - | - | - | - | - | - |  | - | - | - |
| 39 | Lu | - | - |  | - | - | - | - | - | - |  | - | - | - |
| 40 | Min | 65.5 ± 9.7 | 151 (25.1%) |  | 162 (27%) | - | - | - | - | - |  | 168 (28%) | 367 (61%) | - |
| 41 | Wang | - | - |  | - | - | - | - | - | - |  | - | - | - |
| 42 | Wu | - | - |  | - | - | - | - | - | - |  | - | - | - |
| 43 | Yang | - | - |  | - | - | - | - | - | - |  | - | - | - |
| 44 | Zhang | - | - |  | - | - | - | - | - | - |  | - | - | - |
| 45 | Abdolmanafi | - | - |  | - | - | - | - | - | - |  | - | - | - |
| 46 | Avital | - | - |  | - | - | - | - | - | - |  | - | - | - |
| 47 | Balaji | - | - |  | - | - | - | - | - | - |  | - | - | - |
| 48 | Cheimariotis | - | - |  | - | - | - | - | - | - |  | - | - | - |
| 49 | Chu | 66.4 ± 10.7 | 93 (23.8%) |  | - | - | 14 (3.6%) | 67 (17.1%) | 118 (30.2%) | 192 (49.1%) |  | 158 (40.4%) | 311 (79.5%) | 156 (39.9%) |
| 50 | Guo | 80 | 1 (100%) |  | - | - | - | - | 1 (100%) | - |  | - | - | - |
| 51 | Holmberg | 66.2 ± 14.7 | 16 (31.4%) |  | 6 (11.8%) | - | - | 4 (7.8%) | 31 (60.8%) | 10 (19.6%) |  | 25 (49%) | 43 (84.3%) | - |
| 52 | Huang | - | - |  | - | - | - | - | - | - |  | - | - | - |
| 53 | Isidori | C-ACS 70 (64-82) NC-ACS 59 (56-63) CCS 62 (52-66) | 9 (22.5%) |  | - | 13 (32.5%) | 11 (27.5%) | 4 (10%) | 12 (30%) | - |  | 10 (25%) | 34 (85%) | 6 (15%) |
| 54 | Kolluru | - | - |  | - | - | - | - | - | - |  | - | - | - |
| 55 | Lau | - | - |  | - | - | - | - | - | - |  | - | - | - |
| 56 | Shibutani | - | - |  | - | - | - | - | - | - |  | - | - | - |
| 57 | Sun | - | - |  | - | - | - | - | - | - |  | - | - | - |
| 58 | Yang | - | - |  | - | - | - | - | - | - |  | - | - | - |
| 59 | Yin | - | - |  | - | - | - | - | - | - |  | - | - | - |
| 60 | Cao | - | - |  | - | - | - | - | - | - |  | - | - | - |
| 61 | Chen | - | - |  | - | - | - | - | - | - |  | - | - | - |
| 62 | Hatfaludi | 60.5 ± 11.2 | 14 (18%) |  | - | - | - | - | 80 (100%) | - |  | 27 (33.75%) | 60 (75%) | 46 (45%) |
| 63 | Hong | 57.2 ± 11.2 | 145 (24%) |  | - | 406 (67.7%) | 131 (21.7%) | 64 (10.6%) | - | - |  | 106 (17.5%) | 273 (45.2%) | 41 (6.8%) |
| 64 | Huang | 59.14 ± 7.16 | 6 (40.0%) |  | - | - | 1 (6.7%) | - | 12 (80%) | - |  | 0 (0.0%) | 8 (53.3%) | - |
| 65 | Lee | - | - |  | - | - | - | - | - | - |  | - | - | - |
| 66 | Lee | 63.7 ± 10.0 | 6 (14.6%) |  | 8 (19.5%) | 15 (36.6%) | 5 (12.2%) | 4 (9.8%) | 9 (22.0%) | - |  | 6 (14.6%) | 28 (68.3%) | 5 (12.2%) |
| 67 | Lee | 64.0 ± 10.0 | 18 (20%) |  | - | 23 (25.6%) | 10 (11.1%) | 29 (32.2%) | 28 (31.1%) | - |  | 15 (16.7%) | 55 (61.1%) | 9 (10%) |
| 68 | Li | - | - |  | - | - | - | - | - | - |  | - | - | - |
| 69 | Niioka | 68.0 ± 11.3 | 369 (25.4%) |  | 495 (34.1%) | - | - | - | 644 (44.4%) | 180 (12.4%) |  | 544 (37.5%) | 1,071 (73.9%) | 428 (29.5%) |
| 70 | Olender | - | - |  | - | - | - | - | - | - |  | - | - | - |
| 71 | Park | 61.9 ± 12.1 | 187 (21.4%) |  | - | 653 (74.8%) | 220 (25.2%) | - | - | - |  | 254 (29.1%) | 492 (56.4%) | 42 (4.8%) |
| 72 | Rico-Jimenez | - | - |  | - | - | - | - | - | - |  | - | - | - |
| 73 | Shi | - | - |  | - | - | - | - | - | - |  | - | - | - |
| 74 | Sun | - | - |  | 83 (100%) | - | - | - | - | - |  | - | - | - |
| 75 | Wu | - | - |  | - | - | - | - | - | - |  | - | - | - |
| 76 | Araki | 61.9 ± 12.1 | 187 (21.4%) |  | - | 653 (74.8%) | 220 (25.2%) | - | - | - |  | 254 (29.1%) | 492 (56.4%) | 42 (4.8%) |
| 77 | Biccire | 64.8 | 247 (24.6%) |  | 536 (54.4%) | - | - | - | 467 (46.6%) | - |  | 223 (22.3%) | 678 (67.9%) | - |
| 78 | Cha | 62.5 ± 8.8 | 33 (25.4%) |  | - | - | - | - | - | - |  | 41 (31.5%) | 79 (60.8%) | - |
| 79 | Cioffi | - | - |  | - | - | - | - | - | - |  | - | - | - |
| 80 | Gharaibeh | 67.1 ± 12.0 | 30 (28.8%) |  | - | 10 (9.6%)^a^ | 35 (33.7%)^b^ | - | 57 (54.8%) | 1 (1.0%) |  | 56 (53.8%) | 99 (95.2%) | 60 (57.7%) |
| 81 | Han | - | - |  | - | - | - | - | - | - |  | - | - | - |
| 82 | Lee | - | - |  | - | - | - | - | - | - |  | - | - | - |
| 83 | Liu | - | - |  | - | - | - | - | - | - |  | - | - | - |
| 84 | Lv | 70.4 | 4 (40%) |  | - | - | - | - | 10 (100%) | - |  | 6 (60%) | 7 (70%) | - |
| 85 | Oikawa | - | - |  | - | - | - | - | - | - |  | - | - | - |
| 86 | Ren | - | - |  | - | - | - | - | 7 (100%) | - |  | - | - | - |
| 87 | Shi | - | - |  | - | - | - | - | - | - |  | - | - | - |
| 88 | Shi | - | - |  | - | - | - | - | - | - |  | - | - | - |
| 89 | Tang | - | - |  | - | - | - | - | - | - |  | - | - | - |
| 90 | Wang | - | - |  | - | - | - | - | - | - |  | - | - | - |
| 91 | Wu | - | - |  | - | - | - | - | - | - |  | - | - | - |

Overview of patient characteristics in included articles.
*ACS acute coronary syndrome; C-ACS culprit acute coronary syndrome; CCS chronic coronary syndrome; MI myocardial infarction; NC-ACS non-culprit acute coronary syndrome; NSTEMI non-ST-segment elevation myocardial infarction; STEMI ST-segment elevation myocardial infarction; UAP unstable angina pectoris
^a^ Includes STEMI and cardiogenic shock.
^b^ Includes NSTEMI and UAP.*

# **Supplementary Table 4 Algorithm characteristics**

|  |  | Data distribution^a^ | | |  |  |  |  |  |  |  |  |  |  |  |
| --- | --- | --- | --- | --- | --- | --- | --- | --- | --- | --- | --- | --- | --- | --- | --- |
| No. |  | TRAINING | VALIDATION | TEST |  | CROSS-VALIDATION | EXTERNAL VALIDATION | ML/DL | IMAGE COORDINATES | INPUT | COLOR | TASK(S) | ANALYSIS TIME | Data available | Code available |
| 1 |  | 12 pullbacks  508 frames | - | - |  | Leave-one-stent-out | No | ML | Polar | 2D | Greyscale | Classification | - | No | No |
| 2 |  | 24 frames | - | 40 frames |  | - | No | ML | Polar | 2D | Greyscale | Segmentation | Frame ± 30 s | No | No |
| 3 |  | 60 frames | - | 140 frames |  | 10 fold | No | ML | Cartesian | 2D | Greyscale | Segmentation | - | No |  |
| 4 |  | 9 patients  9 pullbacks  1,460 frames | - | - |  | 10 fold | No | ML | Cartesian | 2D | - | Classification | Frame 16 s (15 s segmentation and 1 s classification) | No | No |
| 5 |  | 103 pullbacks  8,332 frames | - | - |  | Leave one-out | No | ML | Polar | 2D | - | Classification | Pullback 120 s | No | No |
| 6 |  | - | - | 20 patients  33 pullbacks  102 frames |  | - | No | ML | Cartesian | 2D | Greyscale | Segmentation | - | No | No |
| 7 |  | $\frac{7}{10}$ of 800 frames | $\frac{3}{20}$ of 800 frames | $\frac{3}{20}$ of 800 frames |  | - | No | DL | Polar | 2D | - | Classification | Frame 0.47 s | No | No |
| 8 |  | 10 patients  $\frac{1}{3}$ of 4,800 ROI | 9 patients  $\frac{1}{3}$ of 4,800 ROI | 9 patients  $\frac{1}{3}$ of 4,800 ROI |  | - | No | Both | Polar | 2D | RGB | Classification | - | No | No |
| 9 |  | - | - | - |  | - | No | ML | Polar | 2D | - | Classification | - | No | No |
| 10 |  | 35 pullbacks | - | - |  | Leave-one-pullback-out | 106 frames | ML | Polar | 2D | - | Segmentation | Pullback 0.366 s | No | No |
| 11 |  | 45 pullbacks  13,342 frames | - | 19 pullbacks  5,685 frames |  | - | No | DL | Polar | 2D | Greyscale | Segmentation | Frame 40.6 ms | No | No |
| 12 |  | 14 patients  140 frames | - | 26 patients  260 frames |  | - | No | ML | Polar | Pseudo 3D  (± 3 frames) | - | Classification | Frame 2 s | No | No |
| 13 |  | - | - | 15 patients  1,140 frames |  | - | No | ML | Polar | 2D | - | Segmentation | Frame 3 s | No | No |
| 14 |  | - | - | 11 patients  11 pullbacks  613 frames |  | - | No | ML | Longitudinal cross sectionals | 2D | - | Segmentation | Pullback 120 s | No | No |
| 15 |  | 4 pullbacks 1,500 stent struts | 4 pullbacks 2,000 stent struts | 7 pullbacks  1,897 frames |  | - | No | ML | Cartesian | 2D | - | Detection | Pullback 10.69 s (detection only) | No | No |
| 16 |  | 50 patients  100 pullbacks  43,873 frames | - | - |  | 5 fold | No | DL | Cartesian patches | 2D | - | Classification | Pullback < 1 minute | No | No |
| 17 |  | - | - | - |  | - | No | ML | Polar | 2D | Greyscale | Segmentation | - | No | No |
| 18 |  | 38 patients 38 pullbacks | 5 patients  5 pullbacks | 5 patients  5 pullbacks |  | 10 fold^b^ | No | DL | Polar | 2D | Greyscale | Classification | - | No | No |
| 19 |  | $\frac{1}{2}$ of 5,040 frames | $\frac{1}{4}$ of 5040 frames | $\frac{1}{4}$ of 5040 frames |  | Leave-one-out | No | Both | Polar | 2D | RGB | Classification Segmentation | - | No | No |
| 20 |  | 40 patients 3,258 frames | - | 9 patients  742 frames |  | 3 fold | No | DL | Both | 2D | - | Classification | - | No | No |
| 20 |  | 40 patients  3,258 frames | - | 9 patients  742 frames |  | 3 fold | No | DL | Cartesian | 2D | - | Classification | - | No | No |
| 21 |  | $\frac{4}{5}$ of 48 VOI | $\frac{1}{10}$ of 48 VOI | $\frac{1}{10}$ of 48 VOI |  | 10 fold^b^ | No | DL | Polar | 2D | - | Segmentation | - | No | No |
| 22 |  | $\frac{9}{10}$ of 80 VOI | $\frac{1}{10}$ of 80 VOI | 9 VOI without plaque |  | 10 fold^b^ | No | DL | Polar | 2D | Greyscale | Segmentation | - | No | No |
| 23 |  | 800 frames  8,000 augmented frames | - | 200 frames |  | - | No | DL | Cartesian | 2D | - | Segmentation | - | No | No |
| 24 |  | 2,000 frames  40,000 augmented frames | - | 300 frames |  | - | No | Both | Polar | 2D | Greyscale | Detection | - | No | No |
| 25 |  | 80 pullbacks | - | - |  | 8 fold | No | ML | Cartesian | 2D | - | Classification | - | No | No |
| 26 |  | 9 patients  9 pullbacks  1,361 frames  2,460 augmented frames | - | - |  | 10 fold | No | DL | Cartesian | 2D | RGB | Classification | - | No | No |
| 27 |  | *in vivo*:  41 patients 4,819 frames.  *ex vivo*:  10 patients 440 frames | - | *in vivo*:  8 patients  1,737 frames |  | 5 fold | No | ML | Polar | 2D | Greyscale | Classification | - | No | No |
| 28 |  | 400 frames  20,000 augmented frames | - | 400 frames |  | - | No | DL | - | 2D | RGB | Segmentation | - | No | No |
| 29 |  | 6 patients  6 volumes | - | 41 patients 41 pullbacks |  | Leave-one-patient-out | No | ML | Cartesian | 2D | - | Classification | - | No | No |
| 30 |  | 1,700 frames | - | 300 frames |  | 5 fold^b^ | No | ML | Cartesian | 2D | - | Classification | - | No | No |
| 31 |  | 13,000 augmented frames | - | 5,600 frames |  | Yes, unknown folds | No | DL | Cartesian patches | 2D | Greyscale | Segmentation | Frame  4.67 s for calcified/lipid  4.84 s for fibrotic | No | No |
| 32 |  | 70 images | - | 7 images |  | 11 fold | No | DL | Cartesian | 2D | - | Segmentation | - | No | No |
| 33 |  | 104 patients  104 pullbacks | - | 21 patients  21 pullbacks |  | 4 fold | No | ML | - | 2D | - | Regression | - | Yes^c^ | No |
| 34 |  | 2,018 OCT and 2,018 matched IVUS frames | - | - |  | 10 | No | DL | Cartesian | 2D | - | Segmentation | - | No | No |
| 35 |  | 18 patients  18 pullbacks  4,000 frames | 2 patients  2 pullbacks  344 frames | 4 patients  4 pullbacks  800 frames |  | 10 fold^b^ | No | DL | Polar | 3D  (majority voting of 3, 5 and 7 frames) | RGB | Classification | - | No | No |
| 36 |  | 100 frames  1,200 augmented frames | 33 frames | 32 frames |  | - | No | DL | Cartesian | 2D | - | Detection | - | Yes^c^ | No |
| 37 |  | 89 VOI  4,819 frames | - | 22 VOI  1,737 frames |  | 5 fold | No | Both | Polar | 2D | Greyscale | Classification | - | Yes^c^ | No |
| 38 |  | $\frac{9}{10}$ of 68 pullbacks | - | $\frac{1}{10}$ of 68 pullbacks |  | 5 fold^b^ | No | DL | Polar | 3D  (5 frames) | - | Classification | Frame 0.3 s | No | No |
| 38 |  | $\frac{9}{10}$ of 68 pullbacks (positive frames only) | - | $\frac{1}{10}$ of 68 pullbacks (positive frames only) |  | 5 fold^b^ | 8 pullbacks  4,320 frames | DL | Polar | 2D | - | Segmentation | Frame 0.3 s | No | No |
| 39 |  | NA | NA | NA |  | NA | NA | NA | NA | NA | NA | Software | - | Yes^c^ | No |
| 40 |  | 480 patients  480 pullbacks  35,678 frames | - | 122 patients  122 pullbacks  9,722 frames |  | 5 fold | 65 patients  65 frames | DL | Cartesian | 2D | - | Classification | Pullback 2.1 s | No | No |
| 41 |  | 2,000 frames | - | 200 frames |  | - | No | DL | Polar | 2D | Greyscale | Classification | - | No | No |
| 42 |  | $\frac{4}{5}$of 10,417 frames | $\frac{1}{5}$ of 10,417 frames | - |  | - | 170 pullbacks 21,363 frames | DL | Polar | Pseudo-3D  (± 7 frames) | Greyscale | Segmentation | Frame 0.02 s (detection and segmentation only) | No | No |
| 43 |  | 54 patients 14,207 frames | - | - |  | 10 fold | No | ML | Cartesian | 2D | - | Classification | Frame 5-10 s | No | No |
| 44 |  | 18 frames  1,512 augmented frames | - | - |  | 3 fold | No | DL | - | 2D | - | Segmentation | Frame 1-2 s | No | No |
| 45 |  | $\frac{9}{10}$ of frames | - | $\frac{1}{10}$ of frames |  | Leave-one-out | No | DL | - | 2D | - | Segmentation | Frame 0.3 s | No | No |
| 45 |  | $\frac{17}{20}$ of 2,052 frames | - | $\frac{3}{20}$ of 2052 frames |  | Leave-one-out | No | DL | - | 2D segment | - | Classification | - | No | No |
| 46 |  | 490 frames | - | 50 frames |  | - | No | DL | Cartesian | 2D | RGB | Segmentation | - | Yes^c^ | No |
| 47 |  | $\frac{7}{10}$ of pullbacks  8,502 frames | $\frac{1}{5}$ of pullbacks 2,499 frames | $\frac{1}{10}$ of pullbacks 1,010 frames |  | - | No | DL | Cartesian | 2D | - | Segmentation | Frame 4.4 ms | No | No |
| 48 |  | $\frac{5}{6}$ of 33 patients | - | $\frac{1}{6}$ of 33 patients |  | 5 fold^b^ | No | DL | Polar | 2D | RGB | Classification | - | Yes^c^ | No |
| 48 |  | $\frac{5}{6}$ of 33 patients | - | $\frac{1}{6}$ of 33 patients |  | 5 fold^b^ | No | DL | Polar | 2D | RGB | Classification | - | Yes^c^ | No |
| 49 |  | 10,517 frames | - | 1,156 frames |  | - | 30 patients  30 pullbacks  300 frames | DL | Cartesian | Pseudo 3D | Greyscale | Segmentation  Software | Pullback 21.4 s (IQR 18.6-25.0) Frame 0.07 ± 0.01 s | No | No |
| 50 |  | 45 frames | - | - |  | 5 fold | No | ML | - | 2D | - | Prognosis | - | Yes^c^ | No |
| 51 |  | 58 patients (51 in vivo, 7 ex vivo); 284 frames (222 in vivo, 62 ex vivo) |  |  |  | Leave-one-patient-out | 11 patients  3,284 frames | DL | Cartesian | 2D | RGB | Segmentation | Pullback 60 s Frame 0.2 s | Yes^c^ | No |
| 52 |  | 1,046 frames  10,460 augmented frames | - | 240 frames |  | No | No | DL | Cartesian | 2D | - | Segmentation | - | No | No |
| 53 |  | 210,000 samples | 90,000 samples | 40 patients; 40 pullbacks |  | No | No | DL | Polar patches | 2D | - | Classification | Pullback 10.08 minutes ± 15.4 s | No | No |
| 54 |  | $\frac{3}{4}$ of 60 VOI | - | $\frac{1}{4}$ of 60 VOI |  | 5 fold^b^ | No | DL | Polar | 2D | - | Segmentation | - | No | No |
| 55 |  | 41 pullbacks  13,890 frames | - | 10 pullbacks  3,909 frames |  | - | No | DL | Polar patches | Pseudo 3D  (± 3 frames) | - | Segmentation | Pullback 54 s | No | No |
| 56 |  | 875 images | 112 frames | 116 frames |  | - | No | DL | Cartesian | 2D | RGB | Segmentation | Frame 0.26 s | No | Yes |
| 57 |  | 2,000 frames | - | 500 frames |  | - | No | DL | Polar | 2D | - | Detection | - | No | No |
| 58 |  | 41 patients  56 pullbacks  25,203 frames | - | - |  | 3 fold | No | DL | Polar | 2D | - | Segmentation | Frame 59 s | Yes^c^ | No |
| 58 |  | 623 frames  1,869 augmented frames | - | 18 patients  23 pullbacks  9,659 frames |  | - | No | DL | Cartesian | 2D | - | Classification | - | Yes^c^ | No |
| 58 |  | 8,051 frames | - | 2,684 frames |  | - | No | DL | Cartesian | 2D | - | Segmentation | - | Yes^c^ | No |
| 59 |  | $\frac{5}{6}$ of frames | $\frac{1}{6}$ of frames | - |  | Yes, unknown folds | No | DL | Cartesian patches | 2D | RGB | Segmentation | Frame <3 s | Yes^c^ | No |
| 60 |  | 936 frames (520 inner contour, 416 outer contour)  11,412 augmented frames (6,240 inner contour, 4,992 outer contour) | - | 479 frames (375 inner contour, 104 outer contour) |  | - | No | DL | Cartesian | 2D | - | Segmentation | - | No | No |
| 61 |  | 3,900 frames | - | 354 frames |  | - | No | DL | Cartesian | 2D | - | Segmentation | Pullback 12 s Frame 0.03 s | No | No |
| 61 |  | 470 frames | - | 100 frames |  | - | No | DL | Cartesian | Pseudo 3D | - | Segmentation | - | No | No |
| 62 |  | 80 patients  102 pullbacks | - | - |  | Leave-one-out | No | DL | - | 2D | RGB | Classification | Pullback 25 ms | Yes^c^ | No |
| 63 |  | NA | NA | NA |  | NA | NA | NA | NA | NA | NA | Prognosis | Pullback <1 minute | No | No |
| 64 |  | NA | NA | NA |  | NA | Yes | NA | NA | NA | NA | External validation | - | No | No |
| 65 |  | $\frac{17}{20}$ of 122 segments | - | $\frac{3}{20}$ of 122 segments |  | 5 fold^b^ | No | DL | Polar | 2D | - | Segmentation | Frame 0.11 s | Yes^c^ | No |
| 65 |  | $\frac{17}{20}$ of 122 segments | - | $\frac{3}{20}$ of 122 segments |  | 5 fold^b^ | No | DL | Polar patches | 2D | - | Classification | Frame 0.003 s | Yes^c^ | No |
| 66 |  | 68 lesions  3,821 lesions | - | 9 lesions  539 frames |  | 5 fold | No | DL | Polar | 2D | - | Segmentation | - | Yes^c^ | No |
| 67 |  | NA | NA | NA |  | NA | NA | NA | NA | NA | NA | Prognosis | - | Yes^c^ | No |
| 68 |  | 27 patients  27 pullbacks | 9 patients  9 pullbacks | 9 patients  9 pullbacks |  | - | No | DL | Cartesian | 2D | - | Segmentation | - | No | No |
| 68 |  | 27 patients  27 pullbacks | 9 patients  9 pullbacks | 9 patients  9 pullbacks  (4 additional pullbacks used for calcium score) |  | - | No | DL | Cartesian | 2D and 3D  (16 frames) | - | Segmentation | Frame 0.023 s for segmentation | No | No |
| 68 |  | 27 patients  27 pullbacks | 9 patients  9 pullbacks | 9 patients  9 pullbacks  (4 additional pullbacks used for calcium score) |  | - | No | DL | Polar | 2D | - | Classification | - | No | No |
| 69 |  | 1,351 patients  35,958 frames | 338 patients  8,989 frames | 102 patients  1,173 frames |  | Not for classification, 10 fold for clinical outcome | No | DL | Polar | 2D | Greyscale | Classification  Prognosis | - | Yes^c^ | No |
| 70 |  | 21 patients  291,280 patches | - |  |  | 8 fold | No | DL | Polar patches | 2D | - | Segmentation | - | No | Yes |
| 71 |  | 581 patients  581 pullbacks  237,021 frames | - | - |  | 5 fold | 292 patients 65,934 frames | DL | Cartesian | 3D  (180 frames) | - | Classification | Lesion-level 0.80-1.04 s | No | No |
| 72 |  | 78 pullbacks  78 frames | - | 20 pullbacks  20 frames |  | 5 fold | No | DL | A-lines | 2D | - | Classification | Frame 0.07 s | Yes^c^ | Yes^c^ |
| 73 |  | 2,000 frames | - | 300 frames |  | 5 fold | No | DL | Both | 2D | - | Classification; Detection | - | No | No |
| 74 |  | 51 pullbacks  2,015 frames | 16 pullbacks  655 frames | 16 pullbacks  5,553 frames |  | - | No | DL | Cartesian | 2D | Greyscale | Segmentation; Classification | Frame 0.447 s | Yes^c^ | No |
| 75 |  | 44 patients  44 pullbacks  1,273 frames | 11 patients  11 pullbacks  379 frames | 15 patients  15 pullbacks  298 frames |  | - | No | DL | Cartesian patches and frames | Pseudo 3D  (± 3 frames) | RGB | Segmentation | Frame 11.31 ms | Yes^c^ | No |
| 76 |  | 581 patients  581 pullbacks  237,021 frames | - | - |  | 5 fold | 292 patients  65,934 frames | DL | Cartesian patches | 3D  (5 frames) | - | Classification | - | Yes^c^ | No |
| 77 |  | NA | NA | NA |  | NA | NA | NA | NA | NA | NA | Prognosis | Pullback 9.16 minutes | Yes^c^ | No |
| 78 |  | 284 pullbacks | - | 72 pullbacks |  | 5 fold | 47 patients  101 pullbacks | ML | - | 2D | - | Classification | - | No | No |
| 79 |  | NA | NA | NA |  | NA | NA | NA | NA | NA | NA | Software | - | No | No |
| 80 |  | 78 lesions | - | 32 lesions |  | 5 fold | No | ML | Cartesian | Pseudo 3D  (± 15 frames) | - | Regression | - | Yes^c^ | No |
| 81 |  | 37 patients  4,625 frames | - | 9 patients  1,156 frames |  | - | No | DL | Cartesian | 2D | - | Detection | Frame 25.2 s | Yes^c^ | No |
| 82 |  | NA | NA | NA |  | NA | NA | NA | NA | NA | NA | Software | Pullback 4 minutes (analysis) and 20 s (loading)  Frame 0.6 seconds (analysis) and 7.5 s editing | Yes^c^ | No |
| 83 |  | 4,000 frames | 1,200 frames | 1,300 frames |  | - | No | DL | Both | 2D | - | Classification | - | No | No |
| 84 |  | 114 frames |  |  |  | 5 fold | No | ML | - | Pseudo 3D | - | Classification; Prognosis | - | Yes^c^ | No |
| 85 |  | 2,149 frames  31,184 augmented frames | - | 200 frames |  | - | No | DL | Cartesian | 2D | - | Segmentation  Software | Frame 1.15 s (prediction only) | No | No |
| 86 |  | $\frac{9}{10}$ of 2,220 patches | - | $\frac{1}{10}$ of 2,220 patches |  | 10 fold^b^ | No | DL | Cartesian patches | 2D | - | Segmentation | - | Yes^c^ | No |
| 87 |  | 2,000 frames | - | 300 frames |  | 5 fold | No | DL | Polar | 2D | - | Detection | - | No | No |
| 88 |  | 5 frames | - | 46 frames |  | - | No | DL | Polar | 2D | - | Segmentation | - | Yes^c^ | No |
| 89 |  | $\frac{4}{5}$ of 2,388 frames | - | $\frac{1}{5}$ of 2,388 frames |  | - | No | DL | Cartesian | 2D | RGB | Segmentation | - | No | No |
| 89 |  | 9 patients | 2 patients | 3 patients |  | - | No | DL | Cartesian patches | 2D | - | Classification | - | No | No |
| 89 |  | - | - | - |  | 5 fold^b^ | No | DL | Polar | 2D | RGB | Segmentation | - | No | No |
| 90 |  | 4,124 frames  49,488 augmented frames | - | 1,500 frames |  | - | No | DL | - | 2D | RGB | Segmentation | - | No | No |
| 91 |  | 117 patients  291 pullbacks  89,969 frames | 16 patients  42 pullbacks  12,727 frames | 32 patients  94 pullbacks  11,996 frames |  | - | No | DL | Polar | 2D | - | Segmentation | - | No | No |

Overview of algorithm characteristics in included articles.
*DL Deep Learning; IQR interquartile range; ML Machine Learning; NA Not Applicable; RGB Red-Green-Blue; VOI Volume of Interest
^a^Training set includes validation set if cross-validation is applied. If validation set is listed separately, a distinct validation set is used.
^b^Test set included.
^c^Upon reasonable request.*

# **Supplementary Table 5 Model performances for lumen**

| No. | Reported set | Reference | Evaluation | ACC. (%) | Sens. (%) | Spec. (%) | PPV (%) | NPV (%) | F1 (%) | AUC-ROC | DICE | Jaccard | Definition |
| --- | --- | --- | --- | --- | --- | --- | --- | --- | --- | --- | --- | --- | --- |
|  | Classification |  |  |  |  |  |  |  |  |  |  |  |  |
| 43 | Validation folds | Expert | Frame | 98.2 | 95.9 | 98.5 | 90.0 | 99.4 | - | 0.963 | - | - | Abnormal lumen: intraluminal atherothrombotic material (protrusion) |
|  | Segmentation |  |  |  |  |  |  |  |  |  |  |  |  |
| 11 | Held-out test | Unknown | Pixel | - | - | - | - | - | - | - | 0.985  (IQR 0.979-0.988) | 0.970  (IQR 0.958-0.977) |  |
| 13 | Held-out test | Expert | Pixel | - | - | - | - | - | - | - | - | - |  |
| 14 | Held-out test | Expert | Pixel | - | 92 | 93 | - | - | - | - | - | - |  |
| 21 | Test folds | Expert | Pixel | - | 99 ± 1 | 99 ± 0.6 | - | - | 99 ± 0.8 | - | 0.98 ± 0.01 | - |  |
| 28 | Test folds | Expert | Pixel | 97.96 ± 0.03 | 90.56 ± 0.14 | - | - | - | - | - | 0.9329 ± 0.0008 | 0.8757 ± 0.0014 |  |
| 34 | Validation folds | Expert | Pixel | - | - | - | - | - | - | - | 0.97 | 0.95 |  |
| 47 | Held-out test | Expert | Pixel | - | 95.05 ± 6.69 | 99.66 ± 0.56 | - | - | - | - | 0.9731 ± 0.452 | - |  |
| 52 | Held-out test | Expert | Pixel | 98.2 | 96.4 | 98.6 | - | - | - | - | 0.957 | 0.923 |  |
| 58 | Held-out test | Unknown | Pixel | - | - | - | - | - | - | - | 0.988 ± 0.045 | - |  |
| 60 | Held-out test | Expert | Pixel | - | 93.97 | - | 96.86 | - | 95.40 | - | - | 0.9119 |  |
| 64 | External test | IVUS | Pixel | - | - | - | - | - | - | - | - | - |  |
| 68 | Held-out test | Unknown | Pixel | - | - | - | - | - | - | - | 0.986 ± 0.024 | - |  |
| 88 | Test folds | Expert | Pixel | - | - | - | - | - | - | - | 0.9424 | - |  |
| 89 | Held-out test | Expert | Pixel | - | - | - | - | - | - | - | >0.95 | - |  |

Performance analysis of lumen classification and segmentation in intracoronary OCT.
*Results on folds are reported as mean across folds unless reported otherwise. Results are reported as value ± standard deviation or value (95% confidence interval), if available unless reported otherwise.
ACC accuracy; AUC-ROC area under the receiver operating characteristic curve; IVUS intravascular ultrasound; NPV negative predictive value; PPV positive predictive value; SENS sensitivity; SPEC specificity*

# **Supplementary Table 6 Model performances for intima**

| No. | Reported set | | | REFerence | | Evaluation | | ACC. (%) | | Sens. (%) | | Spec. (%) | | PPV (%) | NPV (%) | | F1 (%) | | AUC-ROC | | DICE | | Jaccard | | Definition | |
| --- | --- | --- | --- | --- | --- | --- | --- | --- | --- | --- | --- | --- | --- | --- | --- | --- | --- | --- | --- | --- | --- | --- | --- | --- | --- | --- |
|  | | Classification |  | |  | |  | |  | |  | |  | | |  | |  | |  | |  | |  | |  |
| 8 | Held-out test | | | Expert | | ROI | | 92 | | 90 | | 94 | | - | - | | - | | - | | - | | - | | Intima (positive label) media (negative label) | |
|  | Segmentation | | | | | | | | | | | | | | | | | | | | | | | | | |
| 19 | Held-out test^a^ | | | Expert | | Pixel | | 90 ± 4 | | 93 ± 3 | | 86 ± 6 | | - | - | | 99 ± 1 | | - | | - | | - | |  | |
| 49 | Held-out test | | | Core lab | | Pixel | | - | | 98.9 | | - | | 98.9 | - | | - | | - | | 0.989 | | - | | Area between estimated IEL and lumen contour | |
| 88 | Test folds | | | Expert | | Pixel | | - | | - | | - | | - | - | | - | | - | | 0.9067 | | - | |  | |

Performance analysis of intima classification and segmentation in intracoronary OCT. *Results on folds are reported as mean across folds unless reported otherwise. Results are reported as value ± standard deviation or value (95% confidence interval), if available unless reported otherwise.
ACC accuracy; AUC-ROC area under the receiver operating characteristic curve; IEL internal elastic lamina; NPV negative predictive value; PPV positive predictive value; ROI region of interest; SENS sensitivity; SPEC specificity
^a^Only the non-pathological frames from the complete test dataset are used in the evaluation.*

# **Supplementary Table 7 Model performances for media**

| No. | Reported set | REFerence | Evaluation | acc. (%) | sens. (%) | spec. (%) | PPV (%) | NPV (%) | F1 (%) | AUC-ROC | DICE | Jaccard | Definition | |
| --- | --- | --- | --- | --- | --- | --- | --- | --- | --- | --- | --- | --- | --- | --- |
|  | Classification |  |  |  |  |  |  |  |  |  |  |  |  | |
| 8 | Held-out test | Expert | ROI | 92 | 94 | 90 | - | - | - | - | - | - | Intima (positive label) media (negative label) | |
|  | Segmentation |  |  |  |  |  |  |  |  |  |  |  |  | |
| 19 | Held-out test^a^ | Expert | Pixel | 87 ± 4 | 91 ± 2 | 82 ± 5 | - | - | 99 ± 1 | - | - | - |  | |
| 88 | Test folds | Expert | Pixel | - | - | - | - | - | - | - | 0.9108 | - |  |  |

Performance analysis of media classification and segmentation in intracoronary OCT.
*Results on folds are reported as mean across folds unless reported otherwise. Results are reported as value ± standard deviation or value (95% confidence interval), if available unless reported otherwise.
ACC accuracy; AUC-ROC area under the receiver operating characteristic curve; NPV negative predictive value; PPV positive predictive value; SENS sensitivity; SPEC specificity
^a^Only the non-pathological frames from the complete test dataset are used in the evaluation.*

# **Supplementary Table 8 Model performances for sidebranch**

| No. | Reported set | | | REFerence | | Evaluation | | acc. (%) | | Sens. (%) | | | Spec. (%) | | PPV (%) | | NPV (%) | | F1 (%) | | AUC-ROC | | DICE | | Jaccard | Definition |
| --- | --- | --- | --- | --- | --- | --- | --- | --- | --- | --- | --- | --- | --- | --- | --- | --- | --- | --- | --- | --- | --- | --- | --- | --- | --- | --- |
|  | | Classification |  | |  | |  | |  | |  |  | |  | |  | |  | |  | |  | |  | | |
| 4 | Validation folds | | | Expert | | Frame | | 93.42 ± 0.94 | | 98.05 ± 1.62 | | | 60.07 ± 11.41 | | 94.69 ± 1.45 | | - | | 96.32 ± 0.53 | | 0.9436 ± 0.0419 | | - | | - |  |
| 26 | Validation folds | | | Expert | | Frame | | 97.93 ± 1.00 | | 98.48 ± 0.98 | | | 98.41 ± 1.11 | | 97.46 ± 1.30 | | - | | 99.68 ± 0.31 | | 0.9972 ± 0.0017 | | - | | - |  |
|  | | Segmentation |  | |  | |  | |  | |  |  | |  | |  | |  | |  | |  | |  | | |
| 49 | Held-out test | | | Core lab | | Pixel | | - | | 89.0 | | | - | | 81.5 | | - | | - | | - | | 0.851 | | - |  |

Performance analysis of sidebranch classification and segmentation in intracoronary OCT.
*Results on folds are reported as mean across folds unless reported otherwise. Results are reported as value ± standard deviation or value (95% confidence interval), if available unless reported otherwise.
ACC accuracy; AUC-ROC area under the receiver operating characteristic curve; NPV negative predictive value; PPV positive predictive value; SENS sensitivity; SPEC specificity*

# **Supplementary Table 9 Model performances for lipid plaques**

| No. | Reported set | REFerence | Evaluation | Acc. (%) | Sens. (%) | Spec. (%) | PPV (%) | NPV (%) | F1 (%) | AUC-ROC | DICE | Jaccard | Definition |
| --- | --- | --- | --- | --- | --- | --- | --- | --- | --- | --- | --- | --- | --- |
|  | Classification |  |  |  |  |  |  |  |  |  |  |  |  |
| 18 | Test folds | Core lab | A-line | 89.8 | 85.6 | 90.9 | 71.5 | 95.9 | 77.9 | - | **-** | - | Fibrolipidic |
| 20 | Held-out test | Expert | Frame | 84.8 (weighted) | - | - | - | - | - | - | **-** | - | Fibrolipidic |
| 27 | Held-out test | Expert | A-line | - | 94.48 | 87.32 | - | - | - | - | **-** | - | Fibrolipidic |
| 29 | Validation folds | Expert | Frame | 90.63 | 87.50 | 93.75 | - | - | - | - | **-** | - | Fibroatheroma |
| 30 | Test folds | Expert | Plaque | 99.2 | - | - | - | - | - | - | **-** | - |  |
| 37 | Held-out test | Core lab | A-line | 95.5 | 77.3 | 98.9 | 93.3 | 95.9 | 84.5 | - | **-** | - | Fibrolipidic |
| 45 | Held-out test | Expert | Frame | 99 | 99 | 99 | - | - | - | - | **-** | - |  |
| 48 | Test folds (best) | Expert | A-line | 75.4 | 67.5 | 78.1 | 50.6 | 87.8 | 57.9 | - | **-** | - |  |
| 53 | Held-out test | Expert^a^ | Polar patch | - | 67 | 85 | 67 | 85 | - | 0.82 | **-** | - |  |
| 72 | Held-out test | Histology | A-line | 89.6 ± 0.01 | 83.6 ± 0.01 | 91.1 ± 0.01 | - | - | - | - | **-** | - |  |
| 89 | Held-out test | Expert | Frame | 98.10 | 100 | 93.88 | 97.10 | - | 98.53 | 0.9799 | **-** | - |  |
|  | Segmentation |  |  |  |  |  |  |  |  |  |  |  |  |
| 2 | Held-out test | Expert | Pixel | - | 79.5 | - | - | - | - | - | - | - |  |
| 3 | Held-out test | Expert | Pixel | - | 71 | - | 76 | - | - | - | - | - |  |
| 10 | Validation folds | Expert | Pixel | 96.40 ± 8.887 | - | - | - | - | - | - | - | - |  |
| 10 | External test | Histology | Pixel | 87.65 | - | - | - | - | - | - | - | - |  |
| 17 | Test folds | Expert | Pixel | 94.1 | 93.5 | 94.5 | 91.8 | 95.7 | 92.7 | - | - | - |  |
| 22 | Test folds | Core lab | Pixel | - | 87.4 ± 7.2 | 89.5 ± 4.6 | - | - | - | - | 0.801 ± 0.044 | 0.690 ± 0.058 | Fibrolipidic |
| 22 | Test folds | Core lab | A-line | - | 90.1 ± 3.9 | 84.3 ± 6.6 | - | - | - | - | 0.827 ± 0.057 | 0.733 ± 0.073 | Fibrolipidic |
| 31 | Held-out test | Expert | Pixel | 79.6 ± 3.9 | - | - | - | - | - | - | - | - | Calcified/lipid plaque |
| 32 | Test folds | Expert^b^ | Pixel | 74.14 | - | - | - | - | - | - | - | - |  |
| 44 | Validation folds | Expert^b^ | Pixel | 82.84 | - | - | - | - | - | - | - | - |  |
| 49 | Held-out test | Core lab | Pixel | - | 80.7 | - | 73.9 | - | - | - | 0.772 | - |  |
| 49 | External test | Core lab^c^ | Cartesian region | 90.5 (85.2-94.1) | - | - | - | - | - | - | - | - | >80% overlap between ground-truth region and segmentation |
| 51 | Test folds | Histology | Pixel | - | - | - | - | - | - | - | - | 0.68 ± 0.18 | Atherosclerotic lesion |
| 51 | Test folds | Histology | A-line | 88 | - | - | - | - |  | - | - | - | Atherosclerotic lesion  >3 pixels along A-line |
| 51 | External test | Expert | Frame | 85.8 ± 7.7 | 86.6 ± 8.9 | 82.9 ± 8.7 | - | - | - | - | - | - | Atherosclerotic lesion |
| 56 | Held-out test | Histology | Frame | - | 75 | 93 | 68 | 95 | 62 | 0.86 | - | - |  |
| 56 | Held-out test | Histology | Pixel | - | - | - | - | - | - | - | - | 0.45 |  |
| 59 | Validation folds | Expert | Pixel | - | 81 ± 11 | 96 ± 3 | 89 ± 13 | - | - | - | - | - |  |
| 66 | Held-out test | Core lab | A-line | - | 85.8 | 90.7 | 81.8 | - | - | - | 0.837 | - |  |
| 70 | Validation folds (pooled) | Expert | Patch | 91.5 | 77.9 | 95.1 | 80.6 | 94.3 | 79.2 | - | - | - |  |
| 86 | Test folds | Expert | Pixel | 91.8 ± 1.6 | 91.5 ± 1.1 | 94.4 ± 2.8 | - | - | - | - | - | - |  |
| 89 | Test folds | Expert | Pixel | - | 72.656 | 89.832 | - | - | - | 0.85156 | 0.56526 | - |  |
| 90 | Held-out test | Expert | Pixel | - | 80 | - | 82 | - | 81 | - | - | - |  |

Performance analysis of lipid plaque classification and segmentation in intracoronary OCT.
*Results on folds are reported as mean across folds unless reported otherwise. Results are reported as value ± standard deviation or value (95% confidence interval), if available unless reported otherwise.
ACC accuracy; AUC-ROC area under the receiver operating characteristic curve; NPV negative predictive value; PPV positive predictive value; SENS sensitivity; SPEC specificity
^a^Using intravascular ultrasound-near-infrared spectroscopy (IVUS-NIRS) as reference.
^b^Using virtual-histology intravascular ultrasound (VH-IVUS) as reference.
^c^Independent, external core laboratory.*

# **Supplementary Table 10 Model performances for high-risk plaques**

| No. | Reported set | REFErence | Evaluation | Acc. (%) | Sens. (%) | Spec. (%) | PPV (%) | NPV (%) | F1 (%) | AUC-ROC | DICE | Jaccard | Definition | |
| --- | --- | --- | --- | --- | --- | --- | --- | --- | --- | --- | --- | --- | --- | --- |
|  | Classification |  |  |  |  |  |  |  |  |  |  |  |  | |
| 40 | Validation folds | Expert | Frame | 91.6 ± 1.7 | 88.7 ± 3.4 | 91.8 ± 2.0 | 47.1 ± 5.9 | 99.0 ± 0.3 | - | 0.963 ± 0.008 | - | - | TCFA (fibrous cap <65 µm, lipid angle ≥90°) | |
| 40 | Held-out test | Expert | Frame | 92.8 | 88.6 | 93.2 | 50.8 | 99.0 | - | 0.962 | - | - |  | |
| 40 | External test | Expert | Frame | 90 | 94 | 90 | 28 | 99 | - | 0.97 | - | - |  | |
| 41 | Held-out test | Expert | Frame | 96.65 | - | - | - | - | - | - | - | - | Vulnerable plaque | |
| 53 | Held-out test | IVUS-NIRS | Polar patch | - | 90.5 | 84.2 | - | - | - | - | - | - | Max LCBI 4mm > 400 | |
| 69 | Held-out test | Expert | Frame | 96.3 | - | - | - | - | - | 0.998 | - | - | Vulnerable plaque (fibrous cap <100 µm, lipid arc >90°) | |
| 73 | Held-out test | Expert | A-line | - | 92.18 ± 4.5 | - | 75.74 ± 3.19 | - | - | - | - | - | TCFA | |
| 73 | Held-out test | Expert | Frame | - | 92.42 | - | 91.95 | - | 92.19 | 0.9210 | - | - | TCFA | |
| 83 | Held-out test | Expert | Frame | 93.51 ± 0.53 | 94.12 ± 1.38 | - | 92.86 ± 1.70 | - | 93.68 ± 0.69 | - | - | - | TCFA (fibrous cap <65 um) | |
|  | Detection |  |  |  |  |  |  |  |  |  |  |  |  | |
| 24 | Held-out test | Expert | Bounding box | - | 95.02 | - | 88.84 | - | - | 0.867 | - | - | TCFA (fibrous cap <65 um infiltrated by macrophages)  True positive if Dice >0.5 | |
| 57 | Held-out test | Unknown | Bounding box | 71.6 | 94.1 | - | - | - | - | - | - | - | Vulnerable plaque  True positive IoU >0.5 | |
| 73 | Held-out test | Expert | Bounding box | - | 93.67 ± 2.29 | - | 84.34 ± 0.86 | - | - | - | - | - | TCFA  True positive if Dice ≥0.5 | |
| 87 | Validation folds | Expert | Bounding box | - | 87.96 ± 0.21 | - | 83.29 ± 0.12 | - | - | - | - | - | TCFA  True positive if Dice ≥0.5 | |
| 87 | Held-out test | Expert | Bounding box | - | 88.42 | - | 84.00 | - | - | - | - | - |  |  |
|  | Segmentation |  |  |  |  |  |  |  |  |  |  |  |  | |
| 23 | Held-out test | Expert | Pixel | 93.31 | 91.35 | 94.33 | - | - | - | - | - | - | Vulnerable plaque | |

Performance analysis of high-risk plaque classification, detection and segmentation in intracoronary OCT.
*Results on folds are reported as mean across folds unless reported otherwise. Results are reported as value ± standard deviation or value (95% confidence interval), if available unless reported otherwise.
ACC accuracy; AUC-ROC area under the receiver operating characteristic curve; IoU intersection over union; IVUS-NIRS intravascular ultrasound-near-infrared spectroscopy; LCBI lipid core burden index; NPV negative predictive value; PPV positive predictive value; SENS sensitivity; SPEC specificity; TCFA thin-cap fibroatheroma*

# **Supplementary Table 11 Model performances for calcified plaques**

| No. | Reported set | REFerence | Evaluation | Acc. (%) | Sens. (%) | Spec. (%) | PPV (%) | NPV (%) | F1 (%) | AUC-ROC | DICE | Jaccard | Definition |
| --- | --- | --- | --- | --- | --- | --- | --- | --- | --- | --- | --- | --- | --- |
|  | Classification |  |  |  |  |  |  |  |  |  |  |  |  |
| 18 | Test folds | Core lab | A-line | 91.7 | 79.6 | 94.1 | 72.4 | 95.9 | 75.9 | - | - | - | Fibrocalcific |
| 19 | Held-out test^a^ | Expert | Unknown | 90 | 84 | 95 | - | - | - | - | - | - |  |
| 20 | Held-out test | Expert | Frame | 78.0 (weighted) | - | - | - | - | - | - | - | - |  |
| 27 | Held-out test | Expert | A-line | - | 74.82 | 95.28 | - | - | - | - | - | - | Fibrocalcific |
| 30 | Test folds | Expert | Plaque | 97.2 | - | - | - | - | - | - | - | - |  |
| 35 | Test folds | Expert | Pullback | - | 97.7 ± 3.4 | 96.9 ± 1.3 | - | - | 96.1 ± 3.4 | - | - | - |  |
| 37 | Held-out test | Core lab | A-line | 92.8 | 97.2 | 91.9 | 70.3 | 99.4 | 81.6 | - | - | - | Fibrocalcific |
| 38 | Test folds^b^ | Core lab | Frame | - | 97.7 ± 2.4 | - | 87.7 ± 2.1 | - | 92.2 ± 2.1 | - | - | - |  |
| 45 | Held-out test | Expert | Frame | 99 | 98 | 99 | - | - | - | - | - | - |  |
| 48 | Test folds (best) | Expert | A-line | 73.4 | 37.6 | 85.4 | 46.1 | 80.4 | 41.4 | - | - | - |  |
| 53 | Held-out test | Expert^c^ | Polar patch |  | 69 | 97 | 64 | 98 | - | 0.93 | - | - |  |
| 68 | Held-out test | Expert | Frame | 96.5 ± 0.2 | 81.5 ± 1.3 | - | 96.4 ± 0.2 | - | 88.3 ± 0.8 | - | - | - | True positive if Dice ≥0.5 |
| 89 | Held-out test | Expert | Frame | 100 | 100 | 100 | 100 | - | 100 | 1.00 | - | - |  |
|  | Segmentation |  |  |  |  |  |  |  |  |  |  |  |  |
| 2 | Held-out test | Expert | Pixel | 72.1 | - | - | - | - | - | - | - | - |  |
| 3 | Held-out test | Expert | Pixel | - | 81 | - | 83 | - | - | - | - | - |  |
| 10 | Validation folds | Expert | Pixel | 92.14 ± 10.74 | - | - | - | - | - | - | - | - |  |
| 10 | External test | Histology | Pixel | 97.62 | - | - | - | - | - | - | - | - |  |
| 17 | Test folds | Expert | Pixel | 91.1 | 83.1 | 94.3 | 85.8 | 93.1 | 84.5 | - | - | - |  |
| 21 | Test folds | Expert | Pixel | - | 85 ± 4 | 99 ± 0.4 | - | - | 73 ± 1 | - | 0.76 ± 0.03 | - |  |
| 22 | Test folds | Core lab | Pixel | - | 85.1 ± 7.2 | 94.2 ± 2.4 | - | - | - | - | 0.734 ± 0.085 | 0.594 ± 0.064 | Fibrocalcific |
| 22 | Test folds | Core lab | A-line | - | 92.9 ± 4.5 | 76.4 ± 6.2 | - | - | - | - | 0.897 ± 0.038 | 0.827 ± 0.057 | Fibrocalcific |
| 31 | Held-out test | Expert | Pixel | 79.6 ± 3.9 | - | - | - | - | - | - | - | - | Calcified/lipid plaque |
| 38 | Test folds^b^ | Core lab | Pixel | - | 86.2 ± 2.0 | - | 75.8 ± 8.8 | - | 78.1 ± 2.0 | - | - | - |  |
| 46 | Held-out test | Expert | Pixel | 99.03 ± 0.9 | - | - | - | - | - | - | 0.7143 ± 0.2609 | - |  |
| 49 | Held-out test | Core lab | Pixel | - | 88.8 | - | 81.1 | - | - | - | 0.848 | - |  |
| 49 | External test | Core lab^d^ | Cartesian region | 88.5 (82.4-92.7) | - | - | - | - | - | - | - | - | True positive if >80% overlap with ground-truth |
| 51 | Test folds | Histology | Pixel | - | - | - | - | - | - | - | - | 0.34 |  |
| 54 | Test folds | Core lab | Pixel | 94 ± 2 | 69 ± 7 | 98 ± 1 | - | - | 69 ± 4 | - | - | - |  |
| 56 | Held-out test | Histology | Pixel | - | - | - | - | - | - | - | - | 0.51 | Fibrocalcific |
| 56 | Held-out test | Histology | Frame | - | 78 | 88 | 91 | 89 | 67 | 0.91 | - | - | Fibrocalcific |
| 59 | Validation folds | Expert | Pixel | - | 87 ± 6 | 92 ± 6 | 68 ± 12 | - | - | - | - | - |  |
| 61 | Held-out test | Expert | Pixel | - | 88.95 | - | 93.95 | - | 91.38 | - | - | - |  |
| 61 | Held-out test | Expert | Frame | 91.0 (83-95) | 91.7 (82.7-96.9) | 89.3 (71.8-97.7) | 95.7 | 80.6 | 93.6 | - | - | - | Calcified nodule  True positive if >80% overlap with ground truth |
| 68 | Held-out test | Expert | Pixel | - | - | - | - | - | - | - | 0.756 ± 0.222 | - |  |
| 68 | Held-out test | Expert | Frame | 92.4 ± 2.0 | 87.4 ± 0.4 | - | 72.5 ± 7.1 | - | 79.1 ± 4.1 | - | - | - | True positive if Dice ≥0.5 |
| 70 | Validation folds (pooled) | Expert | Patch | 86.9 | 67.8 | 91.5 | 65.9 | 92.2 | 66.8 | - | - | - |  |
| 85 | Held-out test | Expert | Pixel | 83 | - | - | - | - | - | - | 0.72 | 0.59 |  |
| 86 | Test folds | Expert | Pixel | 91.9 ± 3.6 | 92.3 ± 3.4 | 92.5 ± 3.6 | - | - | - | - | - | - |  |
| 89 | Test folds | Expert | Pixel | - | 74.616 | 95.96 | - | - | - | 0.88686 | 0.6176 | - |  |
| 90 | Held-out test | Expert | Pixel | - | 83 | - | 83 | - | 83 | - | - | - |  |

Performance analysis of calcified plaque classification and segmentation in intracoronary OCT.
*Results on folds are reported as mean across folds unless reported otherwise. Results are reported as value ± standard deviation or value (95% confidence interval), if available unless reported otherwise.
ACC accuracy; AUC-ROC area under the receiver operating characteristic curve; NPV negative predictive value; PPV positive predictive value; SENS sensitivity; SPEC specificity
^a^Only the pathological frames from the complete test dataset are used in the evaluation. ^b^Only the in vivo dataset from the complete test dataset is used in the evaluation.
^c^Using intravascular ultrasound-near-infrared spectroscopy (IVUS-NIRS) as reference.
^d^Independent, external core laboratory.*

# **Supplementary Table 12 Model performances for fibrous plaques**

| No. | Reported set | REFERENCE | Evaluation | Acc. (%) | Sens. (%) | Spec. (%) | PPV (%) | NPV (%) | F1 (%) | AUC-ROC | DICE | Jaccard | Definition |
| --- | --- | --- | --- | --- | --- | --- | --- | --- | --- | --- | --- | --- | --- |
|  | Classification |  |  |  |  |  |  |  |  |  |  |  |  |
| 19 | Held-out test^a^ | Expert | Unknown | 94 | 94 | 96 | - | - | - | - | - | - |  |
| 30 | Test folds | Expert | Plaque | 94.0 | - | - | - | - | - | - | - | - |  |
| 45 | Held-out test | Expert | Frame | 96 | 94 | 99 | - | - | - | - | - | - |  |
| 48 | Test folds (best) | Expert | A-line | 81.6 | 55.0 | 90.5 | 65.8 | 85.8 | 59.9 | - | - | - |  |
|  | Segmentation |  |  |  |  |  |  |  |  |  |  |  |  |
| 2 | Held-out test | Expert | Pixel | 89.5 | - | - | - | - | - | - | - | - |  |
| 3 | Held-out test | Expert | Pixel | - | 87 | - | 78 | - | - | - | - | - |  |
| 10 | Validation folds | Expert | Pixel | 100 ± 0.0 | - | - | - | - | - | - | - | - |  |
| 10 | External test | Histology | Pixel | 97.39 | - | - | - | - | - | - | - | - |  |
| 17 | Test folds | Expert | Pixel | 93.0 | 89.0 | 94.8 | 88.5 | 95.0 | 88.7 | - | - | - |  |
| 20 | Held-out test | Expert | Frame | 84.8 (weighted) | - | - | - | - | - | - | - | - | Fibrolipidic |
| 31 | Held-out test | Expert | Pixel | - | - | - | - | - | - | - | - | 0.814 ± 0.033 |  |
| 32 | Validation folds | Expert^b^ | Pixel | 81.92 | - | - | - | - | - | - | - | - |  |
| 49 | Held-out test | Core lab | Pixel | - | 88.1 | 93.2 | - | - | - | - | 0.906 | - |  |
| 49 | External test | Core lab^c^ | Cartesian region | 97.6 (93.4-99.3) | - | - | - | - | - | - | - | - | True positive if >80% overlap with ground-truth |
| 56 | Held-out test | Histology | Pixel | - | - | - | - | - | - | - | - | 0.34 | Pathological intimal thickening |
| 56 | Held-out test | Histology | Frame | - | 74 | 85 | 73 | 85 | - | 0.85 | - | - | Pathological intimal thickening |
| 59 | Validation folds | Expert | Pixel | - | 95 ± 3 | 97 ± 2 | 96 ± 3 | - | - | - | - | - |  |
| 70 | Validation folds (pooled) | Expert | Patch | 97.8 | 94.5 | 98.6 | 94.2 | 98.6 | 94.4 | - | - | - |  |
| 86 | Test folds | Expert | Pixel | 92.8 ± 2.7 | 91.2 ± 1.2 | 91.5 ± 2.0 | - | - | - | - | - | - |  |
| 90 | Held-out test | Expert | Pixel | - | 82 | - | 84 | - | 83 | - | - | - |  |

Performance analysis of fibrous plaque classification and segmentation in intracoronary OCT.
*Results on folds are reported as mean across folds unless reported otherwise. Results are reported as value ± standard deviation or value (95% confidence interval), if available unless reported otherwise.
ACC accuracy; AUC-ROC area under the receiver operating characteristic curve; NPV negative predictive value; PPV positive predictive value; SENS sensitivity; SPEC specificity
^a^Only the pathological frames from the complete test dataset are used in the evaluation.
^b^Using virtual-histology intravascular ultrasound (VH-IVUS) as reference.
^c^Independent, external core laboratory.*

# **Supplementary Table 13 Model performances for plaque rupture**

| No. | Reported set | REFerence | Evaluation | Acc. (%) | Sens. (%) | Spec. (%) | PPV (%) | NPV (%) | F1 (%) | AUC-ROC | DICE | Jaccard | Definition |
| --- | --- | --- | --- | --- | --- | --- | --- | --- | --- | --- | --- | --- | --- |
|  | Classification |  |  |  |  |  |  |  |  |  |  |  |  |
| 71 | Validation folds | Core lab | Frame | - | 74.3 (73.4-75.2) | 96.4 (96.3-96.5) | 44.6 (44.0-45.2) | 99.0 (98.9-99.0) | - | 0.98 (0.98-0.98) | - | - |  |
| 71 | Validation folds | Core lab | Lesion | - | 83.3 (78.8-87.2) | 84.9 (79.8-89.1) | 87.8 (84.3-90.7) | 79.5 (75.2-83.2) | - | 0.90 (0.88-0.93) | - | - |  |
| 71 | External test | Core lab | Frame | - | 81.8 (80.6-83.1) | 95.7 (95.6-95.9) | 53.4 (52.4-54.4) | 98.9 (98.8-99.0) | - | 0.96 (0.96-0.96) | - | - |  |
| 71 | External test | Core lab | Lesion | - | 81.6 (75.6-86.6) | 91.9 (84.0-96.7) | 96.0 (92.2-98.0) | 67.5 (60.8-73.6) | - | 0.91 (0.88-0.94) | - | - |  |

Performance analysis of plaque rupture classification in intracoronary OCT.
*Results on folds are reported as mean across folds unless reported otherwise. Results are reported as value ± standard deviation or value (95% confidence interval), if available unless reported otherwise.
ACC accuracy; AUC-ROC area under the receiver operating characteristic curve; NPV negative predictive value; PPV positive predictive value; SENS sensitivity; SPEC specificity*

# **Supplementary Table 14 Model performances for plaque erosion**

| No. | Reported set | REference | Evaluation | Acc. (%) | Sens. (%) | Spec. (%) | PPV (%) | NPV (%) | F1 (%) | AUC-ROC | | DICE | Jaccard | Definition |
| --- | --- | --- | --- | --- | --- | --- | --- | --- | --- | --- | --- | --- | --- | --- |
|  | Classification |  |  |  |  |  |  |  |  | |  |  |  |  |
| 71 | Validation folds | Core lab | Frame | - | 84.9 (78.5-90.0) | 86.8 (83.1-89.9) | 71.8 (66.4-76.7) | 93.5 (90.9-95.4) | - | | 0.93 (0.91-0.95) | - | - | Definite or probable plaque erosion |
| 71 | Validation folds | Core lab | Lesion | - | 82.1 (76.8-86.6) | 84.5 (80.1-88.2) | 80.2 (75.7-84.0) | 86.1 (82.5-89.0) | - | | 0.90 (0.88-0.93) | - | - |  |
| 71 | External test | Core lab | Frame | - | 92.7 (80.1-98.5) | 88.5 (83.8-92.1) | 56.7 (47.9-65.1) | 98.7 (96.1-99.6) | - | | 0.94 (0.91-0.97) | - | - |  |
| 71 | External test | Core lab | Lesion | - | 91.9 (84.0-96.7) | 81.6 (75.6-86.6) | 67.5 (60.8-73.6) | 96.0 (92.2-98.0) | - | | 0.91 (0.88-0.94) | - | - |  |
| 74 | Held-out test | Expert | Frame | - | 80.0 ± 17.5 | 73.4 ± 25.4 |  |  | - | |  | - | - | Definite plaque erosion  True positive if ≥50% overlap with ground truth |

Performance analysis of plaque erosion classification in intracoronary OCT.
*Results on folds are reported as mean across folds unless reported otherwise. Results are reported as value ± standard deviation or value (95% confidence interval), if available unless reported otherwise.
ACC accuracy; AUC-ROC area under the receiver operating characteristic curve; NPV negative predictive value; PPV positive predictive value; SENS sensitivity; SPEC specificity*

# **Supplementary Table 15 Model performances for layered plaques**

| No. | Reported set | REFerence | Evaluation | Acc. (%) | Sens. (%) | Spec. (%) | PPV (%) | NPV (%) | F1 (%) | AUC-ROC | DICE | Jaccard | Definition |
| --- | --- | --- | --- | --- | --- | --- | --- | --- | --- | --- | --- | --- | --- |
|  | Classification |  |  |  |  |  |  |  |  |  |  |  |  |
| 76 | Validation folds | Core lab | - | 77.6 (77.4-77.8) | 77.7 (76.4-79.0) | 77.6 (77.4-77.8) | - | - | - | 0.860 (0.855-0.866) | - | - |  |
| 76 | External test | Core lab | - | 76.0 (75.7-76.3) | 76.5 (74.6-78.4) | 76.0 (75.7-76.3) | - | - | - | 0.845 (0.837-0.853) | - | - |  |
|  | Segmentation |  |  |  |  |  |  |  |  |  |  |  |  |
| 56 | Held-out test | Histology | Pixel | - | - | - | - | - | - | - | - | 0.36 |  |
| 56 | Held-out test | Histology | Frame | - | 67 | 96 | 53 | 96 | - | 0.86 | - | - |  |

Performance analysis of layered plaque classification and segmentation in intracoronary OCT.
*Results on folds are reported as mean across folds unless reported otherwise. Results are reported as value ± standard deviation or value (95% confidence interval), if available unless reported otherwise.
ACC accuracy; AUC-ROC area under the receiver operating characteristic curve; NPV negative predictive value; PPV positive predictive value; SENS sensitivity; SPEC specificity*

# **Supplementary Table 16 Model performances for undefined plaques**

| No. | Reported set | REFerence | Evaluation | Acc. (%) | Sens. (%) | Spec. (%) | PPV (%) | NPV (%) | F1 (%) | AUC-ROC | DICE | Jaccard | Definition |
| --- | --- | --- | --- | --- | --- | --- | --- | --- | --- | --- | --- | --- | --- |
|  | Classification |  |  |  |  |  |  |  |  |  |  |  |  |
| 20 | Held-out test | Expert | Frame | 91.7 | 90.9 | 92.4 | - | - | 91.3 | - | - | - | Plaque |
| 48 | Test folds (best) | Expert | A-line | 74.73 ± 6.55 | 87.78 ± 8.79 | 61.45 ± 8.41 | - | - | - | - | - | - | Plaque |
| 48 | Test folds (best) | Expert | A-line | 76.5 | 53.8 | 84.0 | 52.9 | 84.5 | 53.4 | - | - | - | Mixed plaque |
| 69 | Held-out test | Expert | Frame | 86.9 | - | - | - | - | - | 0.952 | - | - | Stable plaque: fibrous, fibrocalcific (calcification arc ≤90°), thick-cap fibroatheroma (fibrous cap thickness >100µm) |
|  | Segmentation |  |  |  |  |  |  |  |  |  |  |  |  |
| 19 | Held-out test | Expert | Pixel | 96 ± 4 | 97 ± 3 | 95 ± 5 | - | - | 96 ± 4 | - | - | - | Pathological frames |
| 45 | Held-out test | Expert | Pixel | 93 ± 10 | 90 ± 13 | 95 ± 5 | - | - | 84 ± 18 | - | - | - | Atherosclerotic tissue |
| 70 | Validation folds (pooled) | Expert | Patch | 90.2 | 76.3 | 93.5 | 74.0 | 94.3 | 75.1 | - | - | - | Mixed plaque |

Performance analysis of undefined plaque classification and segmentation in intracoronary OCT.
*Results are reported as value ± standard deviation or value (95% confidence interval), if available unless reported otherwise.
ACC accuracy; AUC-ROC area under the receiver operating characteristic curve; NPV negative predictive value; PPV positive predictive value; SENS sensitivity; SPEC specificity*

# **Supplementary Table 17 Model performances for thrombus**

| No. | Reported set | REFerence | Evaluation | Acc.(%) | Sens. (%) | Spec. (%) | PPV (%) | NPV (%) | F1 (%) | AUC-ROC | DICE | Jaccard | Definition |
| --- | --- | --- | --- | --- | --- | --- | --- | --- | --- | --- | --- | --- | --- |
|  | Classification |  |  |  |  |  |  |  |  |  |  |  |  |
| 45 | Held-out test | Expert | Frame | 98 | 97 | 99 | - | - | - | - | - | - |  |

Performance analysis of thrombus classification in intracoronary OCT.
*ACC accuracy; AUC-ROC area under the receiver operating characteristic curve; NPV negative predictive value; PPV positive predictive value; SENS sensitivity; SPEC specificity*

# **Supplementary Table 18 Model performances for macrophage accumulation**

| No. | Reported set | REFerence | Evaluation | Acc. (%) | Sens. (%) | Spec. (%) | PPV (%) | NPV (%) | F1 (%) | AUC-ROC | DICE | Jaccard | Definition |
| --- | --- | --- | --- | --- | --- | --- | --- | --- | --- | --- | --- | --- | --- |
|  | Classification |  |  |  |  |  |  |  |  |  |  |  |  |
| 19 | Held-out test^a^ | Expert | Unknown | 92 | 89 | 97 | - | - | - | - | - | - |  |
|  | Segmentation |  |  |  |  |  |  |  |  |  |  |  |  |
| 49 | Held-out test | Core lab | Pixel | - | 56.8 | - | 42.9 | - | - | - | 0.489 | - |  |
| 49 | External test | Core lab^b^ | Cartesian region | 48.1 (37.3-59.0) | - | - | - | - | - | - | - | - | True positive if >80% overlap with ground-truth |

Performance analysis of macrophage accumulation classification and segmentation in intracoronary OCT.
*Results are reported as value ± standard deviation or value (95% confidence interval), if available unless reported otherwise.
ACC accuracy; AUC-ROC area under the receiver operating characteristic curve; NPV negative predictive value; PPV positive predictive value; SENS sensitivity; SPEC specificity
^a^Only the pathological frames from the complete test dataset are used in the evaluation.
^b^Independent, external core laboratory.*

# **Supplementary Table 19 Model performances for cholesterol clefts**

| No. | Reported set | REFerence | Evaluation | Acc. (%) | Sens. (%) | Spec. (%) | PPV (%) | NPV (%) | F1 (%) | AUC-ROC | DICE | Jaccard | Definition |
| --- | --- | --- | --- | --- | --- | --- | --- | --- | --- | --- | --- | --- | --- |
|  | Segmentation |  |  |  |  |  |  |  |  |  |  |  |  |
| 49 | Held-out test | Core lab | Pixel | - | 50.8 | - | 54.3 | - | - | - | 0.525 | - |  |
| 49 | External test | Core lab^a^ | Cartesian region | 94.7 (73.5-100) | - | - | - | - | - | - | - | - | True positive if >80% overlap with ground-truth |

Performance analysis of cholesterol clefts segmentation in intracoronary OCT.
*Results are reported as value ± standard deviation or value (95% confidence interval), if available unless reported otherwise.
ACC accuracy; AUC-ROC area under the receiver operating characteristic curve; NPV negative predictive value; PPV positive predictive value; SENS sensitivity; SPEC specificity
^a^Independent, external core laboratory.*

# **Supplementary Table 20 Model performances for microvessels**

| No. | Reported set | REFerence | Evaluation | Acc. (%) | Sens. (%) | Spec. (%) | PPV (%) | NPV (%) | F1 (%) | AUC-ROC | DICE | Jaccard | Definition |
| --- | --- | --- | --- | --- | --- | --- | --- | --- | --- | --- | --- | --- | --- |
|  | Classification |  |  |  |  |  |  |  |  |  |  |  |  |
| 19 | Held-out test^a^ | Expert | Unknown | 95 | 90 | 97 | - | - | - | - | - | - |  |
| 45 | Held-out test | Expert | Frame | 90 | 80 | 100 | - | - | - | - | - | - |  |
|  | Segmentation |  |  |  |  |  |  |  |  |  |  |  |  |
| 49 | Held-out test | Core lab | Pixel | - | 60.4 | - | 59.8 | - | - | - | 0.601 | - |  |
| 65 | Test folds (average) | Core lab | Pixel | - | 85.5 ± 6.9 | 99.8 ± 0.1 | - | - | - | - | 0.73 ± 0.10 | - |  |
| 75 | Held-out test | Expert | Pixel | - | 64.4 | - | - | - | - | - | 0.548 | - |  |

Performance analysis of microvessel classification and segmentation in intracoronary OCT.
*Results are reported as value ± standard deviation or value (95% confidence interval), if available unless reported otherwise.
ACC accuracy; AUC-ROC area under the receiver operating characteristic curve; NPV negative predictive value; PPV positive predictive value; SENS sensitivity; SPEC specificity
^a^Only the pathological frames from the complete test dataset are used in the evaluation.*

# **Supplementary Table 21 Model performances for stents**

| No. | Reported set | REFerence | Evaluation | Acc. (%) | Sens. (%) | Spec. (%) | PPV (%) | NPV (%) | F1 (%) | AUC-ROC | DICE | Jaccard | Definition |
| --- | --- | --- | --- | --- | --- | --- | --- | --- | --- | --- | --- | --- | --- |
|  | Classification |  |  |  |  |  |  |  |  |  |  |  |  |
| 1 | Validation folds | Core lab | Strut-level | - | Baseline  90 ± 3  Follow-up  94 ± 1 | - | Baseline  91 ± 3  Follow-up  85 ± 6 | - | - | - | - | - |  |
| 5 | Validation folds | Core lab | Strut-level | - | 91 ± 4 | - | 84 ± 8 | - | - | - | - | - |  |
| 7 | Held-out test | Expert | Strut-level | 96.5 | 92.9 | - | - | - | - | - | - | - |  |
| 9 | Held-out test | Unknown | A-line | 82.82 | - | - | - | - | - | - | - | - |  |
| 25 | Validation folds | Expert | Strut-level | - | 94 ± 3 | 90 ± 4 | - | - | - | 0.97 | - | - |  |
| 58 | Validation folds | Expert | Strut-level | - | 93.9 ± 0.7 | - | 93.2 ± 0.9 | - | - | - | - | - | Strut with coverage ≤0.3mm  True positive if distance between center of predicted and manual stent strut <94 µm |
| 58 | Validation folds | Expert | Strut-level | - | 87.4 ± 1.1 | - | 95.6 ± 1.9 | - | - | - | - | - | Strut with coverage >0.3mm  True positive if distance between center of predicted and manual stent strut <94 µm |
| 58 | Held-out test | Expert | Strut-level | - | 95.8 | - | 97.5 | - | - | - | - | - | Inner stent layer |
| 58 | Held-out test | Expert | Strut-level | - | 94.0 | - | 90.1 | - | - | - | - | - | Outer stent layer |
|  | Detection |  |  |  |  |  |  |  |  |  |  |  |  |
| 15 | Held-out test | Expert | Strut-level | - | 91.5 | 87.9 | - | - | 90 | - | - | - | BVS True positive if seed point is completely covered by ground truth |
| 36 | Held-out test | Unknown | Strut-level | - | 96.0 | - | 99.5 | - | - | - | - | - | True positive if IoU > 0.40 |
| 81 | Held-out test | Expert | Strut-level | - | 95.41 | - | 97.90 | - | 96.64 | - | - | - | True positive if IoU >0.50 |
|  | Segmentation |  |  |  |  |  |  |  |  |  |  |  |  |
| 13 | Held-out test | Expert | Strut-level | - | 90 | - | 93 | - | 91 | - | - | - | BVS |
| 42 | External test | Core lab | Pixel | - | - | - | - | - | - | - | 0.907 ± 0.039 | 0.838 ± 0.057 |  |
| 42 | External test | Core lab | Strut-level | - | - | 94.0 ± 3.9 | 94.3 ± 3.6 | - | 93.6 ± 3.8 | - | - | - | True positive if IoU >50% |
| 55 | Held-out test | Expert | Strut-level | - | 92 | - | 92 | - | - | - | - | - | True positive if Euclidian distance between predicted and manual stent strut ≤50 µm |
| 55 | Held-out test | Expert | Pixel | - | - | - | - | - | - | - | 0.86 | - | BVS |
| 91 | Held-out test | Core lab | Strut-level | - | 96.6 | 90.4 | 92.7 | - | - | - | - | - | True positive if IoU ≥0.50 |
|  | Regression |  |  |  |  |  |  |  |  |  |  |  |  |
| 80 | Held-out test | Core lab | Lesion | 84 ± 4 | 87 ± 5 | 82 ± 5 |  |  |  | 0.85 ± 0.02 |  |  | Stent expansion ≤80% |

Performance analysis of stent classification, detection, segmentation and regression in intracoronary OCT.
*Results on folds are reported as mean across folds unless reported otherwise. Results are reported as value ± standard deviation or value (95% confidence interval), if available unless reported otherwise.
ACC accuracy; AUC-ROC area under the receiver operating characteristic curve; BVS bioresorbable vascular scaffold; IoU intersection over union; NPV negative predictive value; PPV positive predictive value; SENS sensitivity; SPEC specificity*

# **Supplementary Table 22 Model performances for fractional flow reserve**

| No. | Reported set | REFERENCE | Evaluation | Acc. (%) | Sens. (%) | Spec. (%) | PPV (%) | NPV (%) | F1 (%) | AUC-ROC | DICE | Jaccard | Definition |
| --- | --- | --- | --- | --- | --- | --- | --- | --- | --- | --- | --- | --- | --- |
|  | Classification |  |  |  |  |  |  |  |  |  |  |  |  |
| 62 | Validation folds | Pressure wire | Pullback | 77.5 (68.4-84.5) | 72.9 (59.0-89.6) | 81.5 (69.2-89.6) | 77.8 (63.7-87.5) | 77.2 (64.8-86.2) | - | 0.763 (0.669-0.857) | - | - | FFR ≤ 0.80 |
| 78 | Held-out test | Pressure wire | Pullback | 91.7 | 98.3 | 61.5 | 92.1 | 88.9 | - | - | - | - | FFR ≤ 0.80 |
| 78 | External test | Pressure wire | Pullback | 83.2 | 89.6 | 70.6 | 85.7 | 77.4 | - | - | - | - | FFR ≤ 0.80 |
|  | Regression |  |  |  |  |  |  |  |  |  |  |  |  |
| 33 | Held-out test | Pressure wire | Pullback | 95.2 | 100 | 92.9 | 87.5 | 100 | - | 0.980 | - | - | FFR ≤ 0.80 |

Performance analysis of fractional flow reserve classification and regression in intracoronary OCT.
*Results on folds are reported as mean across folds unless reported otherwise. Results are reported as value ± standard deviation or value (95% confidence interval), if available unless reported otherwise.
ACC accuracy; AUC-ROC area under the receiver operating characteristic curve; FFR fractional flow reserve; NPV negative predictive value; PPV positive predictive value; SENS sensitivity; SPEC specificity*

# **Supplementary Table 23 Model performances for artifacts**

| No. | Reported set | REFERENCE | Evaluation | Acc. (%) | Sens. (%) | Spec. (%) | PPV (%) | NPV (%) | F1 (%) | AUC-ROC | DICE | Jaccard | Definition |
| --- | --- | --- | --- | --- | --- | --- | --- | --- | --- | --- | --- | --- | --- |
|  | Classification |  |  |  |  |  |  |  |  |  |  |  |  |
| 9 | Held-out test | Unknown | A-line | 95.91 | - | - | - | - | - | - | - | - | Guidewire artifact |
| 16 | Validation folds | Expert | Patch | 81.2 | - | - | - | - | - | - | - | - | Absence of layered wall |
| 71 | Validation folds | Core lab | Frame | - | 90.6 (89.7-91.4) | 87.1 (86.9-87.4) | 34.7 (34.1-35.2) | 99.2 (99.1-99.3) | - | 0.96 (0.95-0.96) | - | - | Poor image quality or guiding catheter |
| 71 | External test | Core lab | Frame | - | 99.0 (98.7-99.2) | 99.6 (99.6-99.7) | 95.7 (95.1-96.2) | 99.9 (99.9-99.9) | - | 1.00 (1.00-1.00) | - | - |  |
|  | Segmentation |  |  |  |  |  |  |  |  |  |  |  |  |
| 49 | Held-out test | Core lab | Pixel | - | 92.0 | - | 87.0 | - | - | - | 0.894 | - | Guidewire artifact |
| 70 | Validation folds (pooled) | Expert | Patch | 99.3 | 97.1 | 99.9 | 99.6 | 99.3 | 98.4 | - | - | - | Guidewire artifact or non-visible tissue |

Performance analysis of artifact classification and segmentation in intracoronary OCT.
*Results on folds are reported as mean across folds unless reported otherwise. Results are reported as value ± standard deviation or value (95% confidence interval), if available unless reported otherwise.
ACC accuracy; AUC-ROC area under the receiver operating characteristic curve; NPV negative predictive value; PPV positive predictive value; SENS sensitivity; SPEC specificity*

# **Supplementary Table 24 Model performances for other objectives**

| No. | Reported set | REFERENCE | Evaluation | Acc. (%) | | Sens. (%) | | Spec. (%) | | PPV (%) | | NPV (%) | | F1 (%) | | AUC-ROC | | DICE | Jaccard | | Definition | | |
| --- | --- | --- | --- | --- | --- | --- | --- | --- | --- | --- | --- | --- | --- | --- | --- | --- | --- | --- | --- | --- | --- | --- | --- |
|  | Classification |  |  |  |  | |  | |  | |  | |  | |  | |  | | |  | |  |  |
| 12 | Held-out test | Expert | A-line | 91 (IQR 75-98) | 92 (IQR 71-100) | | 100 (IQR 92-100) | | - | | - | | - | | - | | 0.93 (IQR 0.78-0.98) | | | - | | Healthy regions |  |
|  | Segmentation |  |  |  | |  | |  | |  | |  | |  | |  | |  |  | |  | | |
| 3 | Held-out test | Expert | Pixel | - | | 81 | | - | | 74 | | - | | - | | - | | - | - | | Mixed tissue | | |
| 60 | Held-out test | Expert | Pixel | - | | 97.06 | | - | | 96.35 | | - | | 95.70 | | - | | - | 0.9362 | | Outer contour (lumen, intima, media, adventitia) | | |

Performance analysis of other objective classification and segmentation in intracoronary OCT.
*Results are reported as value ± standard deviation or value (95% confidence interval), if available unless reported otherwise.
ACC accuracy; AUC-ROC area under the receiver operating characteristic curve; IQR interquartile range; NPV negative predictive value; PPV positive predictive value; SENS sensitivity; SPEC specificity*

# **Supplementary Table 25 Model performance for quantification**

| No. | Reported set | REFERENCE | Task | Feature | R | R^2^ | ICC | Measurement |
| --- | --- | --- | --- | --- | --- | --- | --- | --- |
|  | Lumen |  |  |  |  |  |  |  |
| 11 | Held-out test | Unknown | Segmentation | Lumen area | - | - | - | Mean prediction 5.26 mm^2^ (IQR 3.93-7.45); Mean reference standard 5.28 mm^2^ (IQR 3.88-7.45) Average error 1.38% (IQR 0.63-2.62) |
| 13 | Held-out test | Expert | Segmentation | Lumen area | - | 0.94 | - | Mean difference 0.09 mm^2^ |
| 14 | Held-out test | Expert | Segmentation | Lumen area | 0.98 | 0.96 | - | Mean difference 0.04 ± 0.3 mm^2^ |
| 21 | Test folds | Expert | Segmentation | Lumen area | - | - | - | Mean difference -0.54 mm^2^ (-1.2-0.14) |
| 28 | Test folds | Expert | Segmentation | Lumen area | 0.97 | - | - | Mean difference 0.13 mm^2^ (-0.56-0.82) |
| 34 | Validation folds | Expert | Segmentation | Lumen area | - | - | - | Mean difference 0.3680 mm^2^ (-0.5153-1.2513) |
| 34 | Validation folds | Expert | Segmentation | Lumen mean diameter | - | - | - | Mean difference 0.0614 mm (-0.0554-0.1782) |
| 64 | External test | IVUS | Segmentation | Lumen area | - | - | 0.85 (0.71-0.92) | Mean difference -0.47 ± 1.02 mm^2^ |
| 64 | External test | IVUS | Segmentation | Lumen diameter | - | - | 0.84 (0.71-0.91) | Mean difference -0.11 ± 0.23 mm |
|  | Tunica media |  |  |  |  |  |  |  |
| 49 | Held-out test | Core lab | Segmentation | Plaque burden | - | 0.98 | 0.99 (0.98-0.99) | Mean difference 0.35 ± 2.2% |
| 64 | External test | IVUS | Segmentation | Plaque burden | - | - | 0.81 (0.69-0.90) | Mean difference -3.53 ± 6.17%  AUC 0.94 (plaque burden >65%) |
| 64 | External test | IVUS | Segmentation | Vessel area | - | ^-^ | 0.72 (0.57-0.82) | Mean difference -1.94 ± 2.09 mm^2^ |
| 64 | External test | IVUS | Segmentation | Vessel diameter | - | - | 0.75 (0.62-0.85) | Mean difference -0.30 ± 0.31 mm |
|  | Lipid |  |  |  |  |  |  |  |
| 3 | Held-out test | Expert | Segmentation | Lipid area | 0.96 | 0.925 | - | Mean difference 0.06 ± 0.14 mm^2^ |
| 6 | Held-out test | Expert | Segmentation | Minimum FCT Texture features | 0.91 | - | - | - |
| 22 | Test folds | Core lab | Segmentation | Lipid arc | - | - | - | Mean prediction 152.3 ± 41.0°; mean reference standard 146.0 ± 44.1°, p<0.05 |
| 22 | Test folds | Core lab | Segmentation | Mean FCT | - | - | - | Mean prediction 0.121 ± 0.024 mm; mean reference standard 0.151 ± 0.035 mm, p<0.001 |
| 37 | Held-out test | Core lab | Classification | Lipid arc | - | - | - | Mean difference 6.5 ± 19.2^o^ |
| 37 | Held-out test | Core lab | Classification | Lipid length | - | - | - | Mean difference -8.7 ± 11.3 mm |
| 44 | Validation folds | Expert^a^ | Segmentation | Mean FCT | - | - | - | Mean prediction 0.740 mm; mean reference standard 0.739 mm Mean relative error 8.83% |
| 44 | Validation folds | Expert^a^ | Segmentation | Minimum FCT | - | - | - | Mean prediction 0.556 mm; mean reference standard 0.513 mm Mean relative error 17.46% |
| 53 | Held-out test | IVUS-NIRS | Classification | Maximum LCBI_4mm_ | 0.75 | - | - | - |
| 66 | Held-out test | Core lab | Segmentation | Lipid arc | - | 0.943 | - | Mean difference between observers using software 6.7 ± 17.3° |
| 66 | Held-out test | Core lab | Segmentation | Minimum FCT | - | 0.974 | - | Mean difference between observers using software 4.2 ± 14.6 µm |
|  | High-risk plaques |  |  |  |  |  |  |  |
| 40 | Held-out test | Expert | Classification | TCFA burden | 0.87 | - | - | - |
|  | Calcium |  |  |  |  |  |  |  |
| 3 | Held-out test | Expert | Segmentation | Calcium area | 0.96 | 0.962 | - | Mean difference 0.008 ± 0.04 mm^2^ |
| 21 | Test folds | Expert | Segmentation | Calcium arc | - | - | - | Mean difference -7^o^ (-52-37) |
| 21 | Test folds | Expert | Segmentation | Calcium thickness | - | - | - | Mean difference -0.13 mm (-0.52-0.25) |
| 21 | Test folds | Expert | Segmentation | Calcification depth | - | - | - | Mean difference 0.05 mm (-0.15-0.25) |
| 22 | Test folds | Core lab | Segmentation | Calcium arc | - | - | - | Mean prediction 88.6 ± 39.1°; mean reference standard 85.0 ± 35.5°, p<0.001 |
| 22 | Test folds | Core lab | Segmentation | Calcium depth | - | - | - | Mean prediction 0.049 ± 0.012 mm; mean reference standard 0.050 ± 0.013 mm, p≥0.05 |
| 27 | Held-out test | Expert | Classification | Calcium arc | - | - | - | Mean difference 7.70 ± 39.0° |
| 37 | Held-out test | Core lab | Classification | Calcium arc | - | - | - | Mean difference 13.2 ± 16.8° |
| 37 | Held-out test | Core lab | Classification | Calcium length | - | - | - | Mean difference 10.8 ± 20.1 mm |
| 38 | External test^b^ | NA^c^ | Segmentation | Calcium arc | - | - | - | Difference range 0.8-11.4^o^ |
| 38 | External test^b^ | NA^c^ | Segmentation | Calcium depth | - | - | - | Difference range 0.002-0.067 mm |
| 38 | External test^b^ | NA^c^ | Segmentation | Calcium thickness | - | - | - | Difference range 0.014-0.030 mm |
| 61 | Held-out test | Expert | Segmentation | Maximum calcium arc | 0.99 | - | - | Mean prediction 106.8 ± 70.7^o^; mean reference standard 104.3 ± 68.6^o^ |
| 61 | Held-out test | Expert | Segmentation | Maximum calcium thickness | 0.84 | - | - | Mean prediction 1.15 ± 0.66 mm; reference standard 1.21 ± 0.73 mm |
| 61 | Held-out test | Expert | Segmentation | Calcium score | 0.90 | - | - | Mean prediction 1.45 ± 0.96; reference standard 1.52 ± 1.03 |
| 61 | Held-out test | Expert | Segmentation | Calcium volume | 0.99 | - | - | Mean prediction 4.96 ± 4.70 mm^3^; reference standard 4.73 ± 4.30 |
| 68 | Held-out test | Expert | Segmentation | Calcium area | - | - | - | Mean difference 0.13 mm^2^ (-0.73-0.99) |
| 68 | Held-out test | Expert | Segmentation | Calcium arc | - | - | - | Mean difference 2.7 ^o^ (-23.4-28.8) |
| 68 | Held-out test | Expert | Segmentation | Calcium depth | - | - | - | Mean difference 0.01 mm (-0.14-0.16) |
| 68 | Held-out test | Expert | Segmentation | Calcium thickness | - | - | - | Mean difference 0.05 mm (-0.22-0.32) |
| 68 | Held-out test | Expert | Segmentation | Calcium volume | - | - | - | Mean difference 0.4 mm^3^ (-2.8-3.6) |
| 68 | Held-out test | Expert | Segmentation | Calcium score | - | - | - | Accuracy 86.4%, sensitivity 86.4%, PPV 87.7%, F1 score 86.2% |
|  | Fibrous |  |  |  |  |  |  |  |
| 3 | Held-out test | Expert | Segmentation | Fibrous plaque area | 0.97 | 0.949 | - | Mean difference -0.09 ± 0.13 mm^2^ |
|  | Microvessel |  |  |  |  |  |  |  |
| 75 | Held-out test | Expert | Segmentation | Microvessel area | 0.825 | - | - | Mean difference -0.017 (-0.086-0.052) |
|  | Stent |  |  |  |  |  |  |  |
| 1 | Validation folds | Core lab | Classification | Stent area | - | - | - | Mean difference 0.12 ± 0.20 mm^2^ |
| 1 | Validation folds | Core lab | Classification | Tissue area | - | - | - | Mean difference 0.11 ± 0.20 mm^2^ |
| 5 | Validation folds | Core lab | Classification | Stent area | 0.988 | - | - | - |
| 13 | Held-out test | Expert | Segmentation | Strut count | - | - | - | Mean difference -0.16 |
| 13 | Held-out test | Expert | Segmentation | Strut area | - | 0.93 | - | Mean difference 0.14 mm^2^ |
| 25 | Validation folds | Expert | Classification | Tissue coverage thickness | - | - | - | Mean difference 2.5 ± 29 µm |
| 25 | Validation folds | Expert | Classification | Lumen to strut distance | - | - | - | Mean difference 0.1 ± 2.9 µm |
| 42 | External test | Core lab | Segmentation | Minimum stent area | 0.95 | - | - | Mean difference -0.07 ± 1.15 mm^2^ |
| 42 | External test | Core lab | Segmentation | Average stent area | 0.99 | - | - | Mean difference -0.36 ± 0.42 mm^2^ |
| 42 | External test | Core lab | Segmentation | Malapposition distance | 0.98 | - | - | Mean difference 0.02 ± 0.05 mm |
| 42 | External test | Core lab | Segmentation | Tissue coverage thickness | 0.99 | - | - | Mean difference 0.00 ± 0.05 mm |
| 58 | Validation folds | Expert | Classification | Stent area | 0.962 | - | - | Mean difference -0.13 mm^2^ (-1.38-1.12) |
| 58 | Validation folds | Expert | Classification | Stent area | 0.925 | - | - | Mean difference -0.24 mm^2^ (-1.76-1.28) |
| 58 | Validation folds | Expert | Classification | Stent area | 0.918 | - | - | Mean difference -0.13 mm^2^ (-0.62-0.40) |
| 80 | Held-out test | Core lab | Regression | Post-stent lumen area | 0.94 ± 0.04 | - | - | Mean error -0.1 ± 0.7 mm^2^ |
|  | Fractional flow reserve |  |  |  |  |  |  |  |
| 33 | Held-out test | Pressure wire | Regression | Fractional flow reserve | 0.853 | - | - | Mean difference 0.01 (-0.09-0.12) |
| 78 | Held-out test | Pressure wire | Classification | Fractional flow reserve | 0.8782 | - | - | Mean absolute error 0.0344 Mean difference 0.01 ± 0.09 |
| 78 | External test | Pressure wire | Classification | Fractional flow reserve | 0.7884 | - | - | - |
|  | Mixed tissue |  |  |  |  |  |  |  |
| 3 | Held-out test | Expert | Segmentation | Plaque area | 0.96 | 0.930 | - | Mean difference -0.04 ± 0.10 mm^2^ |

Performance analysis for quantification of structures in intracoronary OCT.
*Results on folds are reported as mean across folds unless reported otherwise. Results are reported as value ± standard deviation or value (95% confidence interval), if available unless reported otherwise.
AUC area under the curve; ICC intra-class correlation; IQR interquartile range; IVUS intravascular ultrasound; IVUS-NIRS intravascular ultrasound-near-infrared spectroscopy; NA not applicable; PPV positive predictive value; R Pearson’s correlation coefficient; R^2^ coefficient of determination; TCFA Thin-cap fibroatheroma
^a^Using virtual-histology intravascular ultrasound (VH-IVUS) as reference.
^b^Only the ex vivo dataset from the complete test dataset is used in the evaluation.
^c^Assessment of correlation between repeated pullbacks of the same lesion.*

# **Supplementary Table 26 Model performances for stent-related, plaque-related or clinical outcome**

| No. | Reported set | Evaluation | Endpoint | Follow-up | Plaque feature of interest | Result |
| --- | --- | --- | --- | --- | --- | --- |
| Clinical | | | | | | |
| 63 | OPTIMAL registry | Patient | Non-culprit vessel-related MACE:  cardiac death, nonfatal acute myocardial infarction and/or ischemia-driven revascularization | 2 year | LCR | Optimal cutoff >0.33mm^2^  AUC 0.826 (0.793-0.855)  Sensitivity 92.3%  Specificity 74.4%  PPV 14.0%  NPV 99.5%  HR 19.13 (4.49-81.55) |
| 63 | OPTIMAL registry | Patient | Non-culprit vessel-related MACE:  cardiac death, nonfatal acute myocardial infarction and/or ischemia-driven revascularization | 2 year | TCFA  (lipid arc >180^o^ and minimal fibrous cap thickness ≤65µm) | Sensitivity 34.6%  Specificity 89.5%  PPV 12.9%  NPV 96.8%  HR 2.77 (1.19-6.44) |
| 63 | OPTIMAL registry | Lesion | Non-culprit vessel-related MACE. | 2 year | LCR | HR 10.72 (3.67-31.33) |
| 69 | Validation folds | Lesion | Clinically-driven revascularization and angiographic progression of coronary artery disease with a diameter >75% | 530 (310-1105) days | Vulnerable plaque  (fibrous cap thickness <100 µm, lipid arc >90°) | Odds ratio 13.526 (3.730-49.051)  AUC 0.719 |
| 69 | Validation folds | Patient | Composite of cardiac death, noncardiac death, any clinically driven coronary revascularization | 530 (310-1105) days | Vulnerable plaque  (fibrous cap thickness <100 µm, lipid arc >90°) | Odds ratio 2.295 (1.478-3.562)  AUC 0.590 |
| 77 | CLIMA | Patient | Cardiac death, any myocardial infarction and/or target-vessel revascularization | 1 year | MaxOCT-LCBI_4mm_ ≥400 | Incidence 8.2% (present) vs 4.6% (absent), p=0.017 HR 1.87 (1.12-3.12).  Multivariate HR 1.85 (1.12-3.12)  PPV 8.2%  NPV 95.4% |
| 77 | CLIMA | Patient | Cardiac death and/or target LAD segment myocardial infarction | 1 year | MaxOCT-LCBI_4mm_ ≥400 | Logrank p=0.008 HR 2.56 (1.24-5.29)  Specificity 48.55%  Sensitivity 72.97%  PPV 5.2%  NPV 97.9% |
| Stent | | | | | | |
| 67 | NA | Lesion | Neoatherosclerosis | 15 to 18 months | Fibrous cap surface area | AUC 0.901 (0.859-0.946) |
| Plaque | | | | | | |
| 50 | Validation folds | Frame | Change in lipid percentage index | 10 months | Wall thickness | AUC 0.883  Sensitivity 65.3%  Specificity 94.7% |
| 84 | Validation folds | Frame | Change in cap thickness plaque vulnerability index | 251 ± 41 days | Plaque area, plaque burden, minimal fibrous cap thickness, mean fibrous cap thickness, mean fibrous cap strain | Accuracy 90.3%  Sensitivity 56.7%  Specificity 95.8%  AUC 0.877 |

Performance analysis of stent-related, plaque-related or clinical outcome in intracoronary OCT.
*Results on folds are reported as mean across folds unless reported otherwise. Results are reported as value ± standard deviation or value (95% confidence interval), if available unless reported otherwise.
AUC area under the curve; HR hazard ratio; LCBI Lipid Core Burden Index; LCR Lipid-to-Cap Ratio; MACE major adverse cardiovascular events; NPV negative predictive value; PPV positive predictive value; TCFA thin-cap fibroatheroma*

# **Supplementary Table 27 Bias signaling questions**

| Paper | 1 | 2 | 3 | 4 | 5 | 6 | 7 | 8 | 9 | Paper | 1 | 2 | 3 | 4 | 5 | 6 | 7 | 8 | 9 |
| --- | --- | --- | --- | --- | --- | --- | --- | --- | --- | --- | --- | --- | --- | --- | --- | --- | --- | --- | --- |
| 1 | - | - | - | + | ? | + | + | - | - | 46 | - | - | + | + | + | - | - | + | - |
| 2 | - | + | - | - | ? | ? | + | + | - | 47 | - | - | + | + | + | + | NA | + | - |
| 3 | - | - | + | + | ? | - | + | + | - | 48 | - | - | - | + | ? | + | + | + | - |
| 4 | + | + | + | - | ? | - | + | - | - | 49 | + | + | + | + | ? | + | + | + | + |
| 5 | - | - | + | + | ? | + | ? | + | - | 50 | + | + | - | ? | ? | - | ? | - | - |
| 6 | - | - | - | NA | NA | NA | - | + | - | 51 | + | + | - | + | ? | + | + | + | + |
| 7 | - | - | + | + | ? | - | ? | + | - | 52 | - | - | - | + | + | - | NA | + | - |
| 8 | + | - | - | - | ? | + | ? | + | - | 53 | + | + | - | + | + | + | + | + | - |
| 9 | - | - | - | ? | ? | - | ? | + | - | 54 | - | - | - | + | ? | - | ? | + | - |
| 10 | - | + | - | - | + | + | + | + | + | 55 | - | - | + | - | - | + | + | + | - |
| 11 | + | - | + | ? | + | + | NA | + | - | 56 | - | + | + | + | + | - | + | + | - |
| 12 | - | - | - | + | ? | + | ? | + | - | 57 | - | - | - | ? | ? | - | + | + | - |
| 13 | - | - | + | NA | NA | NA | ? | + | - | 58 | - | - | + | - | - | + | - | + | - |
| 14 | - | - | + | NA | NA | NA | NA | + | - | 59 | - | - | + | + | ? | - | + | - | - |
| 15 | - | - | + | - | ? | + | ? | + | - | 60 | - | - | - | + | + | - | NA | + | - |
| 16 | + | + | + | + | ? | + | ? | - | - | 61 | - | - | + | + | + | - | - | + | - |
| 17 | + | - | - | + | ? | ? | ? | ? | - | 62 | + | + | + | NA | ? | + | ? | - | - |
| 18 | - | + | - | + | ? | + | + | + | - | 63 | + | + | + | NA | NA | NA | NA | NA | NA |
| 19 | + | - | - | + | ? | - | + | + | - | 64 | + | + | + | NA | ? | + | + | + | + |
| 20 | - | - | + | + | + | + | + | + | - | 65 | + | + | - | - | ? | ? | + | + | - |
| 21 | - | - | - | + | ? | - | + | + | - | 66 | + | + | + | + | ? | - | + | + | - |
| 22 | - | - | - | + | ? | - | + | + | - | 67 | + | + | - | NA | NA | NA | NA | NA | NA |
| 23 | - | - | - | + | + | - | - | + | - | 68 | - | - | + | + | ? | + | ? | + | - |
| 24 | - | - | - | + | - | ? | + | + | - | 69 | + | + | + | + | - | + | + | + | - |
| 25 | - | + | - | + | + | + | + | - | - | 70 | - | + | - | - | + | + | + | - | - |
| 26 | - | - | - | - | ? | ? | ? | - | - | 71 | + | + | + | + | - | + | + | + | + |
| 27 | - | - | - | + | - | + | + | + | - | 72 | - | + | - | - | ? | + | ? | + | - |
| 28 | - | - | - | + | + | - | NA | + | - | 73 | - | - | - | + | - | + | + | + | - |
| 29 | - | - | - | + | ? | + | + | - | - | 74 | + | + | + | + | + | + | + | + | - |
| 30 | - | - | - | + | + | ? | + | + | - | 75 | - | - | - | + | ? | + | ? | + | - |
| 31 | - | - | - | + | ? | - | + | + | - | 76 | + | + | + | - | - | + | + | + | + |
| 32 | - | - | + | + | + | - | - | + | - | 77 | + | + | - | NA | NA | NA | NA | NA | NA |
| 33 | + | + | + | NA | NA | + | NA | + | - | 78 | + | + | + | NA | - | + | NA | + | + |
| 34 | - | - | - | - | + | ? | NA | - | - | 80 | + | + | - | + | NA | + | NA | + | - |
| 35 | - | - | + | - | ? | + | + | - | - | 81 | - | - | - | + | + | + | - | + | - |
| 36 | - | - | - | ? | ? | - | - | + | - | 83 | - | - | - | + | + | - | + | + | - |
| 37 | - | - | + | + | ? | - | + | + | - | 84 | + | + | - | ? | ? | - | ? | - | - |
| 38 | - | - | + | + | ? | + | + | + | + | 85 | - | - | - | + | ? | ? | ? | + | - |
| 40 | + | + | + | + | + | + | + | + | + | 86 | - | + | - | + | ? | - | ? | + | - |
| 41 | - | - | - | + | - | ? | + | + | - | 87 | - | - | - | + | - | ? | + | + | - |
| 42 | - | + | + | + | ? | + | ? | + | + | 88 | - | - | + | + | + | - | NA | + | - |
| 43 | - | + | + | - | + | ? | + | - | - | 89 | - | - | - | + | ? | + | + | + | - |
| 44 | - | - | + | + | ? | - | ? | - | - | 90 | - | + | - | + | ? | - | ? | + | - |
| 45 | - | - | - | + | ? | + | + | + | - | 91 | - | - | + | + | ? | + | + | + | - |

Summary of bias signaling question results for included articles. The answers +, - and ? correspond to question answers yes, no and unknown, respectively.
*NA not applicable*

# **References**

1. Lu H, Gargesha M, Wang Z, Chamie D, Attizzani GF, Kanaya T, et al. Automatic stent detection in intravascular OCT images using bagged decision trees. Biomedical Optics Express. 2012;3(11):2809-24.

2. Ughi GJ, Adriaenssens T, Sinnaeve P, Desmet W, D'Hooge J. Automated tissue characterization of in vivo atherosclerotic plaques by intravascular optical coherence tomography images. Biomedical Optics Express. 2013;4(7):1014-30.

3. Athanasiou LS, Bourantas CV, Rigas G, Sakellarios AI, Exarchos TP, Siogkas PK, et al. Methodology for fully automated segmentation and plaque characterization in intracoronary optical coherence tomography images. Journal of biomedical optics. 2014;19(2):026009.

4. Macedo MMG, Guimaraes WVN, Galon MZ, Takimura CK, Lemos PA, Gutierrez MA. A bifurcation identifier for IV-OCT using orthogonal least squares and supervised machine learning. Computerized Medical Imaging and Graphics. 2015;46:237-48.

5. Zhao W, Jenkins MW, Linderman GC, Bezerra HG, Fujino Y, Costa MA, et al. 3-D Stent Detection in Intravascular OCT Using a Bayesian Network and Graph Search. IEEE Trans Med Imaging. 2015;34(7):1549-61.

6. He CL, Wang JQ, Huang YX, Zhu TJ, Miao YH, Li ZY. The Correlation Between Texture Features and Fibrous Cap Thickness of Lipid-Rich Atheroma Based on Optical Coherence Tomography Imaging. MOLECULAR & CELLULAR BIOMECHANICS. 2016;13(1):23-36.

7. Nam HS, Kim CS, Lee JJ, Song JW, Kim JW, Yoo H. Automated detection of vessel lumen and stent struts in intravascular optical coherence tomography to evaluate stent apposition and neointimal coverage. Medical Physics. 2016;43(4):1662-75.

8. Abdolmanafi A, Duong L, Dahdah N, Cheriet F. Deep feature learning for automatic tissue classification of coronary artery using optical coherence tomography. Biomed Opt Express. 2017;8(2):1203-20.

9. Essa E, Xie XH. Automatic segmentation of cross-sectional coronary arterial images. COMPUTER VISION AND IMAGE UNDERSTANDING. 2017;165:97-110.

10. Shalev R, Nakamura D, Nishino S, Rollins AM, Bezerra HG, Wilson DL, et al. Automated volumetric intravascular plaque classification using optical coherence tomography. AI Magazine. 2017;38(1):61-72.

11. Yong YL, Tan LK, McLaughlin RA, Chee KH, Liew YM. Linear-regression convolutional neural network for fully automated coronary lumen segmentation in intravascular optical coherence tomography. Journal of biomedical optics. 2017;22(12):1-9.

12. Zahnd G, Hoogendoorn A, Combaret N, Karanasos A, Pery E, Sarry L, et al. Contour segmentation of the intima, media, and adventitia layers in intracoronary OCT images: application to fully automatic detection of healthy wall regions. International Journal of Computer Assisted Radiology and Surgery. 2017;12(11):1923-36.

13. Amrute JM, Athanasiou LS, Rikhtegar F, Hern, ez JMD, Camarero TG, et al. Polymeric endovascular strut and lumen detection algorithm for intracoronary optical coherence tomography images. JOURNAL OF BIOMEDICAL OPTICS. 2018;23(3).

14. Athanasiou L, Nezami FR, Galon MZ, Lopes AC, Lemos PA, Hern DLT, et al. Optimized Computer-Aided Segmentation and Three-Dimensional Reconstruction Using Intracoronary Optical Coherence Tomography. IEEE Journal of Biomedical and Health Informatics. 2018;22(4):1168-76.

15. Cao Y, Jin QH, Lu YF, Jing J, Chen YD, Yin QY, et al. Automatic analysis of bioresorbable vascular scaffolds in intravascular optical coherence tomography images. BIOMEDICAL OPTICS EXPRESS. 2018;9(6):2495-510.

16. Chen Z, Pazdernik M, Zhang HH, Wahle A, Guo ZH, Bedanova H, et al. Quantitative 3D Analysis of Coronary Wall Morphology in Heart Transplant Patients: OCT-Assessed Cardiac Allograft Vasculopathy Progression. MEDICAL IMAGE ANALYSIS. 2018;50:95-105.

17. Huang Y, He C, Wang J, Miao Y, Zhu T, Zhou P, et al. Intravascular optical coherence tomography image segmentation based on support vector machine algorithm. MCB Molecular and Cellular Biomechanics. 2018;15(2):117-25.

18. Kolluru C, Prabhu D, Gharaibeh Y, Bezerra H, Guagliumi G, Wilson D. Deep neural networks for A-line-based plaque classification in coronary intravascular optical coherence tomography images. Journal of Medical Imaging. 2018;5(4):044504.

19. Abdolmanafi A, Cheriet F, Duong L, Ibrahim R, Dahdah N. An automatic diagnostic system of coronary artery lesions in Kawasaki disease using intravascular optical coherence tomography imaging. J Biophotonics. 2020;13(1):e201900112.

20. Gessert N, Lutz M, Heyder M, Latus S, Leistner DM, Abdelwahed YS, et al. Automatic Plaque Detection in IVOCT Pullbacks Using Convolutional Neural Networks. IEEE Transactions on Medical Imaging. 2019;38(2):426-34.

21. Gharaibeh Y, Prabhu D, Kolluru C, Lee J, Zimin V, Bezerra H, et al. Coronary calcification segmentation in intravascular OCT images using deep learning: Application to calcification scoring. Journal of Medical Imaging. 2019;6(4):045002.

22. Lee J, Prabhu D, Kolluru C, Gharaibeh Y, Zimin VN, Bezerra HG, et al. Automated plaque characterization using deep learning on coronary intravascular optical coherence tomographic images. Biomedical Optics Express. 2019;10(12):6497-515.

23. Li LC, Jia T. Optical Coherence Tomography Vulnerable Plaque Segmentation Based on Deep Residual U-Net. REVIEWS IN CARDIOVASCULAR MEDICINE. 2019;20(3):171-7.

24. Liu R, Zhang YZ, Zheng YT, Liu YQ, Zhao Y, Yi L. Automated Detection of Vulnerable Plaque for Intravascular Optical Coherence Tomography Images. CARDIOVASCULAR ENGINEERING AND TECHNOLOGY. 2019;10(4):590-603.

25. Lu H, Lee J, Ray S, Tanaka K, Bezerra HG, Rollins AM, et al. Automated stent coverage analysis in intravascular oct (IVOCT) image volumes using a support vector machine and mesh growing. Biomedical Optics Express. 2019;10(6):2809-28.

26. Miyagawa M, Costa MGF, Gutierrez MA, Costa J, Costa CFF. Detecting Vascular Bifurcation in IVOCT Images Using Convolutional Neural Networks With Transfer Learning. IEEE ACCESS. 2019;7:66167-75.

27. Prabhu D, Bezerra HG, Kolluru C, Gharaibeh Y, Mehanna E, Wu H, et al. Automated A-line coronary plaque classification of intravascular optical coherence tomography images using handcrafted features and large datasets. Journal of Biomedical Optics. 2019;24(10).

28. Tang J, Lan Y, Chen S, Zhong Y, Huang C, Peng Y, Liu Q, Cheng Y, Chen F, Che W. Lumen Contour Segmentation in IVOCT Based on N-type CNN. IEEE ACCESS. 2019;7:135573-81

29. Yan Q, Xu M, Wong DWK, Taruya A, Tanaka A, Liu J, et al. Automatic fibroatheroma identification in intravascular optical coherence tomography volumes. JOURNAL OF AMBIENT INTELLIGENCE AND HUMANIZED COMPUTING.

30. Yang J, Zhang B, Wang H, Lin F, Han Y, Liu X. Automated characterization and classification of coronary atherosclerotic plaques for intravascular optical coherence tomography. Biocybernetics and Biomedical Engineering. 2019;39(3):719-27.

31. Zhang H, Wang G, Li Y, Lin F, Han Y, Wang H. Automatic plaque segmentation in coronary optical coherence tomography images. International Journal of Pattern Recognition and Artificial Intelligence. 2019;33(14).

32. Zhang C, Li H, Guo X, Molony D, Guo X, Samady H, et al. Convolution neural networks and support vector machines for automatic segmentation of intracoronary optical coherence tomography. MCB Molecular and Cellular Biomechanics. 2019;16(2):153-61.

33. Cha JJ, Son TD, Ha J, Kim JS, Hong SJ, Ahn CM, et al. Optical coherence tomography-based machine learning for predicting fractional flow reserve in intermediate coronary stenosis: a feasibility study. SCIENTIFIC REPORTS. 2020;10(1).

34. Gao Z, Chung J, Abdelrazek M, Leung S, Hau WK, Xian Z, et al. Privileged Modality Distillation for Vessel Border Detection in Intracoronary Imaging. IEEE Transactions on Medical Imaging. 2020;39(5):1524-34.

35. He C, Wang J, Yin Y, Li Z. Automated classification of coronary plaque calcification in OCT pullbacks with 3D deep neural networks. Journal of biomedical optics. 2020;25(9).

36. Jiang XL, Zeng YQ, Xiao SX, He SJ, Ye CZ, Qi Y, et al. Automatic Detection of Coronary Metallic Stent Struts Based on YOLOv3 and R-FCN. COMPUTATIONAL AND MATHEMATICAL METHODS IN MEDICINE. 2020;2020.

37. Lee J, Prabhu D, Kolluru C, Gharaibeh Y, Zimin VN, Dallan LAP, et al. Fully automated plaque characterization in intravascular OCT images using hybrid convolutional and lumen morphology features. Scientific reports. 2020;10(1):2596.

38. Lee J, Gharaibeh Y, Kolluru C, Zimin VN, Dallan LAP, Kim JN, et al. Segmentation of Coronary Calcified Plaque in Intravascular OCT Images Using a Two-Step Deep Learning Approach. IEEE Access. 2020;8:225581-93.

39. Lu H, Lee J, Jakl M, Wang Z, Cervinka P, Bezerra HG, et al. Application and Evaluation of Highly Automated Software for Comprehensive Stent Analysis in Intravascular Optical Coherence Tomography (vol 10, 2150, 2020). SCIENTIFIC REPORTS. 2020;10(1).

40. Min HS, Yoo JH, Kang SJ, Lee JG, Cho H, Lee PH, et al. Detection of optical coherence tomography-defined thin-cap fibroatheroma in the coronary artery using deep learning. EuroIntervention : journal of EuroPCR in collaboration with the Working Group on Interventional Cardiology of the European Society of Cardiology. 2020;16(5):404-12.

41. Wang J. OCT Image Recognition of Cardiovascular Vulnerable Plaque Based on CNN. IEEE Access. 2020;8:140767-76.

42. Wu P, Gutierrez-Chico JL, Tauzin H, Yang W, Li YG, Yu W, et al. Automatic stent reconstruction in optical coherence tomography based on a deep convolutional model. BIOMEDICAL OPTICS EXPRESS. 2020;11(6):3374-94.

43. Yang S, Yoon HJ, Yazdi SJM, Lee JH. A novel automated lumen segmentation and classification algorithm for detection of irregular protrusion after stents deployment. International Journal of Medical Robotics and Computer Assisted Surgery. 2020;16(1).

44. Zhang CN, Guo XP, Guo XY, Molony D, Li HG, Samady H, et al. Machine Learning Model Comparison for Automatic Segmentation of Intracoronary Optical Coherence Tomography and Plaque Cap Thickness Quantification. CMES-COMPUTER MODELING IN ENGINEERING & SCIENCES. 2020;123(2):631-46.

45. Abdolmanafi A, Duong L, Ibrahim R, Dahdah N. A deep learning-based model for characterization of atherosclerotic plaque in coronary arteries using optical coherence tomography images. MEDICAL PHYSICS. 2021;48(7):3511-24.

46. Avital Y, Madar A, Arnon S, Koifman E. Identification of coronary calcifications in optical coherence tomography imaging using deep learning. Scientific reports. 2021;11(1):11269.

47. Balaji A, Kelsey LJ, Majeed K, Schultz CJ, Doyle BJ. Coronary artery segmentation from intravascular optical coherence tomography using deep capsules. Artificial Intelligence in Medicine. 2021;116:102072.

48. Cheimariotis GA, Riga M, Haris K, Toutouzas K, Katsaggelos AK, Maglaveras N. Automatic classification of a-lines in intravascular oct images using deep learning and estimation of attenuation coefficients. Applied Sciences (Switzerland). 2021;11(16).

49. Chu M, Jia HB, Gutierrez-Chico JL, Maehara A, Ali ZA, Zeng XL, et al. Artificial intelligence and optical coherence tomography for the automatic characterisation of human atherosclerotic plaques. EUROINTERVENTION. 2021;17(1):41-+.

50. Guo X, Maehara A, Matsumura M, Wang L, Zheng J, Samady H, et al. Predicting plaque vulnerability change using intravascular ultrasound + optical coherence tomography image-based fluid-structure interaction models and machine learning methods with patient follow-up data: a feasibility study. Biomed Eng Online. 2021;20(1):34.

51. Holmberg O, Lenz T, Koch V, Alyagoob A, Utsch L, Rank A, et al. Histopathology-Based Deep-Learning Predicts Atherosclerotic Lesions in Intravascular Imaging. FRONTIERS IN CARDIOVASCULAR MEDICINE. 2021;8.

52. Huang C, Lan Y, Xu G, Zhai X, Wu J, Lin F, et al. A Deep Segmentation Network of Multi-Scale Feature Fusion Based on Attention Mechanism for IVOCT Lumen Contour. IEEE/ACM transactions on computational biology and bioinformatics. 2021;18(1):62-9.

53. Isidori F, Lella E, Marco V, Albertucci M, Ozaki Y, La Manna A, et al. Adoption of a new automated optical coherence tomography software to obtain a lipid plaque spread-out plot. International Journal of Cardiovascular Imaging. 2021;37(11):3129-35.

54. Kolluru C, Lee J, Gharaibeh Y, Bezerra HG, Wilson DL. Learning with Fewer Images via Image Clustering: Application to Intravascular OCT Image Segmentation. IEEE Access. 2021;9:37273-80.

55. Lau YS, Tan LK, Chan CK, Chee KH, Liew YM. Automated segmentation of metal stent and bioresorbable vascular scaffold in intravascular optical coherence tomography images using deep learning architectures. Phys Med Biol. 2021 Dec 31;66(24). doi: 10.1088/1361-6560/ac4348. PMID: 34911053.

56. Shibutani H, Fujii K, Ueda D, Kawakami R, Imanaka T, Kawai K, et al. Automated classification of coronary atherosclerotic plaque in optical frequency domain imaging based on deep learning. Atherosclerosis. 2021;328:100-5.

57. Sun CY, Hong XJ, Shi S, Shen ZY, Zhang HD, Zhou LX. Cascade Faster R-CNN Detection for Vulnerable Plaques in OCT Images. IEEE Access. 2021;9:24697-704.

58. Yang GQ, Mehanna E, Li C, Zhu HY, He C, Lu F, et al. Stent detection with very thick tissue coverage in intravascular OCT. BIOMEDICAL OPTICS EXPRESS. 2021;12(12):7500-16.

59. Yin YF, He CL, Xu B, Li ZY. Coronary Plaque Characterization From Optical Coherence Tomography Imaging With a Two-Pathway Cascade Convolutional Neural Network Architecture. FRONTIERS IN CARDIOVASCULAR MEDICINE. 2021;8.

60. Cao XY, Jiang PL, Gao DF. A TWO-STAGE EXTRACTION NETWORK FOR THE OPTICAL COHERENCE TOMOGRAPHY CONTOUR. JOURNAL OF MECHANICS IN MEDICINE AND BIOLOGY. 2022;22(8).

61. Chen T, Yu H, Jia HB, Dai JN, Fang C, Ma LJ, et al. Automatic assessment of calcified plaque and nodule by optical coherence tomography adopting deep learning model. INTERNATIONAL JOURNAL OF CARDIOVASCULAR IMAGING. 2022;38(11):2501-10.

62. Hatfaludi CA, Tache IA, Ciușdel CF, Puiu A, Stoian D, Itu LM, et al. Towards a Deep-Learning Approach for Prediction of Fractional Flow Reserve from Optical Coherence Tomography. Applied Sciences (Switzerland). 2022;12(14).
63. Hong H, Jia H, Zeng M, Gutierrez-Chico JL, Wang Y, Zeng X, et al. Risk Stratification in Acute Coronary Syndrome by Comprehensive Morphofunctional Assessment With Optical Coherence Tomography. JACC: Asia. 2022;2(4):460-72.

64. Huang J, Tu S, Masuda S, Ninomiya K, Dijkstra J, Chu M, et al. Plaque burden estimated from optical coherence tomography with deep learning: In vivo validation using co-registered intravascular ultrasound. Catheter Cardiovasc Interv. 2022.

65. Lee J, Kim JN, Gomez-Perez L, Gharaibeh Y, Motairek I, Pereira GTR, et al. Automated Segmentation of Microvessels in Intravascular OCT Images Using Deep Learning. Bioengineering. 2022;9(11).

66. Lee J, Pereira GTR, Gharaibeh Y, Kolluru C, Zimin VN, Dallan LAP, et al. Automated analysis of fibrous cap in intravascular optical coherence tomography images of coronary arteries. Scientific reports. 2022;12(1):21454.

67. Lee J, Pereira GTR, Motairek I, Kim JN, Zimin VN, Dallan LAP, et al. Neoatherosclerosis prediction using plaque markers in intravascular optical coherence tomography images. Frontiers in Cardiovascular Medicine. 2022;9:1079046.

68. Li C, Jia HB, Tian JW, He C, Lu F, Li KW, et al. Comprehensive Assessment of Coronary Calcification in Intravascular OCT Using a Spatial-Temporal Encoder-Decoder Network. IEEE TRANSACTIONS ON MEDICAL IMAGING. 2022;41(4):857-68.

69. Niioka H, Kume T, Kubo T, Soeda T, Watanabe M, Yamada R, et al. Automated diagnosis of optical coherence tomography imaging on plaque vulnerability and its relation to clinical outcomes in coronary artery disease. SCIENTIFIC REPORTS. 2022;12(1).

70. Olender ML, Niu YA, Marlevi D, Edelman ER, Nezami FR. Impact and implications of mixed plaque class in automated characterization of complex atherosclerotic lesions. COMPUTERIZED MEDICAL IMAGING AND GRAPHICS. 2022;97.

71. Park S, Araki M, Nakajima A, Lee H, Fuster V, Ye JC, et al. Enhanced Diagnosis of Plaque Erosion by Deep Learning in Patients With Acute Coronary Syndromes. JACC: Cardiovascular Interventions. 2022;15(20):2020-31.

72. Rico-Jimenez JJ, Jo JA. Rapid lipid-laden plaque identification in intravascular optical coherence tomography imaging based on time-series deep learning. Journal of biomedical optics. 2022;27(10).

73. Shi PW, Xin JM, Zheng NN. A-line-based thin-cap fibroatheroma detection with multi-view IVOCT images using multi-task learning and contrastive learning. JOURNAL OF THE OPTICAL SOCIETY OF AMERICA A-OPTICS IMAGE SCIENCE AND VISION. 2022;39(12):2298-306.

74. Sun HY, Zhao C, Qin YH, Li C, Jia HB, Yu B, et al. In vivo detection of plaque erosion by intravascular optical coherence tomography using artificial intelligence. BIOMEDICAL OPTICS EXPRESS. 2022;13(7):3922-38.

75. Wu XJ, Zhang YQ, Zhang P, Hui H, Jing J, Tian F, et al. Structure attention co-training neural network for neovascularization segmentation in intravascular optical coherence tomography. MEDICAL PHYSICS. 2022;49(3):1723-38.

76. Araki M, Park S, Nakajima A, Lee H, Ye JC, Jang IK. Diagnosis of coronary layered plaque by deep learning. Scientific reports. 2023;13(1):2432.

77. Biccire FG, Budassi S, Ozaki Y, Boi A, Romagnoli E, Di Pietro R, et al. Optical coherence tomography-derived lipid core burden index and clinical outcomes: results from the CLIMA registry. European heart journal Cardiovascular Imaging. 2023;24(4):437-45.

78. Cha JJ, Nguyen NL, Tran C, Shin WY, Lee SG, Lee YJ, et al. Assessment of fractional flow reserve in intermediate coronary stenosis using optical coherence tomography-based machine learning. Frontiers in Cardiovascular Medicine. 2023;10:1082214.

79. Cioffi GM, Pinilla-Echeverri N, Sheth T, Sibbald MG. Does artificial intelligence enhance physician interpretation of optical coherence tomography: insights from eye tracking. Front Cardiovasc Med. 2023 Dec 8;10:1283338. doi: 10.3389/fcvm.2023.1283338. PMID: 38144364; PMCID: PMC10739524.

80. Gharaibeh Y, Lee J, Zimin VN, Kolluru C, Dallan LAP, Pereira GTR, Vergara-Martel A, Kim JN, Hoori A, Dong P, Gamage PT, Gu L, Bezerra HG, Al-Kindi S, Wilson DL. Prediction of stent under-expansion in calcified coronary arteries using machine learning on intravascular optical coherence tomography images. Sci Rep. 2023 Oct 23;13(1):18110. doi: 10.1038/s41598-023-44610-9. PMID: 37872298; PMCID: PMC10593923.
81. Han T, Xia W, Tao K, Wang W, Gao J, Ding X, Zhong H, Liu R, Dou S, Liu Z, Kuang H, Hua J, Xu K. Automatic stent struts detection in optical coherence tomography based on a multiple attention convolutional model. Phys Med Biol. 2023 Dec 22;69(1). doi: 10.1088/1361-6560/ad111c. PMID: 38035376.
82. Lee J, Kim JN, Gharaibeh Y, Zimin VN, Dallan LAP, Pereira GTR, et al. OCTOPUS – Optical coherence tomography plaque and stent analysis software. Heliyon. 2023;9(2).

83. Liu SJ, Xin JM, Wu JY, Deng YY, Su RS, Niessen WJ, et al. Multi-view Contour-constrained Transformer Network for Thin-cap Fibroatheroma Identification. NEUROCOMPUTING. 2023;523:224-34.

84. Lv R, Wang L, Maehara A, Matsumura M, Guo XY, Samady H, et al. Combining IVUS plus OCT Data, Biomechanical Models and Machine Learning Method for Accurate Coronary Plaque Morphology Quantification and Cap Thickness and Stress/Strain Index Predictions. JOURNAL OF FUNCTIONAL BIOMATERIALS. 2023;14(1).

85. Oikawa R, Doi A, Ishida M, Chakraborty B. Automatic detection and visualization system for coronary artery calcification using optical frequency domain imaging. ARTIFICIAL LIFE AND ROBOTICS.

86. Ren XB, Zhao YX, Fan JB, Wu HY, Chen Q, Kubo T. Semantic segmentation of superficial layer in intracoronary optical coherence tomography based on cropping-merging and deep learning. INFRARED PHYSICS & TECHNOLOGY. 2023;129.

87. Shi PW, Xin JM, Wu JY, Deng YY, Cai ZT, Du SY, et al. Detection of thin-cap fibroatheroma in IVOCT images based on weakly supervised learning and domain knowledge. JOURNAL OF BIOPHOTONICS.

88. Shi P, Xin J, Du S, Wu J, Deng Y, Cai Z, Zheng N. Automatic lumen and anatomical layers segmentation in IVOCT images using meta learning. J Biophotonics. 2023 Sep;16(9):e202300059. doi: 10.1002/jbio.202300059. Epub 2023 Jun 14. PMID: 37289201.

89. Tang H, Zhang Z, He Y, Shen J, Zheng J, Gao W, et al. Automatic classification and segmentation of atherosclerotic plaques in the intravascular optical coherence tomography (IVOCT). Biomedical Signal Processing and Control. 2023;85:104888.

90. Wang Z, Zheng J, Jiang P, Gao D. Sk-Conv and SPP-based UNet for lesion segmentation of coronary optical coherence tomography. Technology and health care : official journal of the European Society for Engineering and Medicine. 2023.

91. Wu P, Qiao YC, Chu M, Zhang S, Bai JF, Gutierrez-Chico JL, et al. Reciprocal assistance of intravascular imaging in three-dimensional stent reconstruction: Using cross-modal translation based on disentanglement representation. COMPUTERIZED MEDICAL IMAGING AND GRAPHICS. 2023;104.
